# STING Agonist‐Modified Tumor Targeting Photosensitizer Remodels Cancer‐Associated Fibroblasts to Potentiate Photoimmunotherapy in Pancreatic Cancer

**DOI:** 10.1002/advs.202520547

**Published:** 2025-12-27

**Authors:** Yixuan Zhang, Shankun Yao, Yihan Zhao, Saisai Zhu, Wen Fang, Ranran Yu, Naiwen Shi, Ruixin Zhang, Yin Zhang, Jiawei Liang, Ziying Zhang, Shijin Xu, Ziwei Zhang, Jiajun Zhang, Yukun Li, Xiaoxuan Han, Yuncong Chen, Shu Zhang, Ying Lv

**Affiliations:** ^1^ Department of Gastroenterology Nanjing Drum Tower Hospital The Affiliated Hospital of Nanjing University Medical School Nanjing China; ^2^ State Key Laboratory of Coordination Chemistry School of Chemistry and Chemical Engineering Chemistry and Biomedicine Innovation Center (ChemBIC) ChemBioMed Interdisciplinary Research Center Nanjing University Nanjing China; ^3^ Center of Hepatobiliary Pancreatic Disease Xuzhou Central Hospital Southeast University Xuzhou China; ^4^ State Key Laboratory of Pharmaceutical Biotechnology, School of Life Sciences Nanjing University Nanjing China; ^5^ Department of Gastroenterology Nanjing Drum Tower Hospital Clinical College of Nanjing University of Chinese Medicine Nanjing University of Chinese Medicine Nanjing China; ^6^ Department of Gastroenterology Nanjing Drum Tower Hospital The Affiliated Hospital of Nanjing Medical University Nanjing China; ^7^ Department of Cardiothoracic Surgery Nanjing Drum Tower Hospital Medical School Nanjing University Nanjing China

**Keywords:** cancer‐associated fibroblasts, carrier‐free nanodrug, pancreatic cancer, photoimmunotherapy, STING agonist

## Abstract

Pancreatic cancer (PC) features dense stromal barriers and profound immune exclusion, rendering it largely unresponsive to immunotherapy. Here, we present ICyM2, a carrier‐free and esterase‐responsive nanoaggregate rationally designed by covalently linking the mitochondria‐targeting photosensitizer ICyOH with the non‐nucleotide STING agonist MSA‐2. This single‐molecule construct enables high drug loading, uniform nanoaggregate formation, and preferential tumor accumulation, thereby minimizing hepatic off‐target toxicity while allowing spatiotemporally controlled activation in the tumor microenvironment. Upon intratumoral activation, ICyOH disrupts cancer‐associated fibroblast (CAF)–mediated stromal barriers and triggers immunogenic cell death (ICD), which facilitates deep penetration of MSA‐2. Subsequently, MSA‐2 amplifies ICD‐derived antigenic signaling, activates the STING pathway, promotes dendritic cell maturation, reprograms macrophages toward an M1‐like phenotype, and enhances cytotoxic T‐cell infiltration. In vivo, ICyM2 elicits potent tumor regression, establishes durable immune memory that suppresses lung metastasis, and converts immunologically “cold” tumors into “hot.” Moreover, ICyM2 synergizes with PD‐1 blockade to further strengthen antitumor immunity without observable systemic toxicity. Collectively, ICyM2 integrates stromal remodeling and immune activation within a single, carrier‐free platform, providing a clinically translatable photo‐immunotherapeutic strategy to overcome stromal and immune resistance in pancreatic cancer.

## Graphical abstract

This carrier‐free, esterase‐responsive nanoaggregate integrates a mitochondria‐targeting photosensitizer with a non‐nucleotide STING agonist to achieve localized photoactivation and immune reprogramming. ICyM2 remodels tumor stroma, induces potent immunogenic cell death, and triggers intratumoral STING activation, collectively revitalizing the immunosuppressed pancreatic tumor microenvironment and converting “cold” tumors into “hot” ones with durable antitumor immunity.

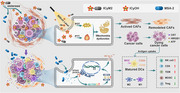

## 1 Introduction

Pancreatic cancer (PC) remains one of the most aggressive solid tumors, with a dismal 5‐year survival rate of less than 10%, making it the fourth leading cause of cancer‐related deaths globally [1, 2]. Despite advances in multimodal treatment strategies, the prognosis of PC has not been substantially improved [3]. Immunotherapy has transformed the treatment landscape of various malignancies by harnessing the immune system to eradicate tumors [4, 5, 6], yet its efficacy in PC remains limited. A defining histopathological feature of PC is its pronounced desmoplastic stroma, largely orchestrated by cancer‐associated fibroblasts (CAFs) [7, 8]. CAFs secrete abundant extracellular matrix (ECM) proteins, such as collagen, forming a dense physical barrier that not only hinders drug penetration but also restricts immune cell infiltration [9, 10]. Beyond acting as a physical blockade, CAFs engage in extensive crosstalk with immune cells, driving immunosuppressive signaling and shaping an immune‐excluded tumor microenvironment (TME) [11]. Collectively, these features underpin the “cold” immune phenotype of PC and constitute a major obstacle to immunotherapy. Therefore, strategies aimed at remodeling CAFs‐driven stromal architecture hold great promise for reversing immunosuppression and improving the clinical efficacy of immunotherapy in pancreatic cancer.

Photodynamic therapy (PDT) utilizes photosensitizers to generate reactive oxygen species (ROS) upon light irradiation, enabling precise tumor ablation with minimal invasiveness and a low risk of acquired drug resistance, and has thus attracted considerable attention as a promising therapeutic modality [12, 13]. Indocyanine green (ICG), a prototypical heptamethine cyanine dye, has been approved by the U.S. Food and Drug Administration (FDA) for near‐infrared (NIR) imaging owing to its excellent photophysical properties and favorable safety profile [14]. Inspired by the clinical success of ICG, heptamethine cyanine derivatives have become an important foundation for designing NIR photosensitizers. However, their inherently low intersystem crossing (ISC) efficiency limits ROS production, reducing PDT potency [15]. Rational structural modifications, such as heavy atom incorporation, can substantially enhance ISC efficiency, increase singlet oxygen (^1^O_2) quantum yield, and promote extracellular matrix degradation [16]. This can remodel the dense stromal barrier, improving oxygen and drug penetration and facilitating reactive oxygen species (ROS) diffusion to reinforce photosensitization [16]. Moreover, PDT has been reported to induce immunogenic cell death (ICD) in tumor cells, thereby releasing tumor‐associated antigens and initiating antitumor immunity [13].

Despite this dual potential, photosensitizer‐based PDT alone remains insufficient to achieve durable therapeutic outcomes. The cytotoxic effects of ROS are confined to the irradiated region, limiting systemic antitumor immunity [17]. Although PDT‐induced ICD triggers the release of damage‐associated molecular patterns (DAMPs) and subsequent antigen‐presenting cell (APC) recruitment, this immune activation is often too weak to elicit robust innate and adaptive responses [18, 19]. To address these limitations, the incorporation of immunoadjuvants has emerged as a critical strategy to potentiate photoimmunotherapy [20, 21, 22]. Among these, activation of the stimulator of interferon genes (STING) pathway has gained significant attention for its ability to enhance tumor immunogenicity and drive systemic antitumor immunity [23]. MSA‐2, a novel non‐nucleotide small‐molecule STING agonist discovered by Addona and colleagues, demonstrates potent immunostimulatory activity by promoting dendritic cell maturation and suppressing macrophage polarization toward an immunosuppressive M2 phenotype [24]. However, systemic intravenous delivery of MSA‐2 carries a high risk of uncontrolled inflammation and severe systemic toxicity, posing major obstacles to clinical translation [25]. Nanocarrier‐based strategies, including liposomes and polymeric nanoparticles, have been widely explored to deliver STING agonists, aiming to enhance tumor targeting and reduce systemic toxicity. These approaches have shown promising potential, but often face challenges such as particle heterogeneity, reproducibility issues, and formulation complexity, which can hinder their clinical translation [26, 27, 28]. Therefore, there is a clear need for an integrated strategy that not only ensures efficient and safe STING agonist delivery, but also leverages the stromal‐remodeling potential of PDT to enhance immune activation and achieve durable therapeutic outcomes.

**SCHEME 1:**
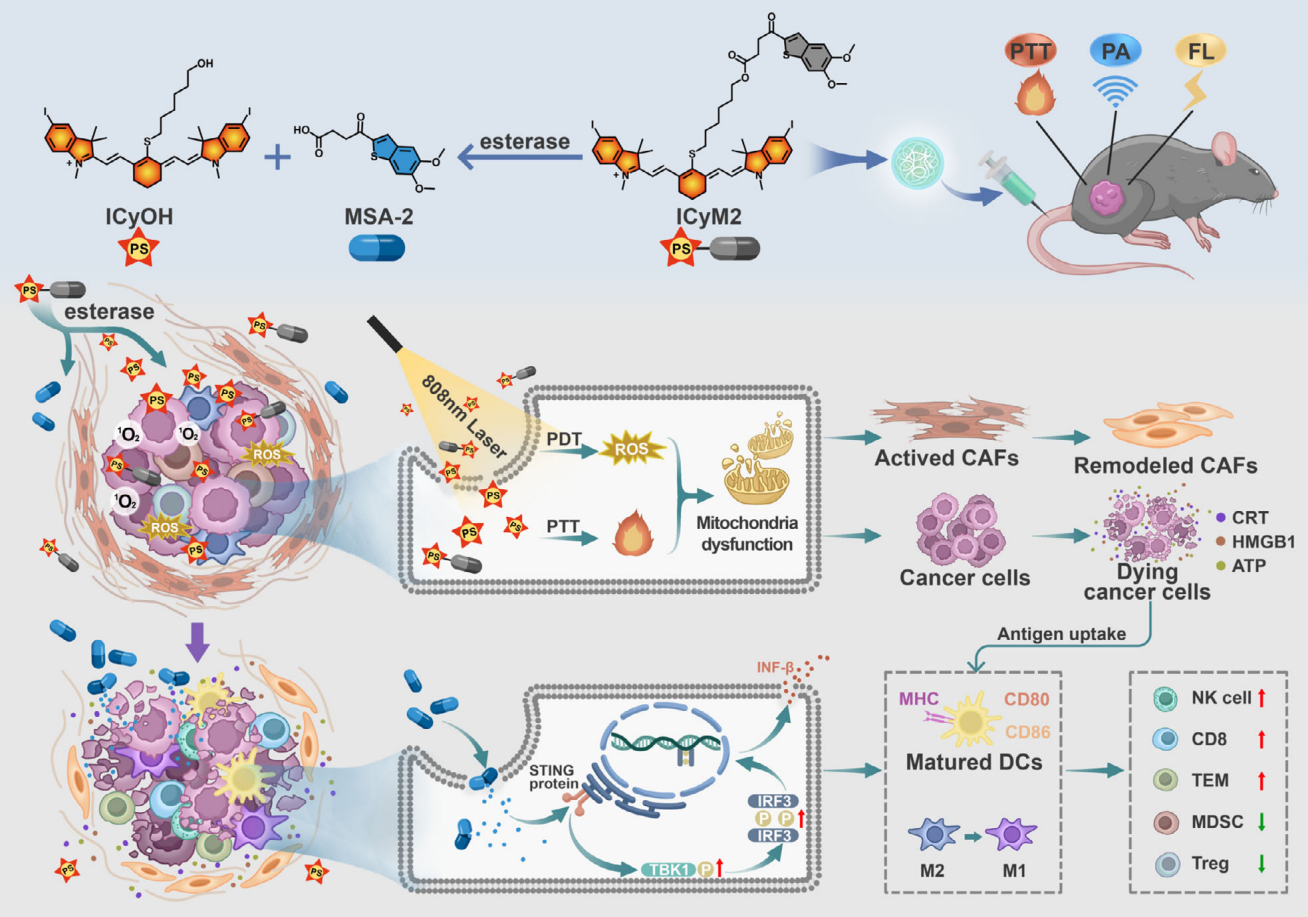
The carrier‐free and esterase‐responsive nanoaggregate is formed by covalently linking the photosensitizer ICyOH with the non‐nucleotide STING agonist MSA‐2. Upon esterase cleavage and 808 nm laser irradiation, ICyOH induces stromal remodeling and immunogenic cell death (ICD) in tumor cells, facilitating the deep penetration and sequential activation of MSA‐2. The released MSA‐2 amplifies ICD‐derived antigenic signals, activates the STING pathway, promotes dendritic‐cell maturation and macrophage repolarization, and enhances T‐cell infiltration, thereby converting immunologically “cold” tumors into “hot” ones with durable antitumor immunity.

To address these challenges, carrier‐free nanodrugs have recently emerged as promising alternatives, offering high drug loading capacity, reduced carrier‐related toxicity, and simple preparation methods, as demonstrated in recent studies [29, 30]. To further enhance both photodynamic and immunomodulatory performance, ICyM2 was rationally engineered to target mitochondria. Mitochondria are highly sensitive to oxidative stress and represent optimal subcellular sites for amplifying PDT efficacy [31], which is particularly advantageous in the hypoxic and stroma‐rich pancreatic tumor microenvironment, where conventional PDT is often limited. Building on this concept, we developed ICyM2, an esterase‐responsive carrier‐free prodrug nanoaggregate constructed by covalently linking the mitochondria‐targeting photosensitizer ICyOH with the non‐nucleotide STING agonist MSA‐2. This rational single‐molecule design enables stable nanoaggregate formation, preferential tumor accumulation, and tumor microenvironment‐specific activation. Upon activation, ICyOH disrupts CAF‐mediated stromal barriers and induces immunogenic cell death (ICD) in tumor cells, which facilitates MSA‐2 penetration. Subsequently, MSA‐2 amplifies antigenic signaling, activates the STING pathway, and promotes dendritic cell maturation and macrophage reprogramming, together eliciting potent innate and adaptive immune responses. Collectively, ICyM2 offers a clinically translatable photo‐immunotherapeutic strategy that integrates stromal remodeling, targeted immunoadjuvant delivery, and durable immune activation to overcome stromal and immune resistance in pancreatic cancer (Scheme 1).

## 2 Results and Discussion

### 2.1 CAFs Form a Compact Stromal Barrier and Modulate Myeloid Cell Function

**FIGURE 1:**
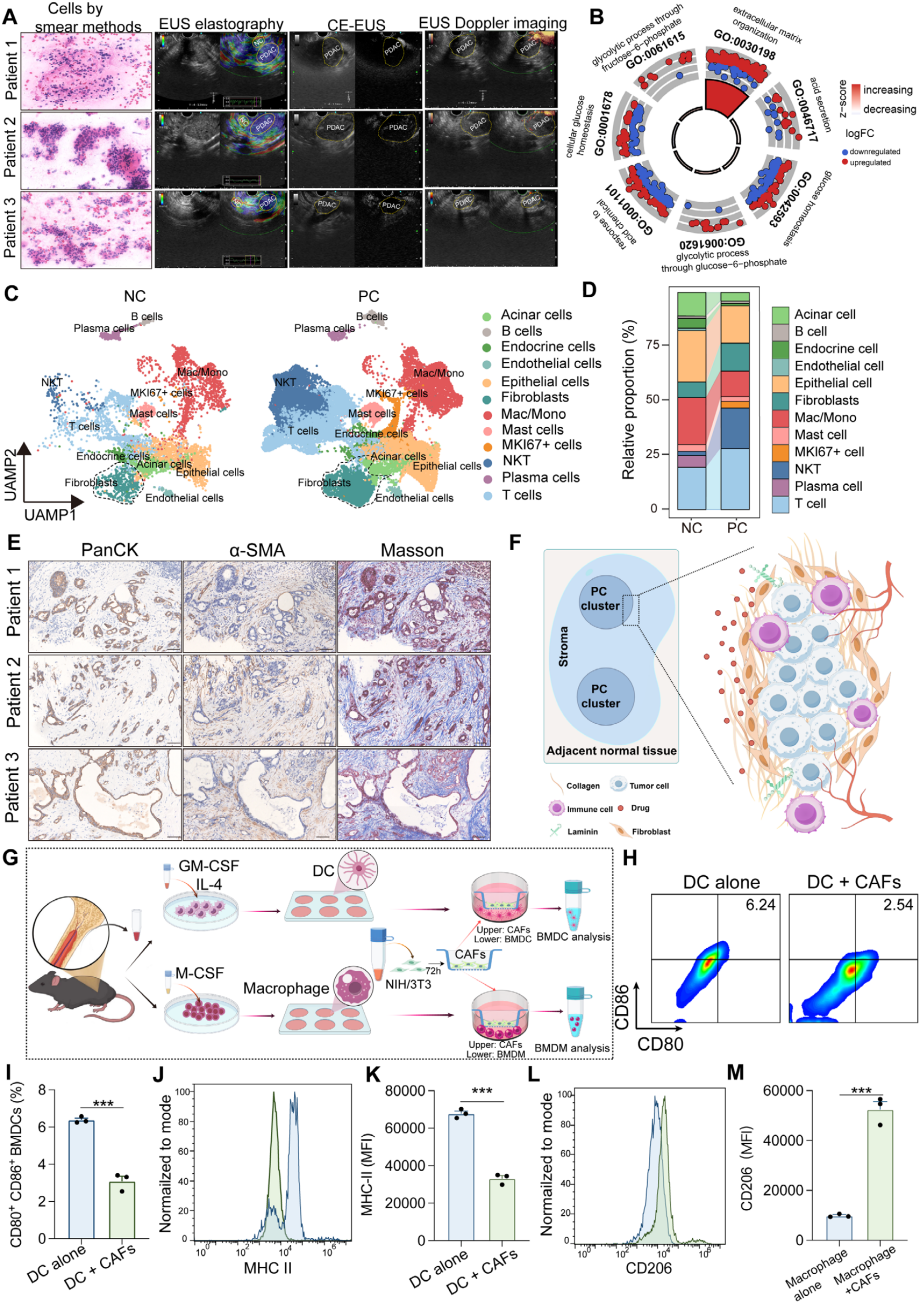
CAFs form a compact stromal barrier and modulate myeloid cell function. (A) Representative endoscopic ultrasound (EUS) images of pancreatic cancer (PC) lesions, including ROSE cytology from fine‐needle aspiration, elastography, CE‐EUS, and Doppler ultrasound. (B) Gene set enrichment analysis of bulk RNA‐seq data showing ECM‐ and CAF‐associated gene signatures in PC. (C) UMAP visualization of single‐cell RNA‐seq profiles from adjacent non‐cancerous tissue (NC) and pancreatic cancer (PC) samples, showing 12 major cell populations. (D) Relative proportions of each cluster, highlighting increased fibroblasts (CAFs) in PC compared with NC. (E) Masson's trichrome staining and IHC for α‐SMA and PanCK showing desmoplastic stroma in human PC. Scale bar = 100 µm. (F) Schematic illustration of CAF‐mediated stromal barriers. (G) Schematic illustration of CAF co‐culture with BMDCs and BMDMs. (H–K) Flow cytometry analysis of BMDCs after co‐culture with CAFs, showing reduced CD80/CD86 (H,I) and MHC II (J,K) expression (n = 3). (L,M) Flow cytometry analysis of BMDMs after co‐culture with CAFs, showing increased CD206 expression. Data are presented as mean ± SD (n = 3). Statistical significance was determined by unpaired two‐tailed Student's t‐test. ^***^ *p* < 0.001.

Endoscopic ultrasound (EUS) is the most sensitive imaging modality for detecting pancreatic cancer (PC) and is routinely used in clinical practice [32]. To leverage this diagnostic capability, we first assessed EUS findings in our patient cohort. Rapid on‐site evaluation (ROSE) of fine‐needle aspiration specimens confirmed the malignant epithelial nature of the lesions. Elastography revealed markedly increased tumor stiffness, whereas contrast‐enhanced EUS and Doppler imaging demonstrated significantly reduced intratumoral perfusion, together indicating a highly fibrotic and poorly vascularized tumor microenvironment (Figure 1A; Figure S1). To investigate the molecular basis of this phenotype, bulk RNA‐seq analysis of primary PC versus adjacent tissues revealed enrichment of extracellular matrix (ECM)‐ and CAF‐related gene signatures (Figure 1B) [33]. Reanalysis of single‐cell RNA‐seq data from our published study [34] identified 12 major cell clusters, with fibroblasts (CAFs) emerging as the dominant stromal population in PC (Figure 1C,D). This observation was further supported by independent single‐cell data from Peng et al.[35], which also confirmed CAF predominance (Figure S2).

Histological staining of patient samples further validated these findings. α‐SMA⁺ fibroblasts were observed to encase PanCK⁺ tumor nests, while Masson's trichrome staining highlighted extensive collagen deposition. These fibroblast–collagen “wall‐like” structures fused as tumors expanded, forming continuous stromal barriers that not only impeded drug penetration but also restricted immune cell infiltration (Figure 1E,F).

To further validate the protumorigenic role of CAFs, NIH/3T3 fibroblasts stimulated with PC cell–conditioned medium exhibited marked α‐SMA induction (Figure S3) [36], and co‐inoculation of CAFs with PC cells significantly accelerated subcutaneous tumor growth in vivo (Figure S4A–C). Beyond promoting tumor progression, CAFs also contributed to immune suppression: in co‐culture assays, they impaired dendritic cell maturation, as indicated by reduced MHC II and CD80/CD86 expression (Figure 1H–K), and drove macrophage polarization toward an M2‐like phenotype, characterized by elevated CD206 expression (Figure 1L,M).

Together, these findings demonstrate that CAFs construct dense stromal barriers and suppress antitumor immunity via myeloid modulation, underscoring stromal remodeling as a critical strategy to overcome both physical and immunological resistance in PC.

### 2.2 Construction and Characterization of ICyM2 with Enzyme‐Responsive Properties

We first designed ICyOH, a di‐iodinated heptamethine cyanine derivative, to enhance reactive oxygen species (ROS) generation and enable mitochondrial targeting. Heavy‐atom substitution was employed to accelerate intersystem crossing and increase ROS yield while retaining photothermal properties. The chemical structure of ICyOH was confirmed by ^1^H NMR, ^13^C NMR, and high‐resolution mass spectrometry (HR‐MS) (Figure S5A–C).

**FIGURE 2:**
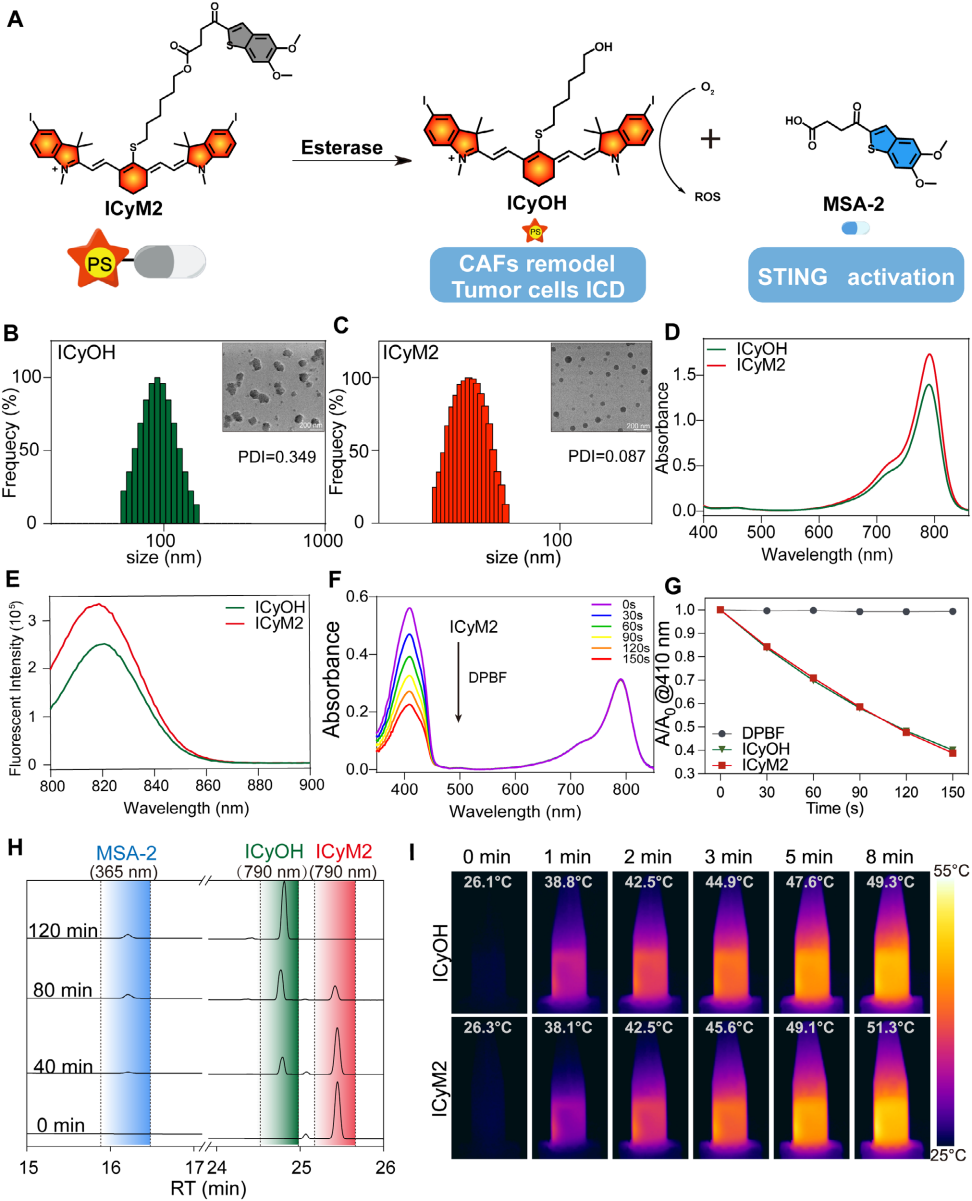
Construction and characterization of ICyM2 with enzyme‐responsive properties. (A) Schematic illustration of ICyM2 design, generated by covalently linking the photosensitizer ICyOH with the STING agonist MSA‐2 via an esterase‐cleavable ester bond. (B,C) DLS and TEM analyses showed that ICyM2 formed uniform spherical nanoaggregates, whereas ICyOH aggregated into larger, less uniform particles. (D,E) Absorption and fluorescence spectra of ICyOH and ICyM2, both displaying nearly identical optical properties. (F,G) Comparable singlet‐oxygen quantum yields confirming preserved photosensitizing capacity after conjugation. (H) Esterase responsiveness of ICyM2, with progressive cleavage into ICyOH and MSA‐2 upon incubation with esterase. (I) Photothermal heating curves under 808 nm laser irradiation, demonstrating robust photothermal effects of both ICyOH and ICyM2.

Guided by a photoimmunotherapy rationale, we constructed ICyM2 by covalently linking ICyOH to the STING agonist MSA‐2 via an esterase‐cleavable ester (Figure 2A), and verified its structure by ^1^H/^13^C NMR and HR‐MS (Figure S6A–C). This design enables esterase‐catalyzed hydrolysis within the tumor microenvironment, releasing both components in situ to potentiate efficacy while reducing systemic exposure.

In aqueous media, ICyM2 spontaneously formed uniform spherical nanoaggregates (hydrodynamic diameter 66.9 ± 2.3 nm; PDI 0.087), whereas ICyOH formed larger, less uniform aggregates (92.2 ± 6.2 nm; PDI 0.349) as determined by DLS and TEM (Figure 2B,C). ICyM2 maintained a nearly constant hydrodynamic diameter over 7 days in PBS with 10% FBS (Figure S7). Moreover, ICyM2 exhibited a moderately negative zeta potential (∼ −24 mV), in contrast to the slightly positive charge of ICyOH (∼ +5 mV) (Figure S8). Photophysical measurements showed nearly overlapping absorption (∼790 nm) and emission (∼820 nm) spectra for ICyOH and ICyM2 (Figure 2D,E; Figure S9A–E), with comparable singlet‐oxygen quantum yields (ΦΔ: ICyOH 0.32; ICyM2 0.28), indicating that conjugation preserved photosensitizing capacity (Figure 2F,G). Esterase responsiveness was validated by incubating ICyM2 with porcine liver esterase at 37 °C, detecting released ICyOH and MSA‐2 by ∼40 min and achieving near‐complete cleavage by ∼2 h (Figure 2H); HR‐MS further confirmed the released ICyOH (m/z 833.1467; Figure S10A–D). Under 808 nm irradiation (0.5 W cm^−^^2^), both ICyOH and ICyM2 exhibited robust photothermal heating, reaching ∼49–51 °C (Figure 2I; Figures S11 and S12). Together, these data establish ICyM2 as an esterase‐activatable, carrier‐free photoimmunotherapy conjugate that retains photophysical/photothermal features and enables on‐site MSA‐2 release.

### 2.3 ICyM2 Remodels CAFs and Induces Tumor ICD to Promote Myeloid Activation

**FIGURE 3:**
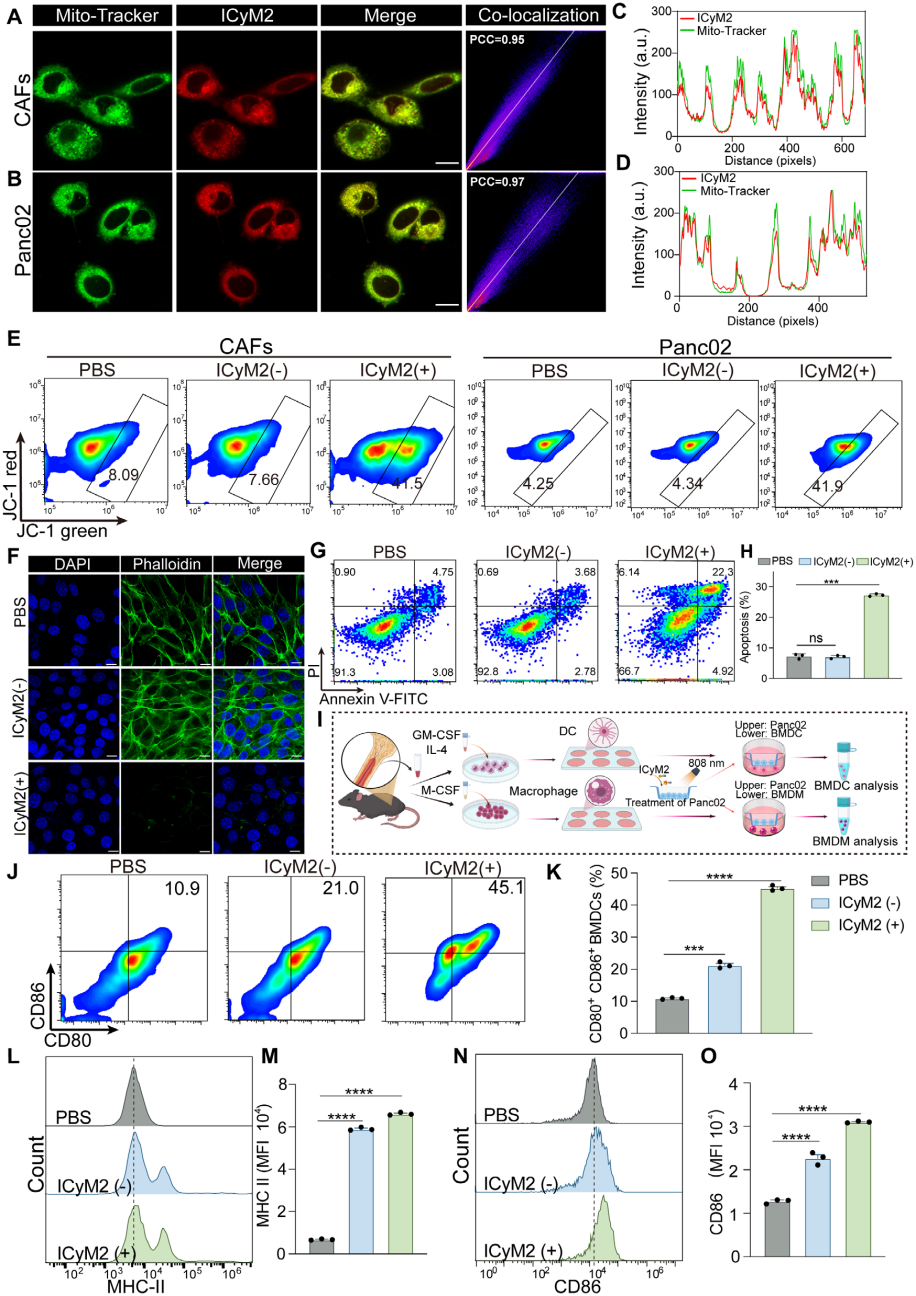
ICyM2 Remodels CAFs and Induces Tumor ICD to Promote Myeloid Activation. (A,B) Representative confocal images of CAFs (A) and Panc02 tumor cells (B) showing strong co‐localization of ICyM2 with mitochondria (MitoTracker). Scale bar = 25 µm. (C,D) Line‐scan intensity profiles confirming spatial overlap of ICyM2 and MitoTracker signals in CAFs (C) and Panc02 cells (D). (E) Flow cytometry analysis of mitochondrial membrane potential using JC‐1. (F) Phalloidin staining showing cytoskeletal disruption in CAFs. (G,H) Annexin V/PI flow cytometry and quantification of apoptosis in Panc02 cells. (I) Schematic illustration of the transwell co‐culture system with BMDCs or BMDMs and ICyM2‐treated Panc02 cells. (J,K) Flow cytometry analysis of CD80 and CD86 expression in BMDCs. (L,M) MHC‐II expression in BMDCs. (N,O) Flow cytometry analysis of CD86 expression in BMDMs. Data are presented as mean ± SD (n = 3). Statistical significance was determined by one‐way ANOVA followed by Tukey's post hoc test. ns, not significant; ^***^ *p* < 0.001; ^****^ *p* < 0.0001.

Immunofluorescence imaging demonstrated efficient ICyM2 uptake in both α‐SMA⁺ CAFs and PanCK⁺ Panc02 cells, as verified by line‐scan intensity analysis (Figure S13A–D). As a lipophilic cation, ICyM2 readily crossed cellular and mitochondrial membranes, showing strong co‐localization with MitoTracker (Pearson's coefficients: 0.95 in CAFs, 0.97 in Panc02; Figure 3A–D). We then assessed its photodynamic cytotoxicity by CCK‐8 assays. ICyM2 exhibited negligible toxicity in the dark, whereas NIR irradiation induced a clear dose‐dependent decrease in viability, reducing survival of both CAFs and Panc02 cells to <15% at 4 µM (Figure S14A, B). Consistent with its preserved photophysical properties (Figure 2G), ICyM2 displayed a singlet oxygen quantum yield of 0.28, and DCFH‐DA assays confirmed robust intracellular ROS production upon NIR irradiation (Figure S15A–C).

Excessive ROS triggered profound mitochondrial dysfunction [37]. JC‐1 assays revealed a marked increase in depolarized mitochondria after ICyM2 + NIR treatment (Figure 3E; Figure S16A, B), corroborated by fluorescence imaging of mitochondrial membrane potential collapse (Figure S17). TEM analysis further showed classical ultrastructural damage, including mitochondrial shrinkage, cristae disintegration, and outer‐membrane rupture, specifically in the ICyM2 + NIR group but not in PBS controls (Figure S18). These mitochondrial injuries produced distinct outcomes in the two major cell types: CAFs exhibited cytoskeletal disruption as indicated by phalloidin staining (Figure 3F), whereas Panc02 tumor cells underwent significant apoptosis (Figure 3G,H) accompanied by hallmarks of immunogenic cell death (ICD), including ecto‐CRT exposure, HMGB1 release, ATP secretion, and γ‐H2AX foci formation (Figures S19 and S20). Together, these results demonstrate that ICyM2 disrupts CAF structure while inducing ICD in tumor cells, thereby priming a more immune‐permissive microenvironment.

To further evaluate downstream immune effects, we examined whether ICyM2 could modulate myeloid cell function (Figure 3I). Western blotting revealed pronounced phosphorylation of STING, TBK1, and IRF3, and ELISA demonstrated significantly elevated IFN‐β and CXCL10 secretion, together confirming robust activation of the cGAS–STING pathway in APCs (Figures S21 and S22). Flow cytometry revealed that ICyM2 + NIR substantially enhanced dendritic cell maturation: CD80/CD86⁺ DCs increased ∼4‐fold compared with PBS and ∼2‐fold relative to ICyM2(–) (Figure 3J,K), while MHC‐II expression rose nearly 10‐fold versus PBS (Figure 3L,M). In macrophages, ICyM2 + NIR treatment markedly shifted polarization toward the M1 phenotype, as evidenced by a ∼2.5‐fold increase in CD86 expression relative to PBS and a ∼1.3‐fold increase relative to ICyM2(–) (Figure 3N,O). Correspondingly, the proportion of CD206⁺ M2 macrophages was reduced by ∼83% compared with PBS and ∼63% compared with ICyM2(–) (Figure S23).

Collectively, these findings demonstrate that ICyM2 not only remodels CAFs and elicits tumor ICD but also synergistically activates the STING pathway to enhance DC maturation and repolarize macrophages. This dual action fosters a potent myeloid‐driven antitumor immune response, establishing a strong mechanistic rationale for ICyM2‐based photoimmunotherapy in PC.

### 2.4 ICyM2 Exhibits Preferential Tumor Accumulation and Enables Multimodal Imaging

**FIGURE 4:**
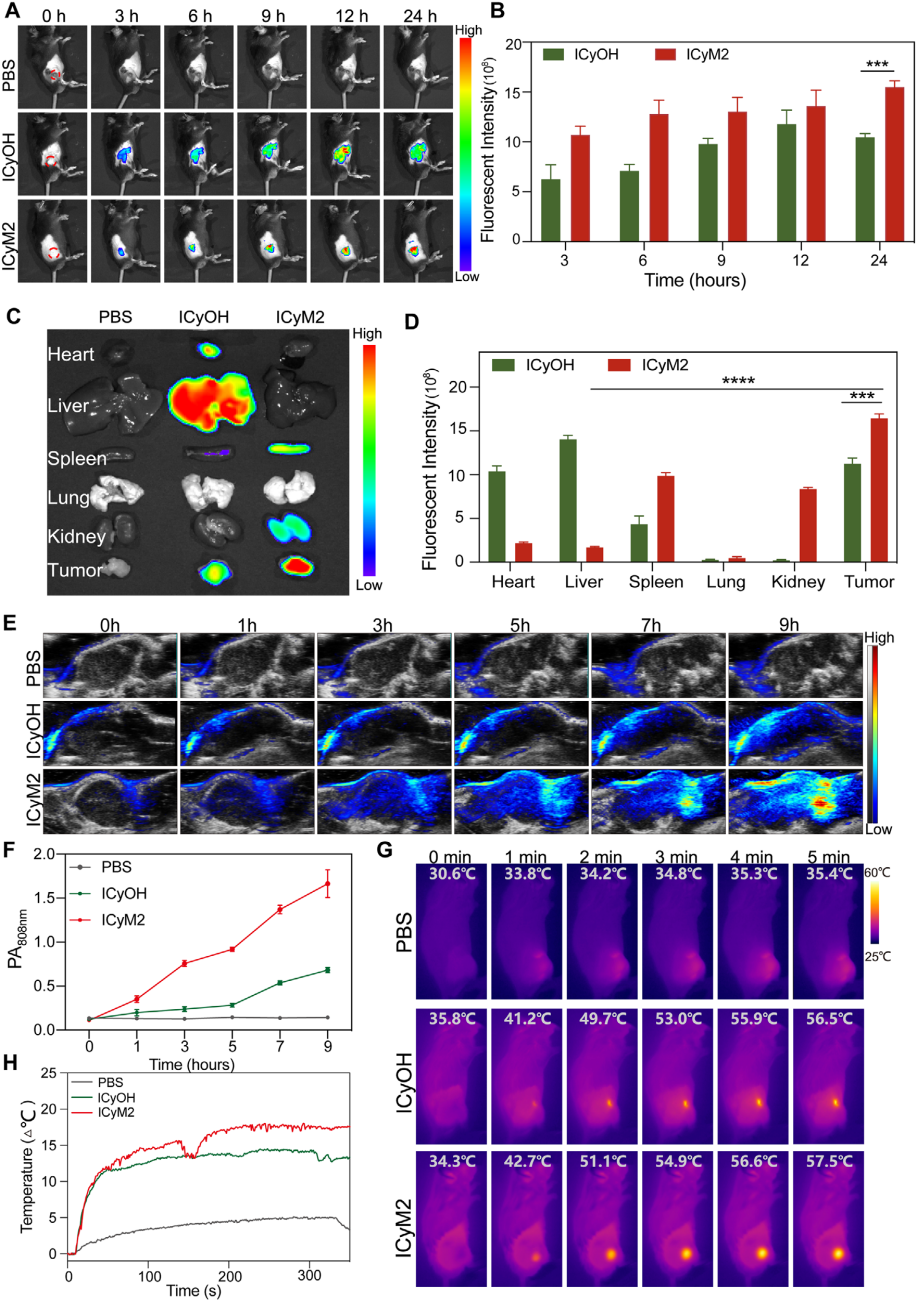
Enhanced Tumor Accumulation and Multimodal Imaging of ICyM2 (A) NIR fluorescence images of tumor‐bearing mice at different time points after intravenous injection of PBS, ICyOH, or ICyM2. (B) Quantitative analysis of tumor fluorescence intensity over time. (C) Ex vivo NIR fluorescence images of major organs and tumors at 24 h post‐injection. (D) Quantitative analysis of fluorescence intensity in major organs and tumors. (E) Photoacoustic (PA) images (808 nm) of tumor‐bearing mice at different time points after injection of ICyOH or ICyM2. (F) Quantitative analysis of PA signal intensity in the tumor region over time. G) Infrared thermal images of tumor‐bearing mice from different groups under 808 nm laser irradiation. (H) Tumor temperature profiles during laser irradiation. Data are presented as mean ± SD (n = 3). Statistical significance was determined by unpaired two‐tailed Student's t‐test. ^***^ *p* < 0.001; ^****^ *p* < 0.0001.

Encouraged by its favorable in vitro photophysical and photothermal properties, we next investigated the in vivo biodistribution and imaging performance of ICyM2. A desmoplastic tumor model was established by co‐inoculating Panc02 cells and CAFs into mice. Near‐infrared (NIR) fluorescence imaging revealed rapid and selective tumor accumulation of ICyM2, with fluorescence intensity at 24 h exceeding background by more than 1000‐fold, whereas ICyOH peaked earlier (12 h, ∼770‐fold increase) and declined thereafter, indicating inferior retention. These results highlight ICyM2's superior tumor targeting and prolonged intratumoral retention, which can be attributed to its optimized size (∼64 nm) and surface charge (∼−24 mV), enhancing EPR‐mediated tumor accumulation and minimizing nonspecific uptake, as previously reported [38, 39](Figure 4A,B).

Ex vivo fluorescence imaging at 24 h confirmed these findings (Figure 4C). ICyM2 produced strong tumor signals with only moderate spleen and kidney uptake, while ICyOH accumulated prominently in the liver. Quantitative analysis further demonstrated a tumor‐to‐liver ratio of ∼9.5 for ICyM2, ∼12‐fold higher than ICyOH (∼0.8), highlighting the benefit of its optimized nanoaggregate size and uniform morphology (Figure 4D).

Beyond fluorescence, ICyM2 enabled robust photoacoustic (PA) imaging for real‐time tumor monitoring. PA signals at 808 nm increased steadily, peaking at 9 h post‐injection, with ICyM2 consistently outperforming ICyOH across all time points (Figure 4E,F). In parallel, in vivo photothermal performance was assessed. Upon 808 nm laser irradiation, tumors in ICyM2‐ and ICyOH‐treated mice rapidly heated to 57.5 and 56.5 °C, respectively, whereas PBS controls rose only to 35.4 °C (Figure 4G,H), confirming that ICyM2 maintains strong photothermal properties.

Collectively, these results demonstrate that ICyM2 achieves preferential tumor accumulation, reduced off‐target hepatic uptake, and superior multimodal imaging capabilities, providing a robust foundation for subsequent therapeutic evaluation.

### 2.5 The Anti‐Tumor Efficacy of ICyM2

**FIGURE 5:**
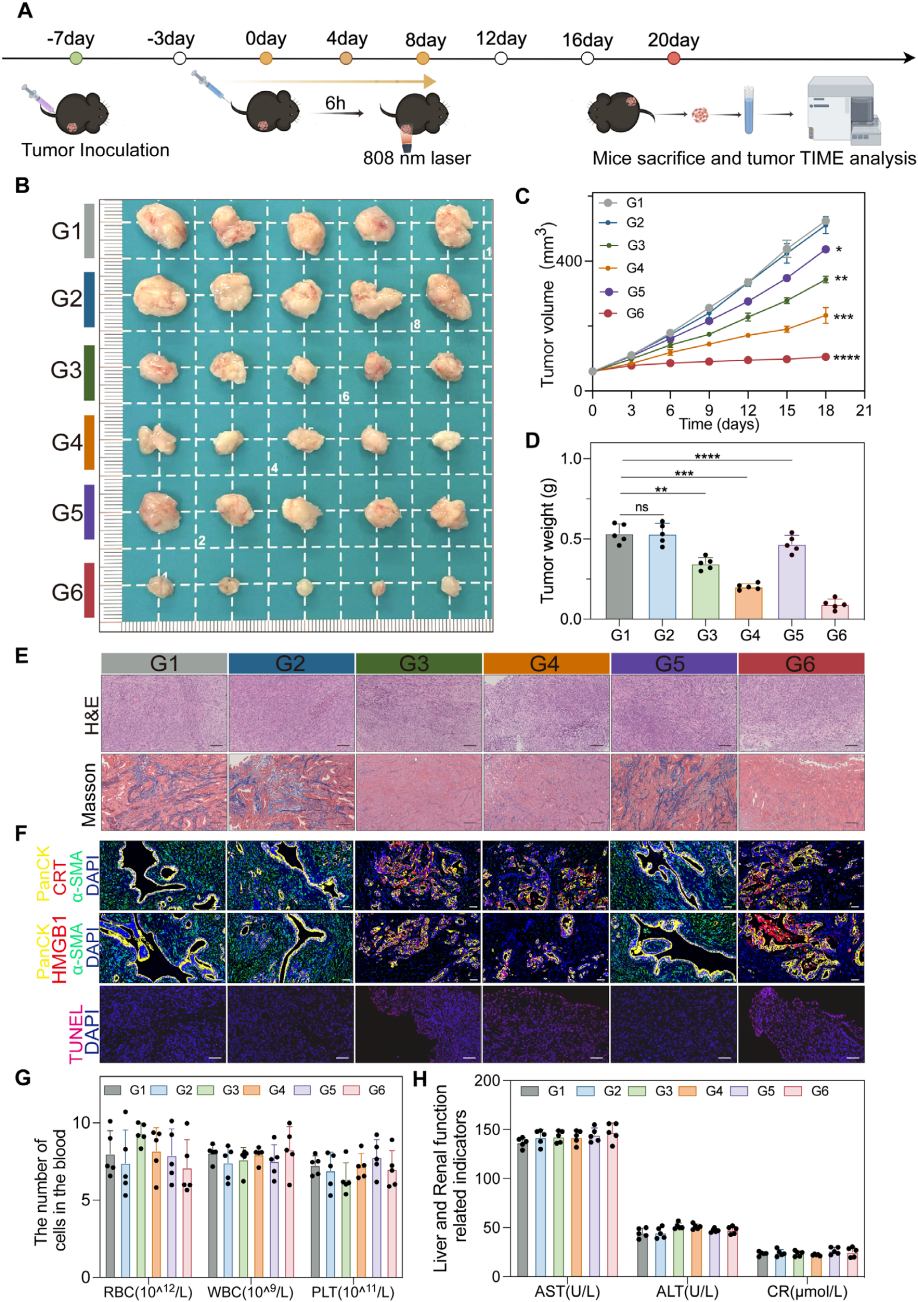
In vivo antitumor effects of ICyM2. (A) Schematic illustration of the treatment regimen. (B) Representative tumor photographs from each group. (C) Tumor growth curves. (D) Tumor weights of different treatment groups (n = 5). (E) H&E and Masson's trichrome staining of tumor sections at the endpoint. Scale bar = 100 µm. (F) Multiplex immunofluorescence and TUNEL staining of tumor sections at the endpoint. Blue: DAPI; Green: α‐SMA; Yellow: PanCK; Red: CRT or HMGB1; TUNEL shown separately. Scale bar = 50 µm. (G,H) Hematological parameters (G) and liver/kidney function markers (H) at the end of the study. Definitions of treatment groups (G1–G6): PBS (G1), MSA‐2 (G2), ICyOH + Laser (G3), ICyOH + MSA‐2 + Laser (G4), ICyM2 without irradiation (G5), and ICyM2 + Laser (G6). Data are presented as mean ± SD (n = 5). Statistical significance was determined by one‐way ANOVA followed by Tukey's post hoc test. ^*^ *p* < 0.05, ^**^ *p* < 0.01, ^***^ *p* < 0.001; ^****^< 0.0001.

To recapitulate the pancreatic tumor microenvironment, a desmoplastic subcutaneous model was generated by co‐inoculating Panc02 cells with CAFs. Tumor‐bearing mice were randomized into six groups (G1–G6): PBS (G1), MSA‐2 (G2), ICyOH + Laser (G3), ICyOH + MSA‐2 + Laser (G4), ICyM2 without irradiation (G5), and ICyM2 + Laser (G6). MSA‐2 was administered intravenously in G2 and G4, while designated groups received 808 nm irradiation (0.5 W cm^−^^2^, 5 min) 6 h post‐injection (Figure 5A). Upon systemic administration, ICyM2 preferentially accumulated in tumors and underwent esterase‐mediated cleavage to release ICyOH and MSA‐2 in situ. Within the PC microenvironment, tumor cells and CAFs constitute the predominant compartments [40]. ICyOH generated ROS under NIR irradiation, disrupting the fibrotic stroma and facilitating deeper intratumoral penetration of MSA‐2. This sequential action promoted DC maturation, macrophage repolarization, and amplification of ICD signaling, thereby overcoming CAF‐driven immunosuppression.

Tumor photographs (Figure 5B) and growth curves (Figure 5C) demonstrated negligible efficacy of MSA‐2 monotherapy (G2), moderate inhibition with ICyOH + Laser (G3), and progressively stronger suppression in ICyOH + MSA‐2 + Laser (G4) and ICyM2 groups (G5–G6). Strikingly, ICyM2 + Laser (G6) achieved the most pronounced effect, reducing tumor weights on day 20 by ∼12.0−, 11.4−, 6.8−, 6.5−, and 3.8‐fold compared with PBS, MSA‐2, ICyOH + Laser, ICyOH + MSA‐2 + Laser, and ICyM2 (–), respectively (Figure 5D).

Histological analyses corroborated these findings. Tumors from G6 mice exhibited marked stromal remodeling, including reduced CAF abundance and collagen rearrangement (Figure 5E; Figure S24), accompanied by extensive tumor necrosis, strong CRT exposure, HMGB1 release, increased apoptosis, and reduced proliferation (Figure 5F). Serum IL‐6 levels, measured 6 h post‐treatment as an early indicator of systemic inflammation, remained near baseline in the ICyM2‐treated groups (G5, G6), significantly lower than those in the free MSA‐2 group (G2) (Figure S25), indicating that ICyM2 significantly reduces the systemic inflammatory response typically associated with MSA‐2. While a transient increase in IL‐6 was observed early on, all treatments were well tolerated, with stable body weight, normal hematological and liver/kidney function indices, and no observable pathological abnormalities in major organs (Figure 5G,H; Figure S26A,B). These results demonstrate that ICyM2 is safe both in the short‐term and long‐term, without causing significant systemic inflammation or toxicity.

Collectively, these results demonstrate that ICyM2 + Laser achieves potent antitumor efficacy by sequentially disrupting stromal barriers, inducing ICD, and enhancing MSA‐2 delivery, thereby establishing a strong rationale to further investigate how ICyM2 reshapes the tumor immune microenvironment.

### 2.6 In Vivo Mechanistic Study of ICyM2‐Mediated Antitumor Immunity

Having established the potent antitumor efficacy of ICyM2 in vivo, we next investigated the underlying immune mechanisms. Immunofluorescence staining revealed robust STING pathway activation in tumors from the ICyM2 + Laser (G6) group, with p‐TBK1 and p‐IRF3 levels elevated by ∼16.8− and ∼12.5‐fold compared with PBS controls (G1). By contrast, ICyOH + MSA‐2 + Laser (G4) and ICyOH + Laser (G3) induced only ∼3.9− and ∼3.3‐fold increases, respectively (Figure S27A,B). Consistently, serum IFN‐β concentrations in G6 were ∼15.9‐fold higher than PBS, compared with ∼3.9− and ∼2.9‐fold increases in G4 and G3 (Figure S28). The moderate activation in G3 reflects ICD signals from irradiated ICyOH, while the partial increase in G4 likely derives from ICD priming combined with limited intratumoral entry of systemically administered MSA‐2 after stromal remodeling. In contrast, ICyM2 provided tumor‐localized, esterase‐activated co‐delivery of ICyOH and MSA‐2, achieving spatiotemporal overlap with ICD signals and eliciting far stronger STING pathway activation.

**FIGURE 6:**
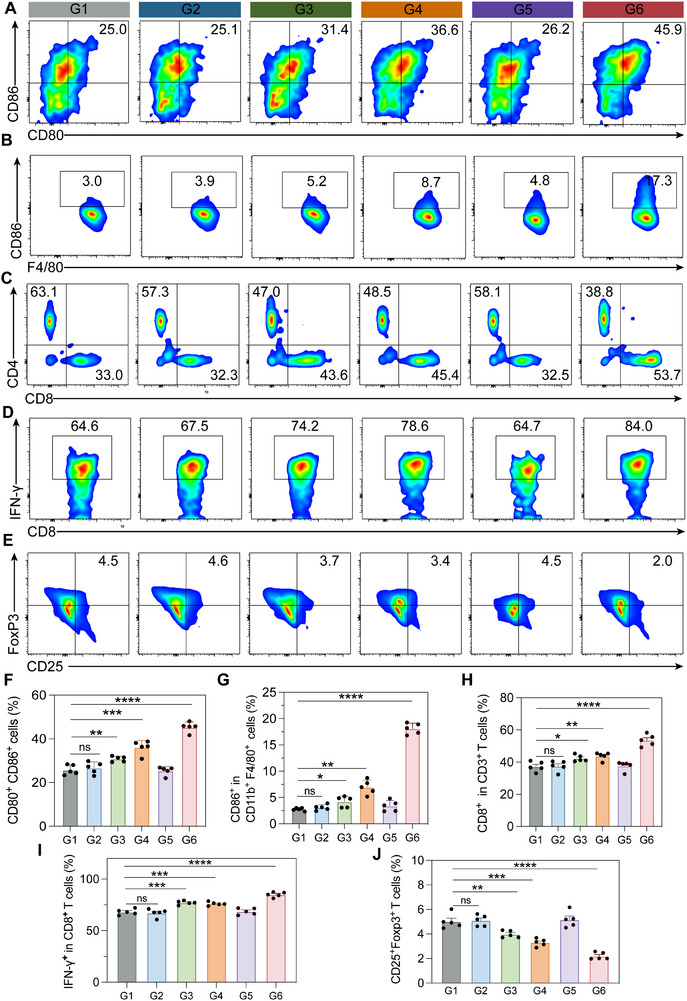
ICyM2 reshapes the tumor immune microenvironment. (A–E) Flow cytometry analysis of dendritic cell (DC) maturation (A), M1‐like macrophages (B), CD8⁺ and CD4⁺ T cells (C), IFN‐γ⁺ CD8⁺ T cells (D), and regulatory T cells (Tregs) (E) in tumors. (F–J) Quantification of DC maturation (F), M1‐like macrophages (G), CD8⁺ T cells (H), IFN‐γ⁺ CD8⁺ T cells (I), and Tregs (J). Data are presented as mean ± SD (n = 5). Statistical significance was determined by one‐way ANOVA followed by Tukey's post hoc test. ns, not significant; ^*^ *p* < 0.05; ^**^ *p* < 0.01; ^***^ *p* < 0.001; ^****^ *p* < 0.0001.

To further dissect immune mechanisms, key myeloid populations were profiled (Figure S29). Photosensitizer monotherapy (G3) modestly enhanced DC maturation (∼1.2‐fold vs PBS), whereas ICyM2 without irradiation (G5) showed minimal effect, consistent with insufficient intratumoral activation. By contrast, ICyM2 + Laser (G6) induced the strongest response, increasing DC maturation by ∼1.7‐fold relative to PBS (Figure 6A–F), reflecting ICD‐driven antigen release combined with localized MSA‐2 delivery. Tumor‐associated macrophages (TAMs) were also reprogrammed: M2‐like macrophages decreased by ∼2.9‐fold, while pro‐inflammatory M1‐like macrophages increased by ∼5.8‐fold versus PBS (Figure S30A,B). These changes underscore ICyM2's ability to disrupt stromal barriers and rewire immunosuppressive myeloid niches, creating a permissive context for T cell priming and infiltration.

Analysis of lymphocyte subsets further demonstrated broad reshaping of adaptive and innate immunity (Figure S31). ICyM2 + Laser (G6) tumors exhibited the highest CD8⁺ T cell infiltration, accompanied by a ∼1.5–1.8‐fold increase in the CD8/CD4 ratio compared with all other groups (Figure 6C,H). Functionally, CD8⁺ T cells in G6 displayed ∼1.2–1.3‐fold higher IFN‐γ production than PBS controls (Figure 6D,I). In parallel, Tregs were reduced by ∼2–3‐fold (Figure 6E,J; Figure S32), NK cell infiltration was markedly enhanced (∼2.5–3.5‐fold vs PBS) (Figure S33A–C), and MDSCs were reduced by ∼2‐fold (Figure S34A–C).

Collectively, these findings demonstrate that ICyM2‐mediated sequential stromal clearance and immune stimulation not only activate innate immune sensors (STING) but also reprogram myeloid compartments, expand cytotoxic T and NK cell populations, and suppress inhibitory subsets. These coordinated changes effectively convert immunologically “cold” pancreatic tumors into “hot” tumors.

### 2.7 ICyM2 Induces Immune Memory Against Tumor Metastasis

**FIGURE 7:**
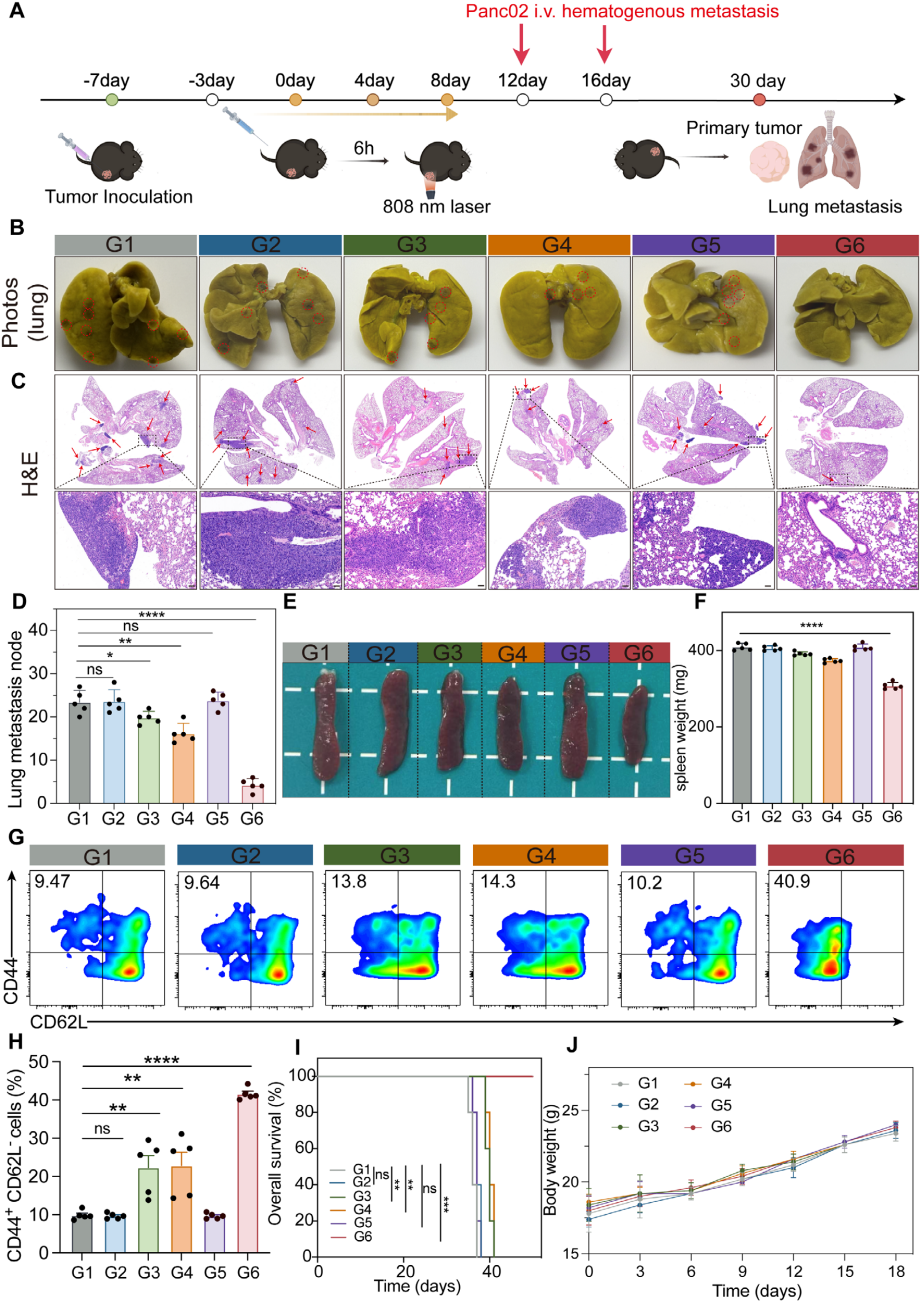
ICyM2 Induces Immune Memory against Tumor Metastasis (A) Schematic illustration of the treatment regimen. (B) Representative Bouin's‐stained lung images. (C) H&E staining of lung sections from different treatment groups. (D) Quantification of metastatic lung nodules. (E,F) Representative spleen images (E) and spleen weights (F). (G, H) Flow cytometry analysis (G) and quantification (H) of effector memory T (TEM) cells in spleens after treatment. (I) Survival curves of tumor‐bearing mice after different treatments. (J) Body weight changes during treatment. Data are presented as mean ± SD (n = 5). Statistical significance was determined by one‐way ANOVA followed by Tukey's post hoc test. ns, not significant; ^*^ *p* < 0.05; ^**^ *p* < 0.01; ^****^ *p* < 0.0001.

Mature dendritic cells (DCs), cytotoxic CD8⁺ T cells, and M1‐like tumor‐associated macrophages (TAMs) cooperate to establish systemic antitumor immunity and suppress metastasis [41]. To evaluate whether ICyM2 could prevent tumor dissemination, we employed a hematogenous metastasis model (Figure 7A). After primary tumor treatment, Panc02 cells were intravenously injected on days 12 and 16 to mimic aggressive systemic spread. Compared with spontaneous metastasis, this approach yields a higher metastatic burden and provides a stringent test of anti‐metastatic efficacy.

Consistent with earlier findings, ICyM2 + Laser (G6) markedly inhibited primary tumor growth (Figure S35A–C). Strikingly, lung metastasis was almost completely blocked: G6‐treated mice displayed no visible metastatic nodules, whereas numerous lesions were present in all control groups (Figure 7B). H&E staining confirmed extensive metastatic foci in control lungs, while lungs from G6‐treated mice were nearly free of metastases (Figure 7C). Quantitative analysis showed a ∼5.6‐fold reduction in lung metastases compared with PBS (G1) (Figure 7D), highlighting the exceptional anti‐metastatic efficacy of ICyM2 + Laser.

Systemic disease burden was reflected by splenomegaly in controls, whereas spleens from G6‐treated mice remained near normal size, suggesting efficient clearance of circulating tumor cells. To assess long‐term immune memory, splenic effector memory T cells (TEMs, CD62L^−^CD44⁺) were profiled (Figure S36). G6 treatment induced a ∼4.4‐fold expansion of TEMs compared with PBS, indicating durable immune memory capable of preventing metastatic relapse. Consistently, ICyM2 + Laser prolonged overall survival, with all mice surviving to day 50, while all control mice succumbed by day 41 (Figure 7I). Body weight remained stable across groups, confirming the absence of overt systemic toxicity (Figure 7J).

Together, these findings demonstrate that ICyM2 + Laser not only eradicates established primary tumors but also prevents metastatic dissemination and induces robust, long‐term immune memory, thereby conferring durable survival benefits without detectable toxicity.

### 2.8 ICyM2 Synergizes with PD‐1 Blockade to Enhance Antitumor Immunity

**FIGURE 8:**
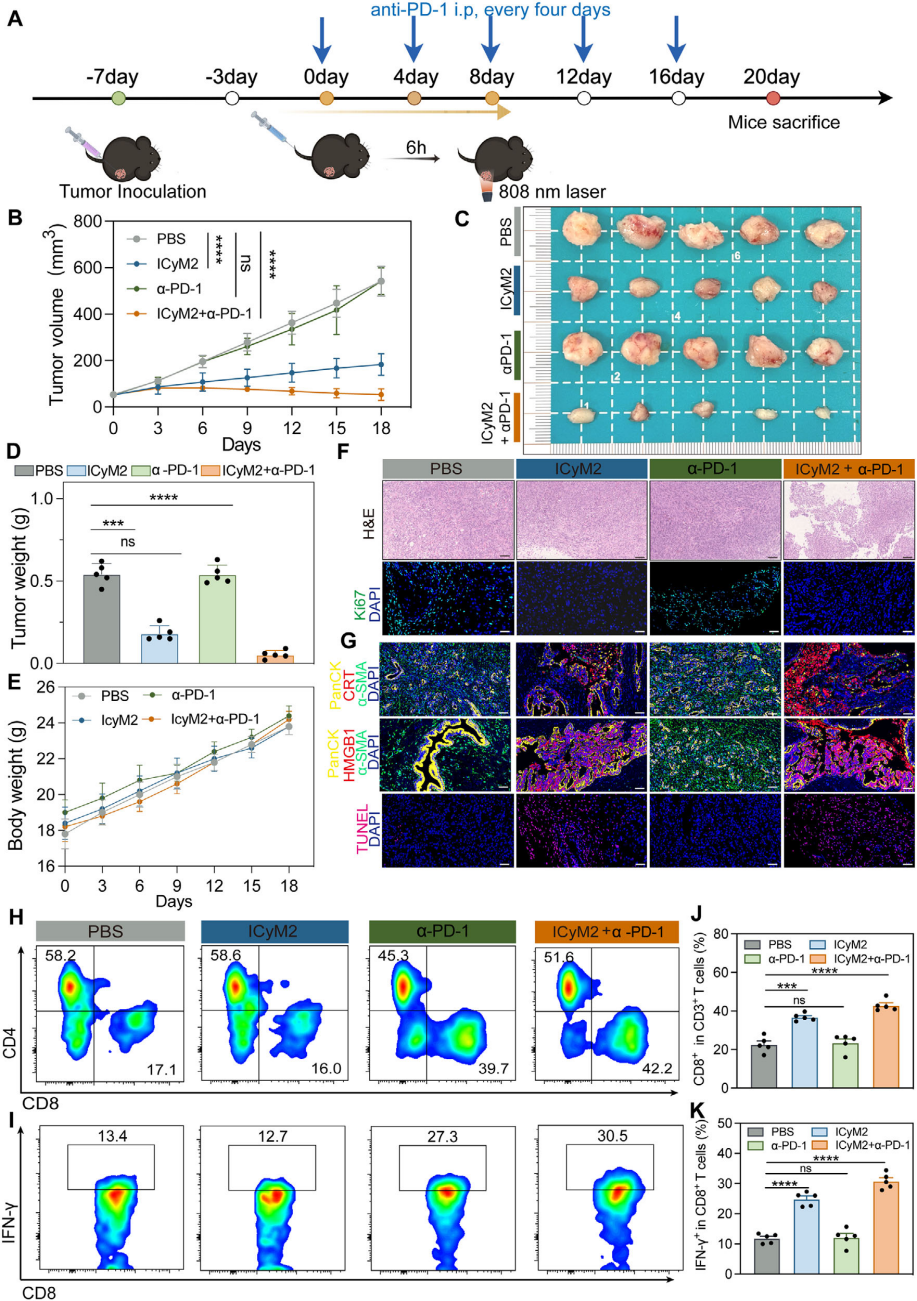
Synergistic effect of ICyM2 enhances the efficacy of anti‐PD‐1 antibody therapy (A) Schematic illustration of the treatment schedule. (B) Tumor growth curves (n = 5). (C) Representative images of excised tumors from each group. (D) Tumor weights of different treatment groups (n = 5). (E) Changes in body weight during treatment. (F) H&E and Ki67 staining of tumor sections at the endpoint. Scale bar = 100 µm. (G) Multiplex immunofluorescence and TUNEL staining of tumor sections at the endpoint. Blue: DAPI; Green: α‐SMA; Yellow: PanCK; Red: CRT or HMGB1; TUNEL shown separately. Scale bar = 50 µm. (H,I) Representative flow cytometry plots gated on CD3⁺ T cells (H: CD8⁺ T cells; I: IFN‐γ⁺ CD8⁺ T cells). (J, K) Quantification of CD8⁺ T cells (J) and IFN‐γ⁺ CD8⁺ T cells (K) among CD3⁺ T cells in tumors (n = 5). Data are presented as mean ± SD (n = 5). Statistical significance was determined by one‐way ANOVA followed by Tukey's post hoc test.ns, not significant;^***^ *p* < 0.001; ^****^ *p* < 0.0001.

Pancreatic cancer represents an immunologically “cold” tumor type with limited responsiveness to checkpoint inhibition [42]. To determine whether ICyM2 could potentiate anti–PD‐1 therapy, desmoplastic Panc02 tumors were established and treated with ICyM2 phototherapy in combination with anti–PD‐1 antibody every 4 days (Figure 8A). Tumor progression was monitored for 18 days. In the PBS group, tumors grew rapidly regardless of PD‐1 administration, and anti–PD‐1 monotherapy conferred only minimal benefit. ICyM2 monotherapy significantly inhibited tumor growth (∼4–5‐fold reduction vs PBS), while the combination regimen further reduced tumor burden by ∼5–6‐fold relative to ICyM2 alone (Figure 8B–D). Importantly, this synergy was achieved without systemic toxicity: body weights remained stable across groups (Figure 8E), hematological and renal indices were within normal limits, and histopathological analysis of major organs revealed no abnormalities (Figure S37A–C).

Histopathological and immunofluorescence analyses corroborated the superior efficacy of the combination treatment. H&E staining demonstrated extensive tumor necrosis, while Ki67 and TUNEL assays revealed decreased proliferation and increased apoptosis. Multicolor immunofluorescence further showed pronounced stromal remodeling, characterized by depletion of α‐SMA⁺ CAFs, together with robust ecto‐CRT exposure and HMGB1 release, hallmarks of ICD (Figure 8F,G).

Flow cytometry (gating in Figure S38) revealed comprehensive immune reprogramming. ICyM2 monotherapy increased CD8⁺ T cell infiltration (∼1.5‐fold vs PBS). When combined with PD‐1 blockade, CD8⁺ T cell levels rose further (∼1.2‐fold vs ICyM2 alone), while IFN‐γ⁺ CD8⁺ T cells expanded by ∼2–2.5‐fold compared with PBS, confirming enhanced cytotoxic function (Figures 8H–K).

Together, these findings establish ICyM2 as a potent immunomodulatory platform that synergizes with PD‐1 blockade to remodel stromal barriers, amplify ICD, and reinforce cytotoxic T cell responses. This strategy provides a promising avenue to sensitize otherwise refractory “cold” PC to checkpoint immunotherapy.

## 3 Conclusions

In this work, we developed ICyM2, a carrier‐free and esterase‐responsive nanoaggregate rationally constructed by covalently linking the mitochondria‐targeting photosensitizer ICyOH with the non‐nucleotide STING agonist MSA‐2. This single‐molecule design enables high drug loading, uniform nanoassembly, and selective tumor accumulation, thereby overcoming the delivery inefficiency and systemic toxicity associated with conventional carrier‐based formulations. Upon activation within the tumor microenvironment, ICyOH disrupts CAF‐mediated stromal barriers and induces immunogenic cell death (ICD), which facilitates the intratumoral penetration and effective activation of MSA‐2. The sequential release amplifies STING signaling, promotes dendritic cell maturation, reprograms macrophages toward an M1 phenotype, and enhances cytotoxic T‐cell infiltration.

As a result, ICyM2 achieves potent tumor regression, induces durable immune memory to prevent metastasis, and exhibits excellent systemic biosafety. Moreover, it synergizes with PD‐1 blockade to convert immunologically “cold” pancreatic tumors into “hot,” highlighting its translational potential. Collectively, this study establishes a rational design paradigm that integrates stromal remodeling and immune activation within a single carrier‐free nanoplatform, providing a promising strategy for next‐generation photoimmunotherapy in pancreatic cancer.

## Conflicts of Interest

The authors declare no conflict of interest.

## Supporting information




**Supporting File**: advs73463‐sup‐0001‐SuppMat.docx.

## Data Availability

The data that support the findings of this study are available from the corresponding author upon reasonable request.
