# Supplementary material for: STING Agonist‐Modified Tumor Targeting Photosensitizer Remodels Cancer‐Associated Fibroblasts to Potentiate Photoimmunotherapy in Pancreatic Cancer
Source: Adv Sci (Weinh). 2025 Dec 27;13(13):e20547. doi: 10.1002/advs.202520547 (PMC12955854; doi:10.1002/advs.202520547)
Supplement: Supplementary file 1 — Supporting File: advs73463‐sup‐0001‐SuppMat.docx. [file ADVS-13-e20547-s001.docx]

Supporting information

**STING agonist-Modified Tumor Targeting Photosensitizer Remodels Cancer-Associated Fibroblasts to Potentiate Photoimmunotherapy in Pancreatic Cancer**

**1. Supporting figures**


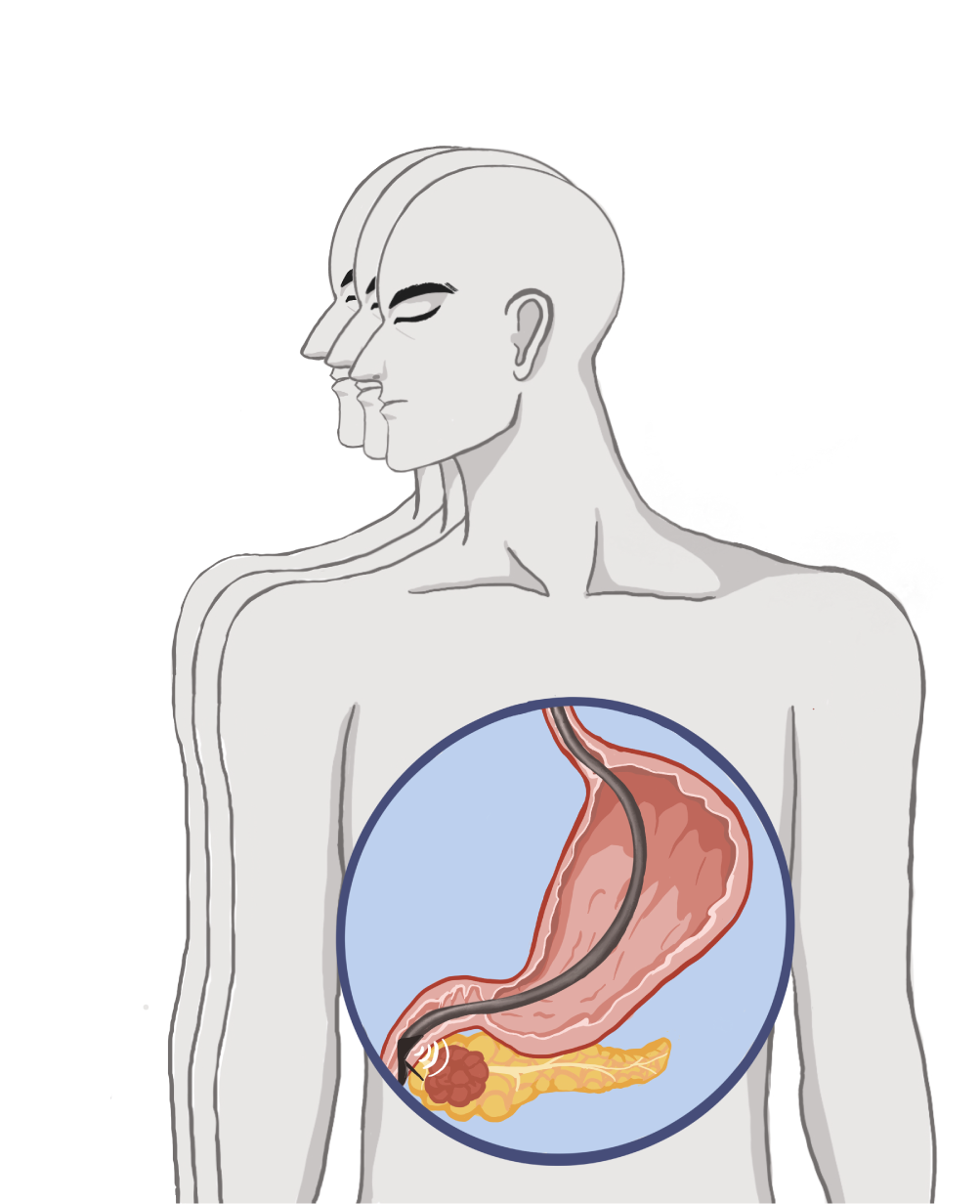


**Figure S1.** Schematic illustration of EUS examination.


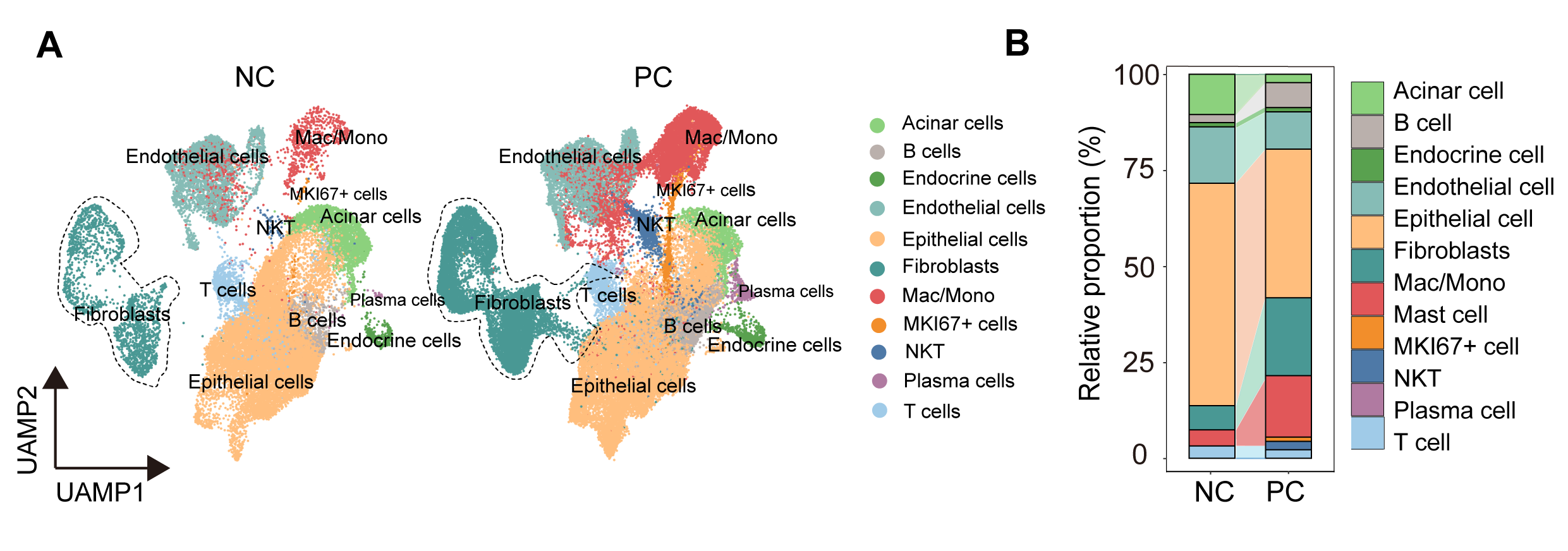


**Figure S2.** A) UMAP visualization of single-cell RNA-seq profiles from adjacent non-cancerous tissue (NC) and pancreatic cancer (PC) samples. B) Relative proportions of each cluster, highlighting increased fibroblasts (CAFs) in PC compared with NC.


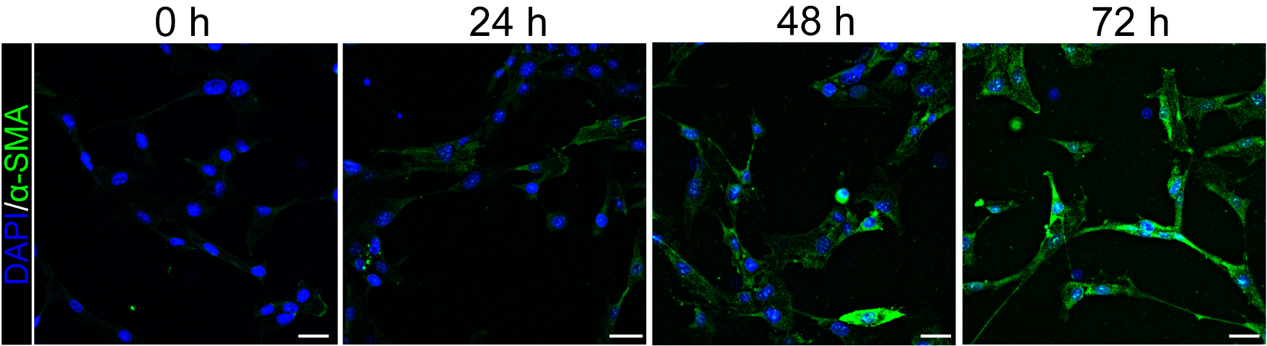


**Figure S3.** Immunofluorescence staining to detect the expression level of α-SMA in activated NIH/3T3 cells. Scale bar = 50 μm.


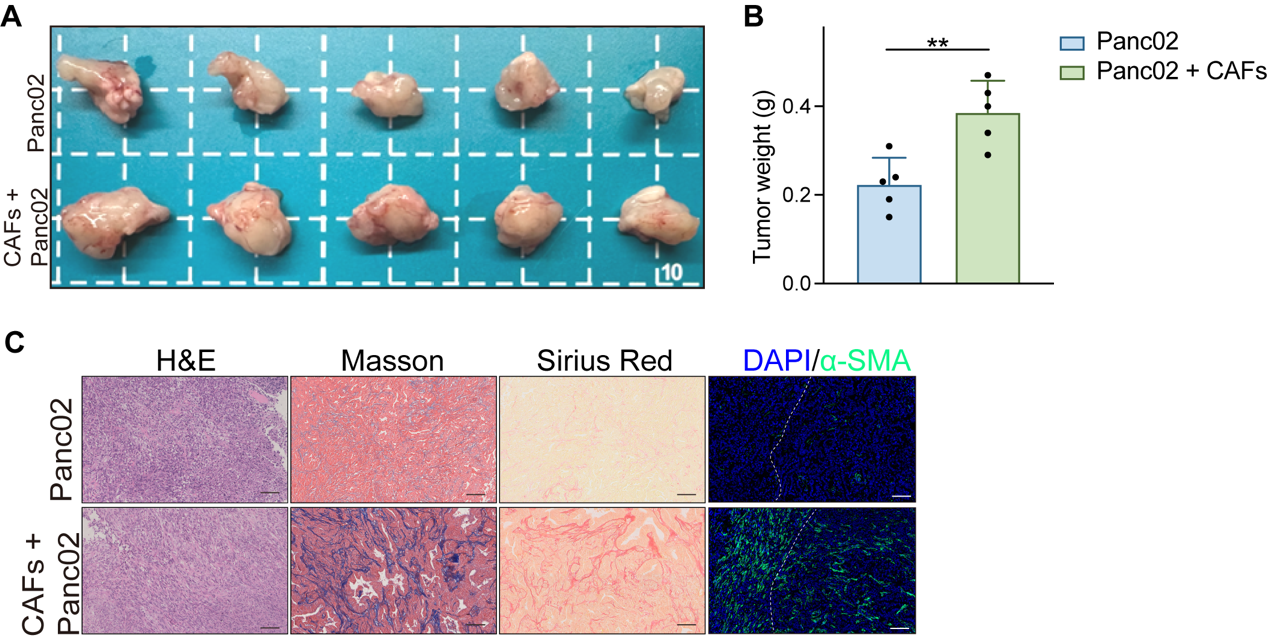


**Figure S4.** A) Representative tumor photographs. B) Tumor weights of different treatment groups. Data were presented as the mean ± SD, n=5; **P < 0.01. C) Representative images of H&E, Masson’s trichrome, Sirius Red, and α-SMA staining of tumor tissues at the end of the experiment. Nuclei are stained with DAPI (blue), and α-SMA is shown in green. Scale bar = 100 μm. Data are presented as mean ± SD (n = 5). Statistical significance was determined by unpaired two-tailed Student’s t-test. ns, not significant; **p < 0.01.


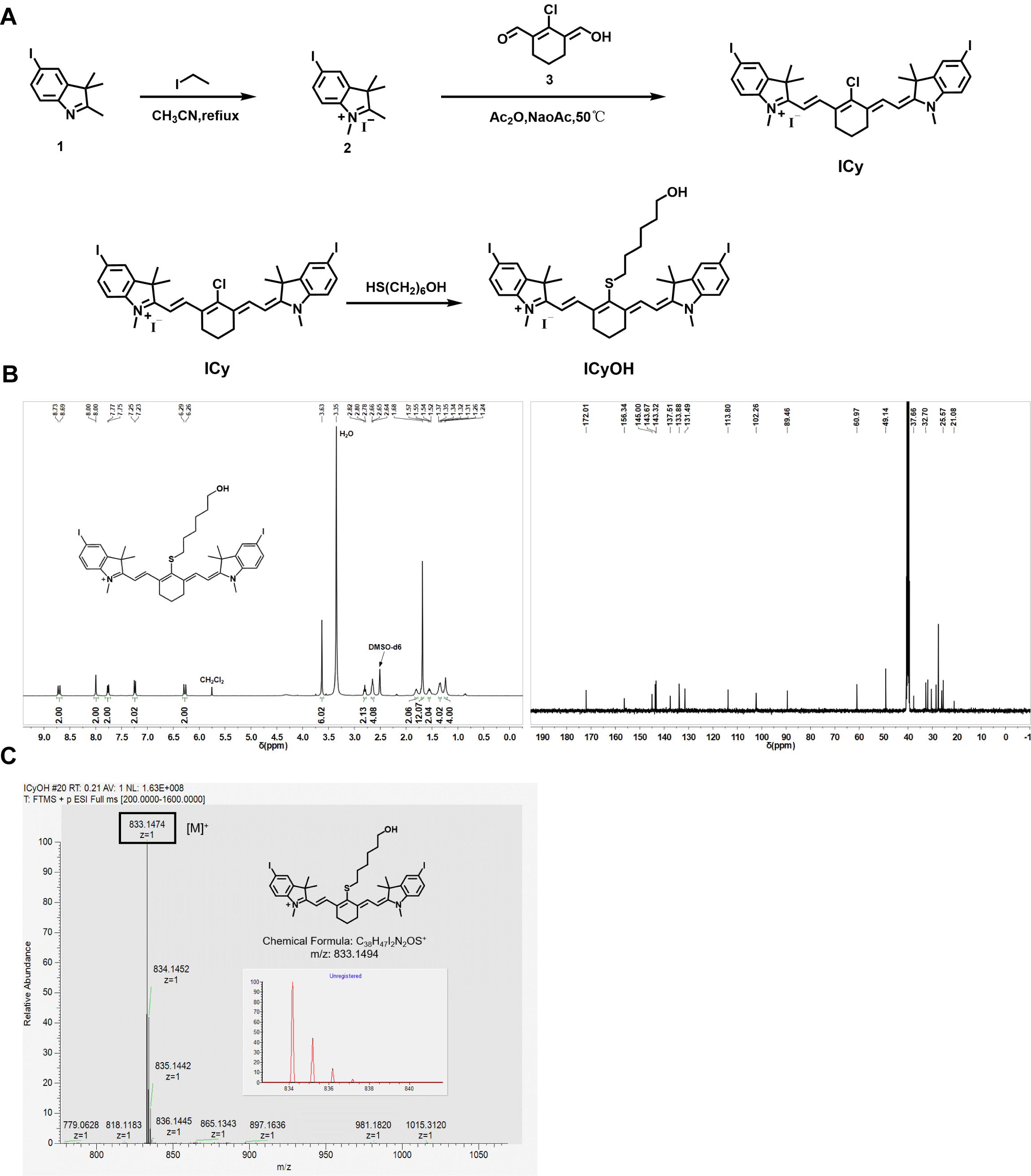


**Figure S5.** A) Synthetic route of ICyOH. B)^1^H and ^13^C NMR spectra of ICyOH, ^1^H NMR (400 MHz, DMSO-*d*₆) and ^13^C NMR (101 MHz, DMSO-*d*₆). C) HR-MS spectrum (ESI，positive mode) of ICyOH.


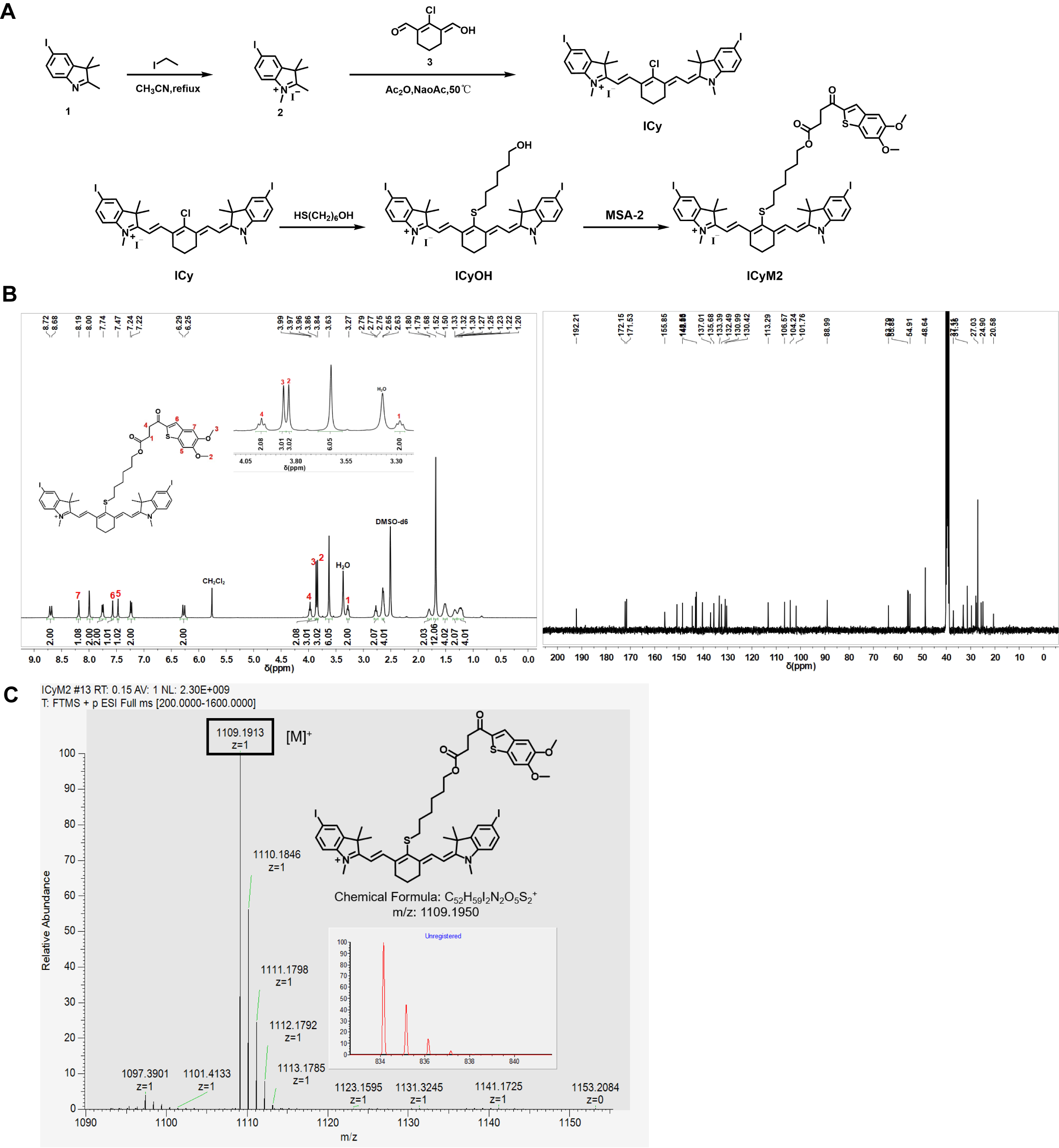


**Figure S6.** A) Synthetic route of ICyM2. B)^1^H and ^13^C NMR spectra of ICyM2, ^1^H NMR (400 MHz, DMSO-*d*_6_) and ^13^C NMR (101 MHz, DMSO-*d*_6_).C) HR-MS spectrum (ESI，positive mode) of ICyM2.


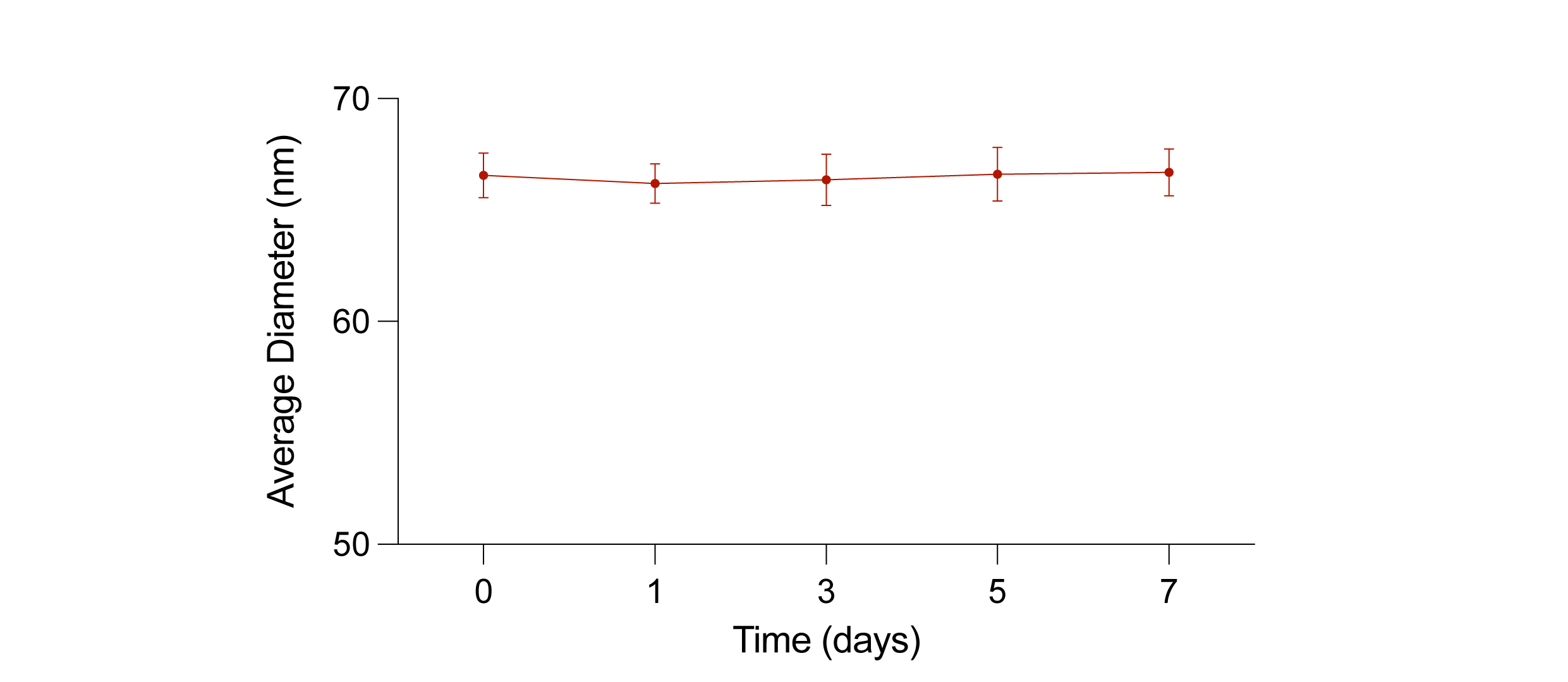


**Figure S7.** Time-dependent colloidal stability of ICyM2 in phosphate-buffered saline (PBS) containing 10% FBS at 37 °C over 7 days. The average hydrodynamic diameter remained stable throughout the incubation period. Data are presented as mean ± SD (n = 3).


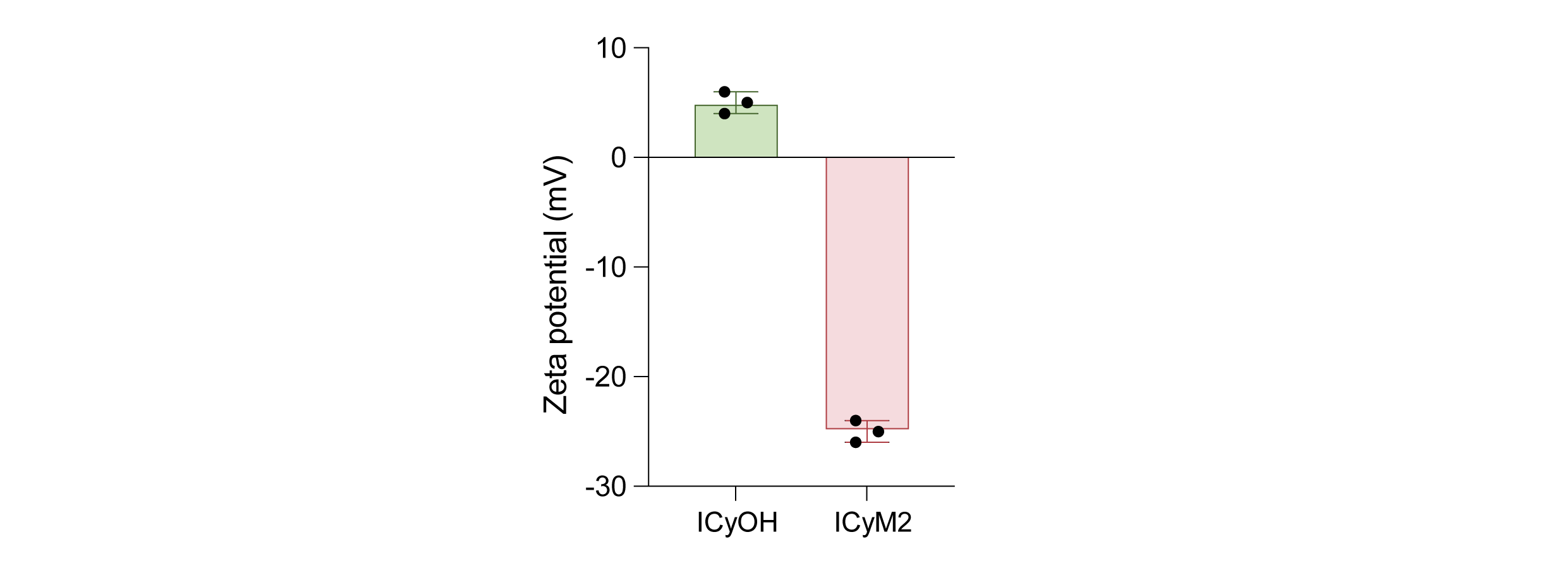


**Figure S8.** Zeta potential of ICyOH and ICyM2.Data are presented as mean ± SD (n = 3).


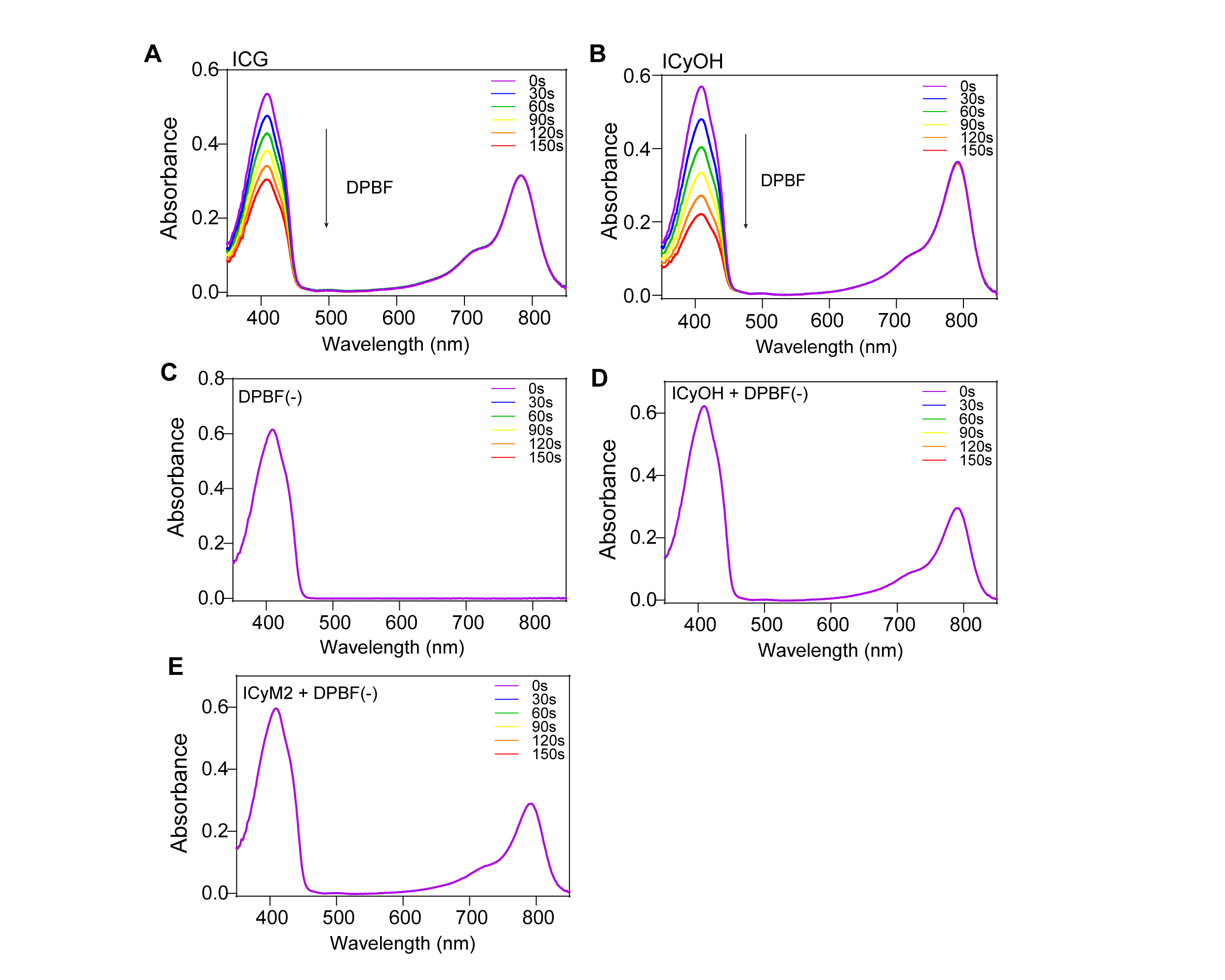


**Figure S9** A, B) DPBF absorption spectra of ICG (A) and ICyOH (B) in methanol under 808 nm laser irradiation (100 mW cm⁻²). C-D) DPBF absorption spectra in methanol under dark conditions: (C) DPBF alone, D) DPBF with ICyOH. E) DPBF with ICyM2.


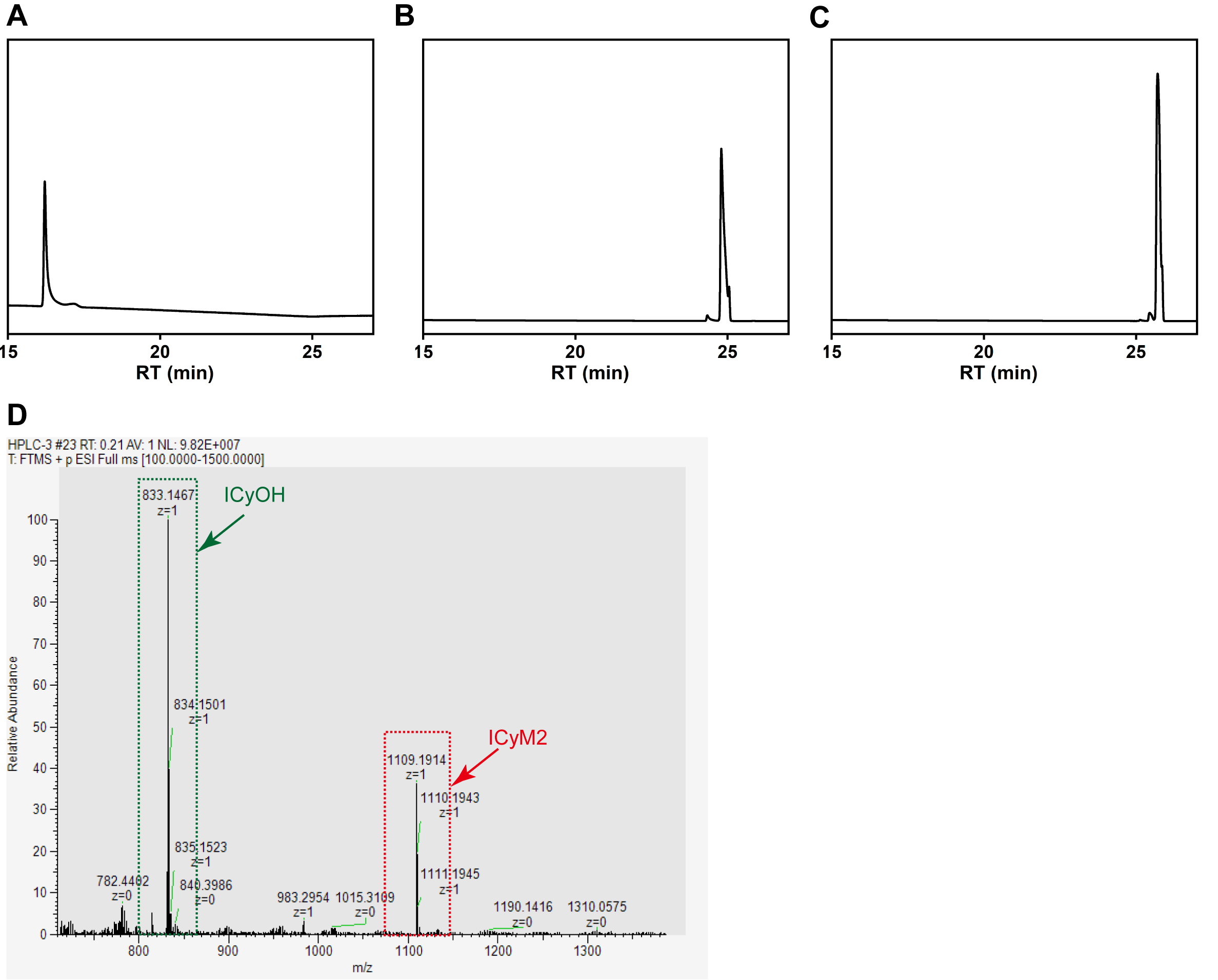


**Figure S10.** A, C) HPLC chromatograms of MSA-2 (A), ICyOH (B), and ICyM2 (C). D) Mass spectrometry characterization of ICyM2 hydrolysis products.


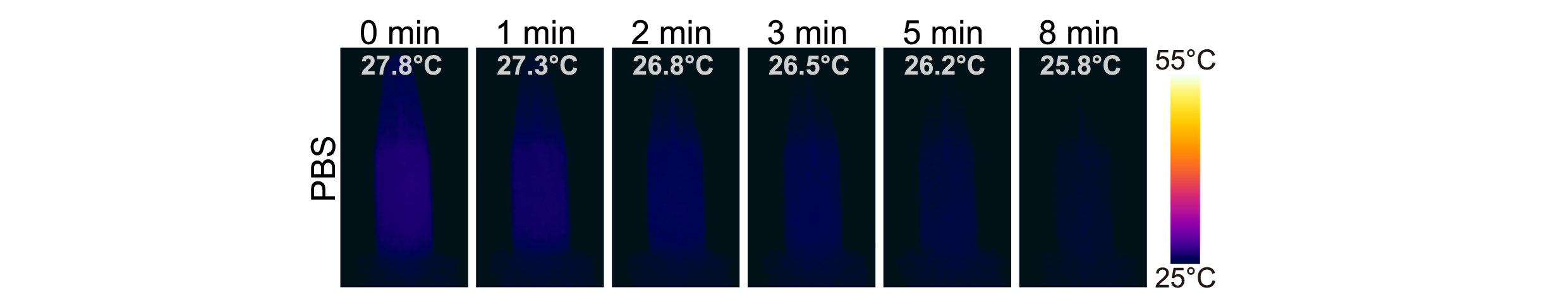


**Figure S11.** Thermal images PBS in methanol under 808 nm laser irradiation (50 µM, 500 mW cm⁻^2^) recorded by FLIR camera.


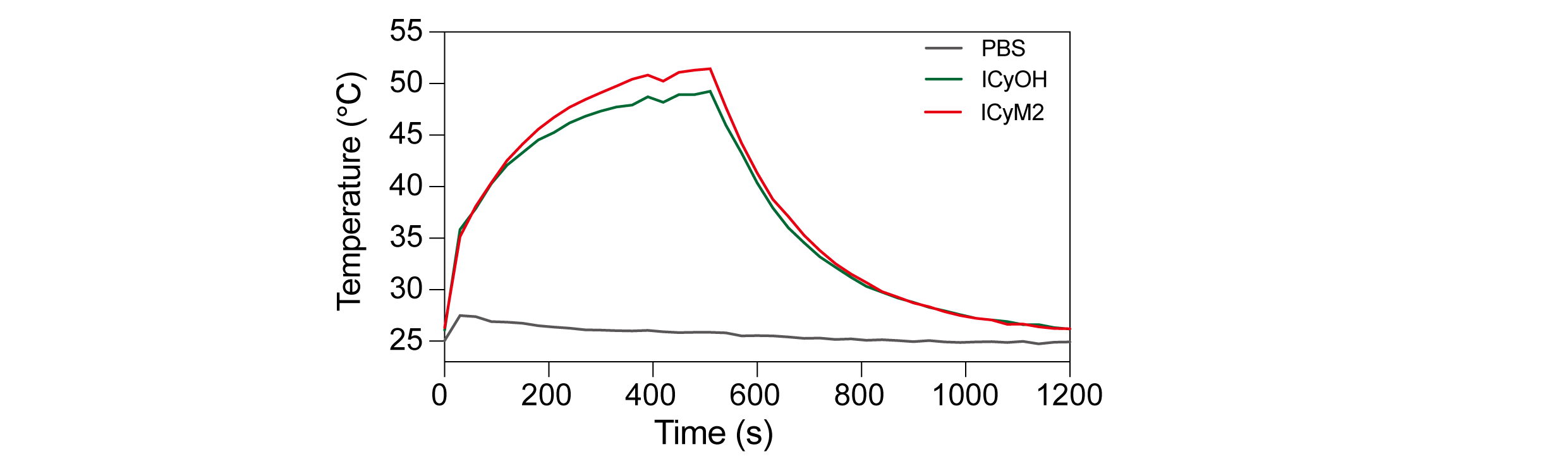


**Figure S12.** Temperature–time curves of ICyOH and ICyM2 in methanol (50 µM) under 808 nm laser irradiation (500 mW cm⁻²) recorded by a FLIR camera.


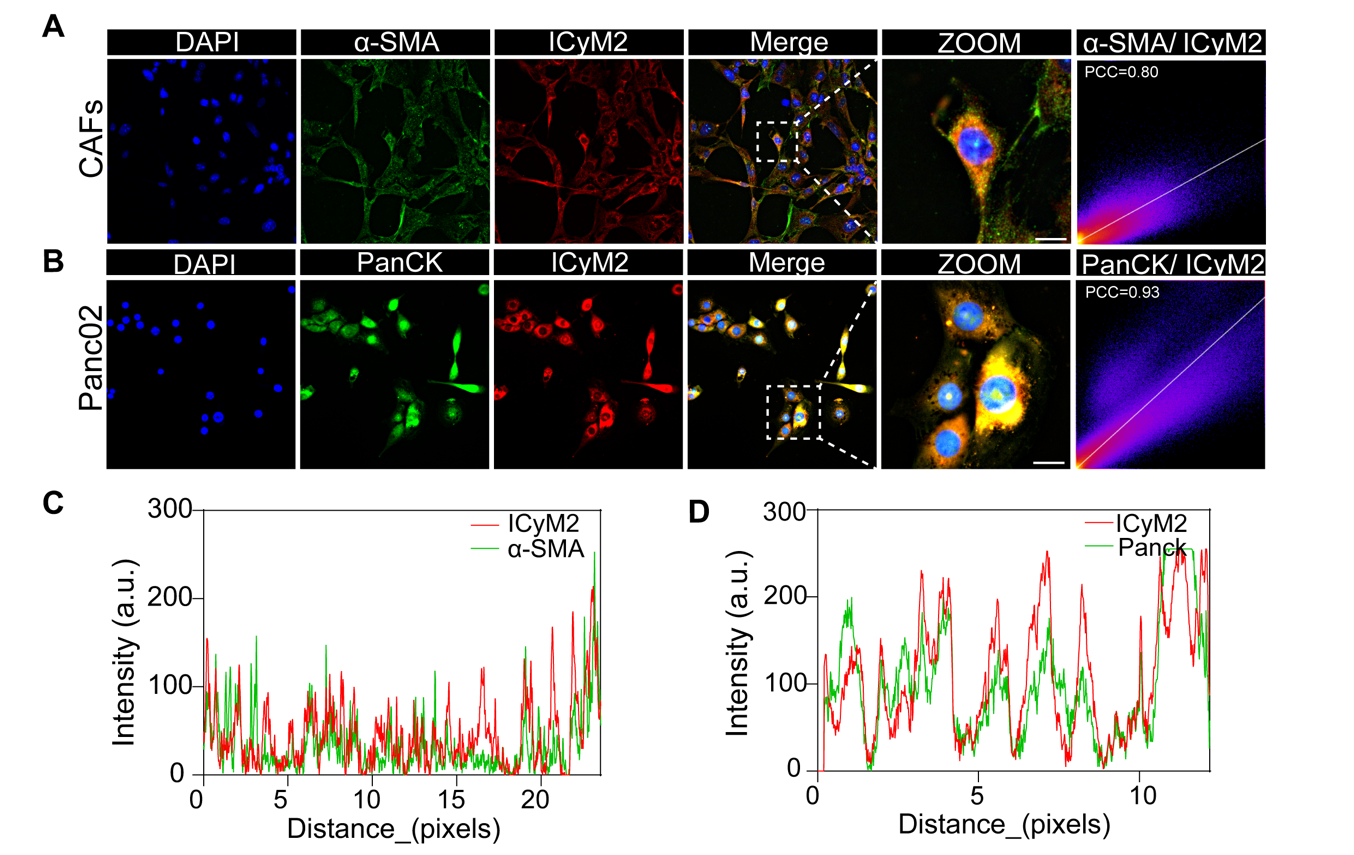


**Figure S13.** A, B) Immunofluorescence co-staining showing ICyM2 accumulation in α-SMA⁺ CAFs and PanCK⁺ tumor cells. Pearson’s correlation coefficients are indicated. Scale bar = 20 µm. C, D) Line-scan intensity profiles showing high spatial overlap between ICyM2 fluorescence and α-SMA or PanCK immunostaining in CAFs (C) and tumor cells (D).


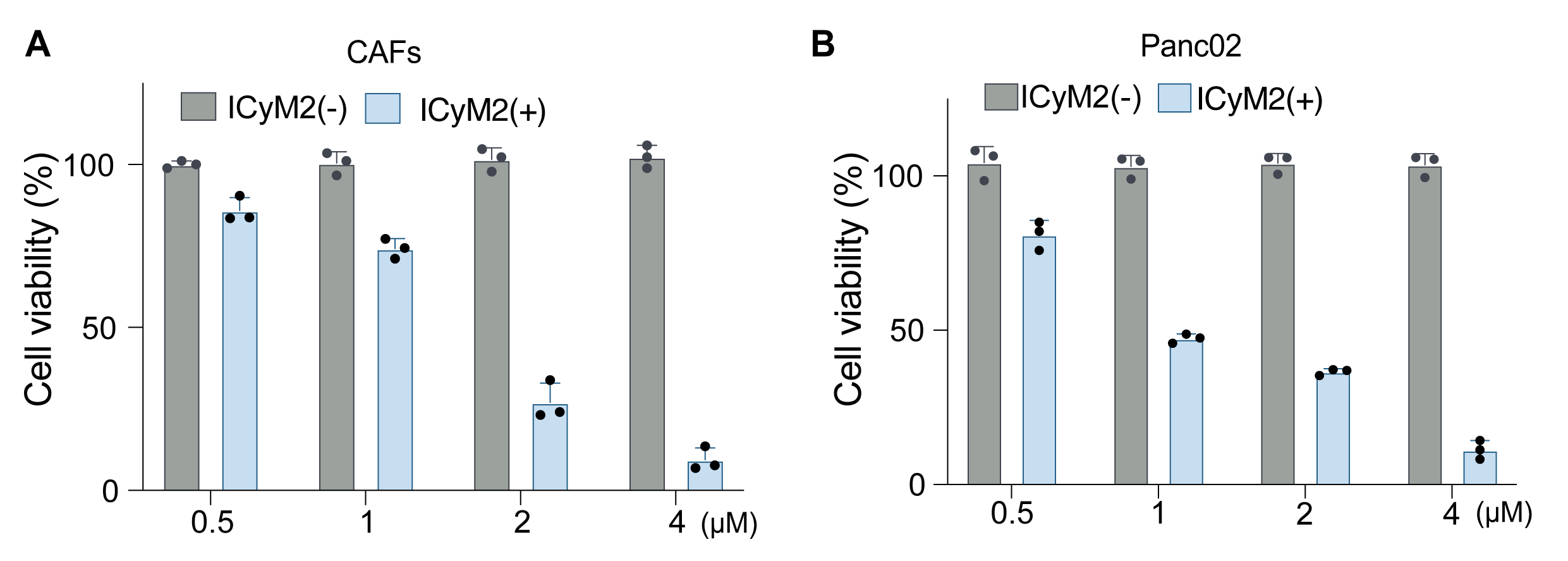


**Figure S14.** (A) CAFs and (B) Panc02 treated with ICyM2 (0.5–4 µM) with or without NIR irradiation (808 nm, 0.5 W cm⁻², 1 min). “ICyM2(+)” denotes irradiated wells; “ICyM2(−)” non-irradiated. Data were presented as the mean ± SD, n=3.


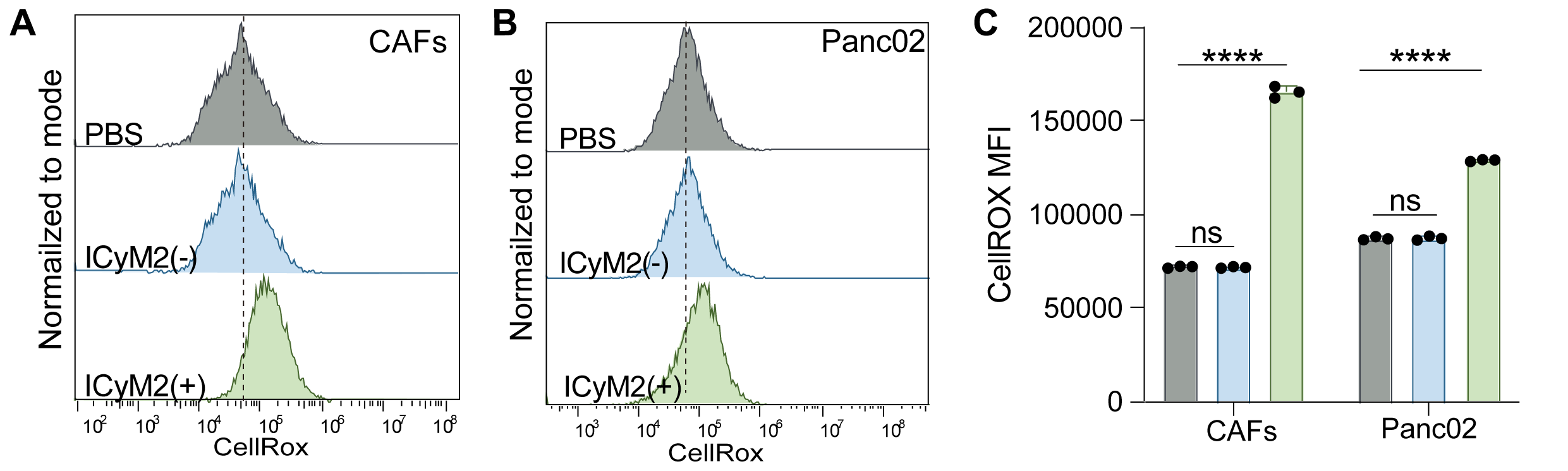


**Figure S15.** (A-B) Flow cytometry of DCFH-DA fluorescence in CAFs and Panc02 after ICyM2 ± NIR (808 nm, 0.5 W·cm⁻², 1 min). “ICyM2(+)” denotes irradiated; “ICyM2(−)” non-irradiated. (C) Quantification of ROS levels. Data are presented as mean ± SD (n = 3); ****P < 0.0001. Data are presented as mean ± SD (n = 3). Statistical significance was determined by one-way ANOVA followed by Tukey’s post hoc test. ns, not significant; ****p < 0.0001.


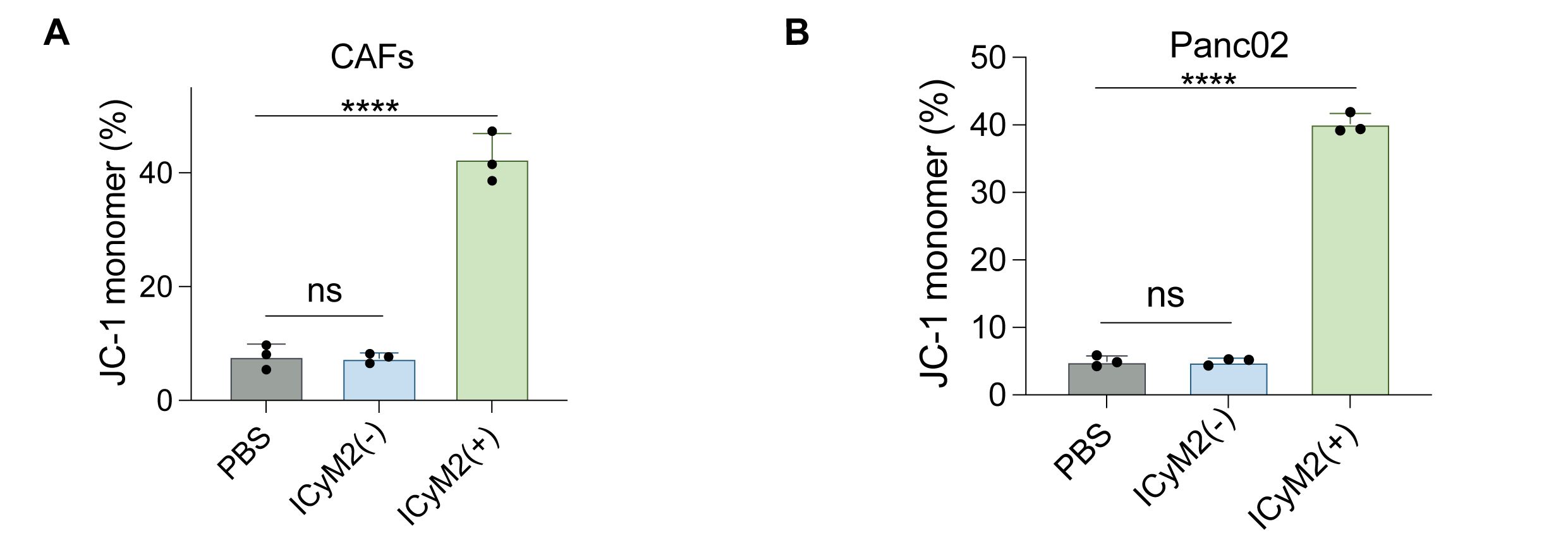


**Figure S16.** CAFs(A) and Panc02(B) treated with ICyM2 with or without NIR irradiation (808 nm, 0.5 W·cm⁻², 1 min). “ICyM2(+)” denotes irradiated; “ICyM2(−)” non-irradiated. Cells were stained with JC-1 and analyzed by flow cytometry. Data are presented as mean ± SD (n = 3). Statistical significance was determined by one-way ANOVA followed by Tukey’s post hoc test. ns, not significant; ****p < 0.0001.


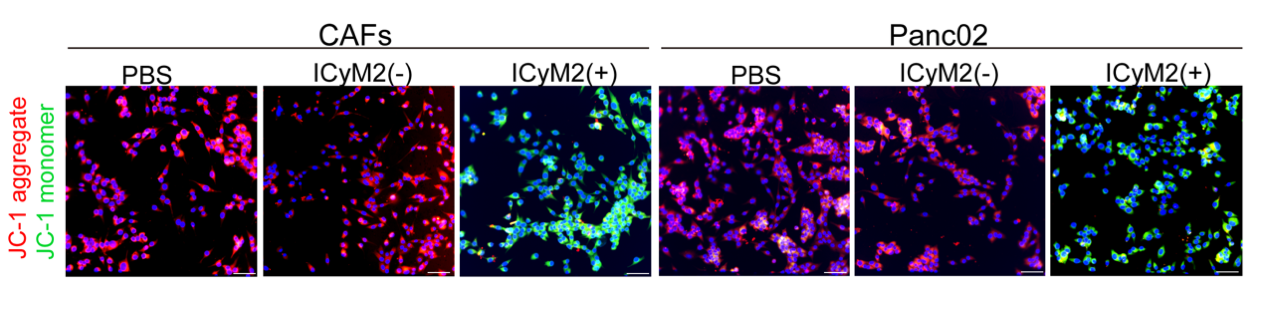


**Figure S17.** Representative images of (A) CAFs and (B) Panc02 after ICyM2 treatment with or without NIR irradiation (808 nm, 0.5 W·cm⁻², 1 min). “ICyM2(+)” denotes irradiated; “ICyM2(−)” non-irradiated. Scale bar = 100 μm.


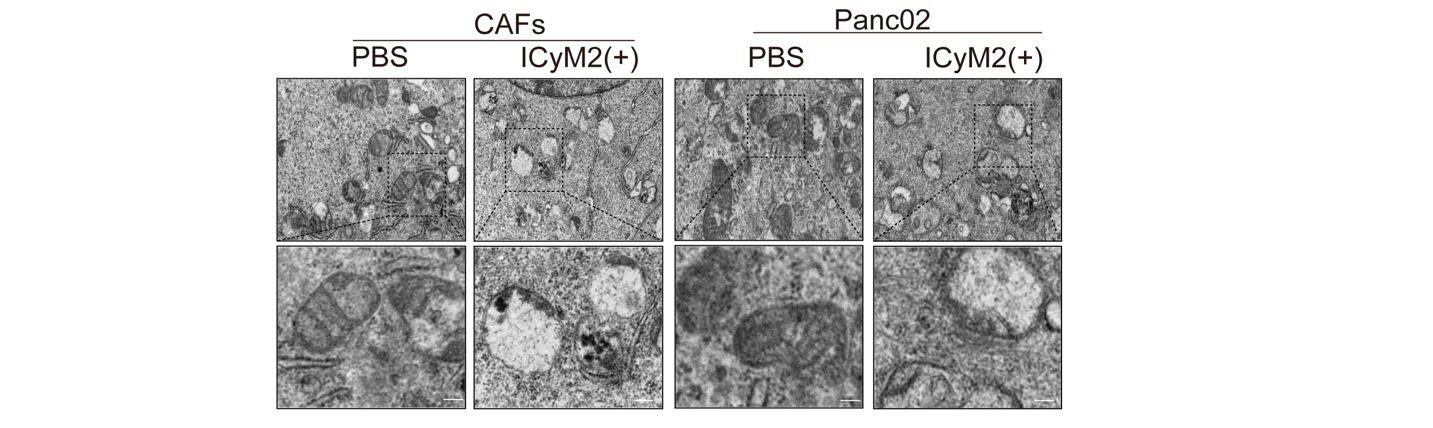


**Figure S18.** Transmission electron microscopy (TEM) of CAFs and Panc02 after ICyM2 ± NIR (808 nm, 0.5 W·cm⁻², 1 min). Mitochondrial injury—cristae disruption, matrix rarefaction, and swelling—is evident in ICyM2(+) compared with PBS. Scale bar = 150 nm.


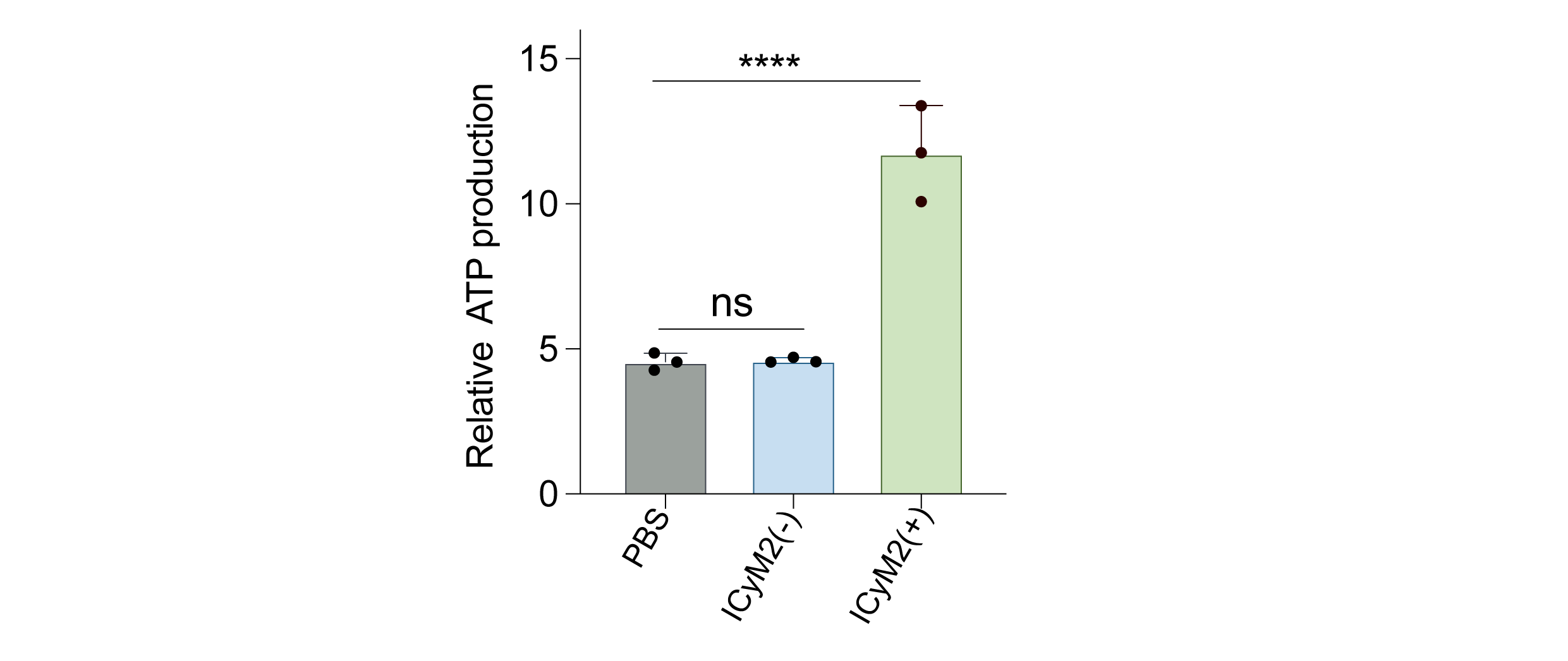


**Figure S19.** Quantification of extracellular ATP release from Panc02 cells under the indicated treatments. The ICyM2 + NIR group (808 nm, 0.5 W·cm⁻², 1 min) showed the highest extracellular ATP compared with PBS and ICyM2(−). Data are presented as mean ± SD (n = 3). Statistical significance was determined by one-way ANOVA followed by Tukey’s post hoc test. ns, not significant; ****p < 0.0001.


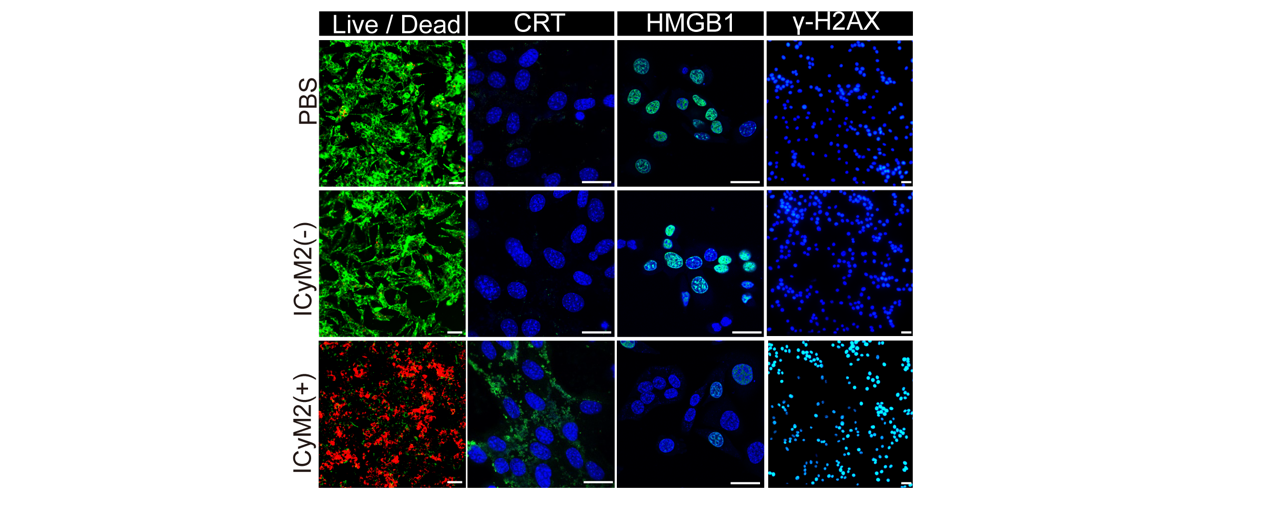


**Figure S20.** Representative images of tumor cells after ICyOH + NIR treatment, showing increased cell death (Live/Dead staining) and hallmark features of ICD, including ecto-CRT exposure, HMGB1 translocation, and γ-H2AX foci formation. Scale bar = 50 µm.


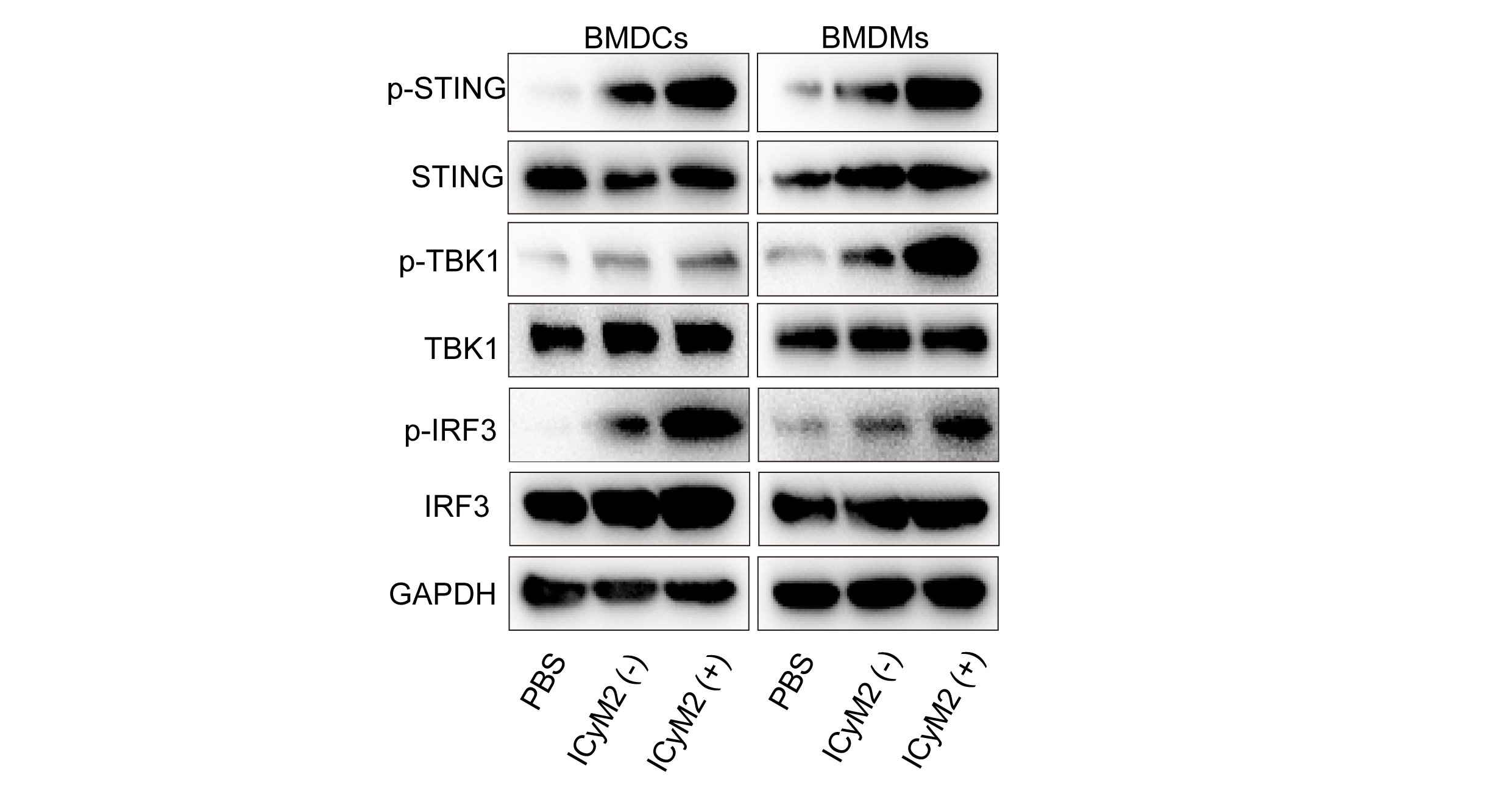


**Figure S21.** Western blot analysis of STING pathway activation in BMDCs and BMDMs following the indicated treatments. Representative blots of phosphorylated STING (p-STING), total STING, phosphorylated TBK1 (p-TBK1), total TBK1, phosphorylated IRF3 (p-IRF3), and total IRF3 are shown. Cells were treated with ICyM2 with or without near-infrared (NIR) irradiation (808 nm, 0.5 W·cm⁻², 1 min). GAPDH served as a loading control.


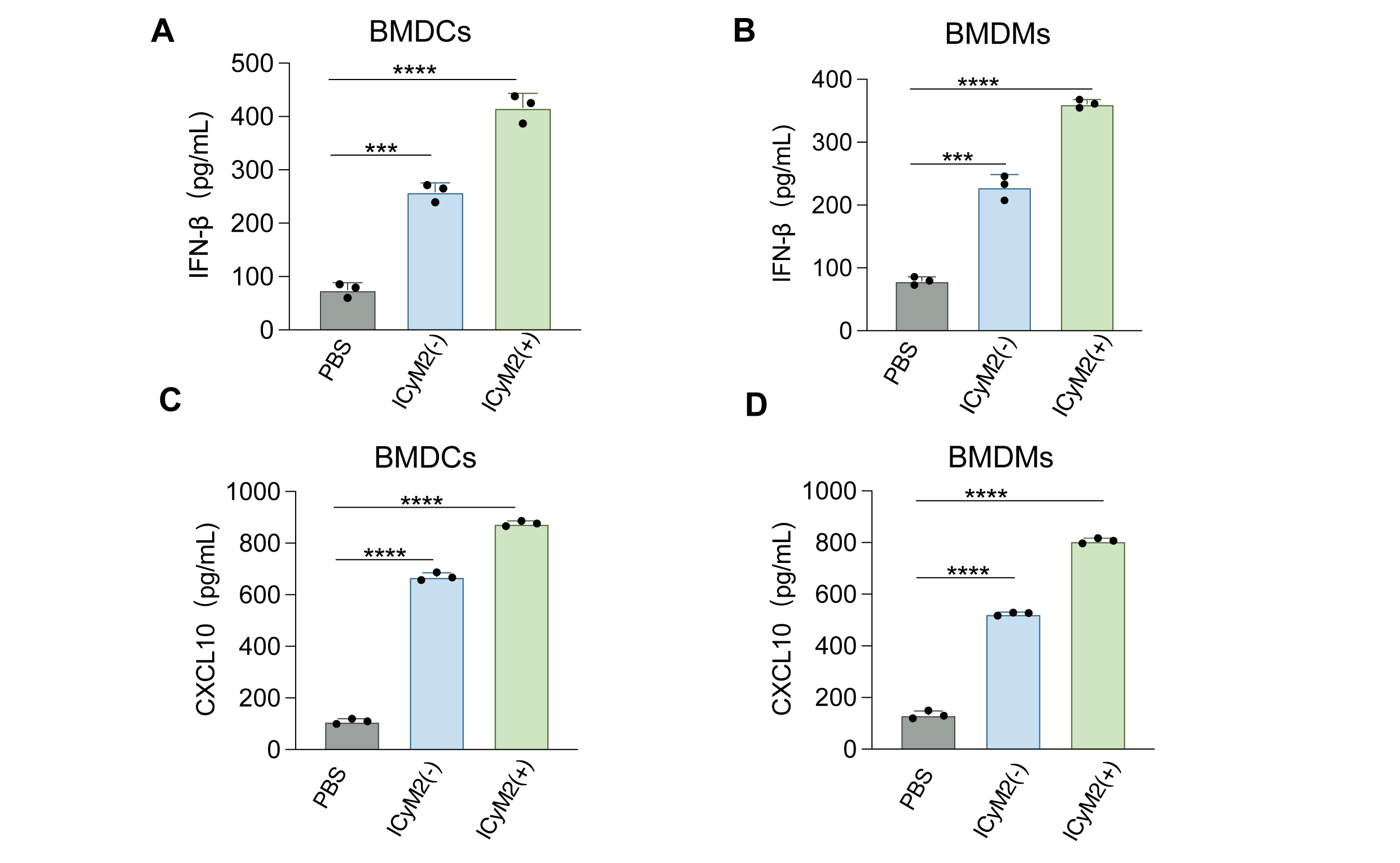


**Figure S22.** ELISA quantification of IFN-β (A, B) and CXCL10 (C, D) secretion from BMDCs (A, C) and BMDMs (B, D) following the indicated treatments (PBS, ICyM2 without NIR, and ICyM2 with NIR). Data are presented as mean ± SD (n = 3). Statistical significance was determined using one-way ANOVA with Tukey’s post hoc test; ***P < 0.001, ****P < 0.0001.


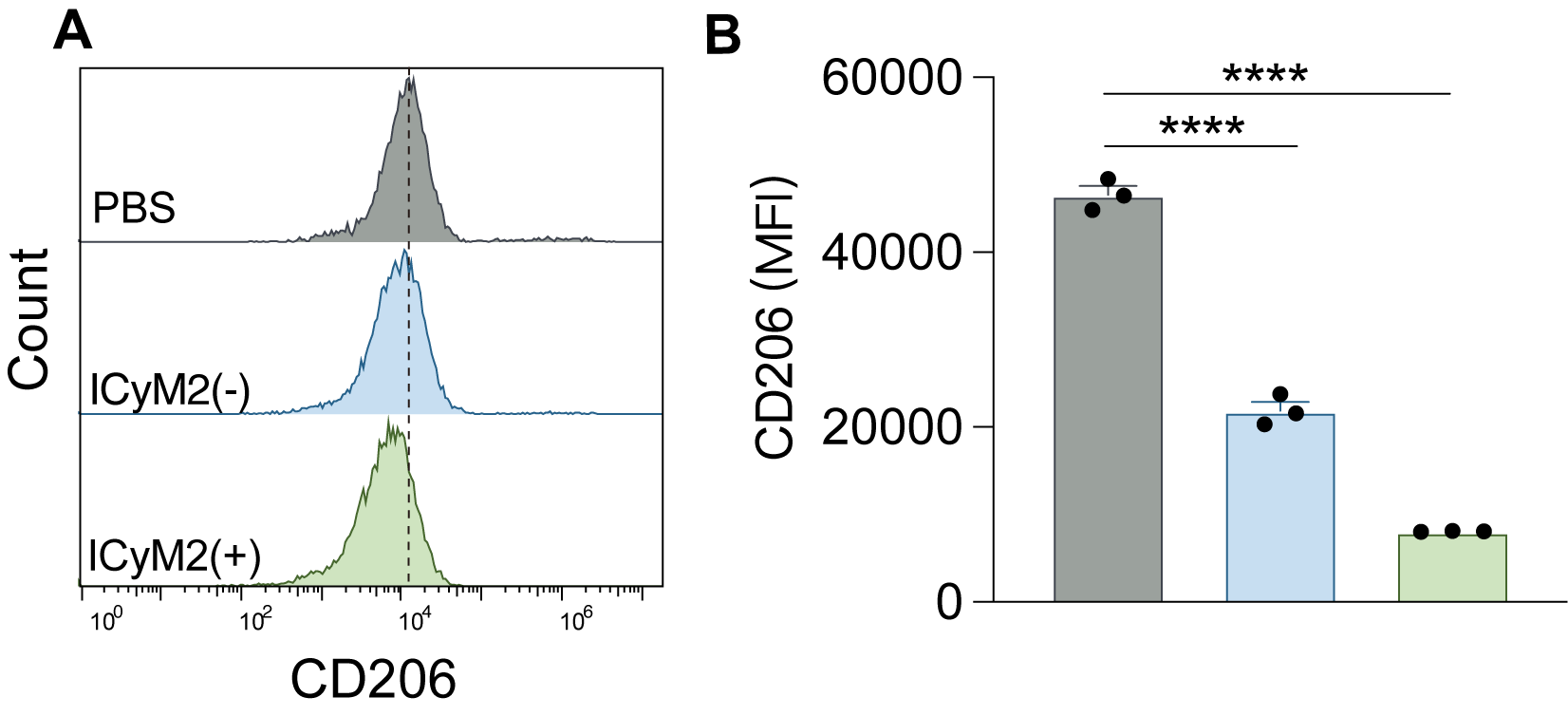


**Figure S23.** ELISA quantification of IFN-β (A, B) and CXCL10 (C, D) secretion from BMDCs (A, C) and BMDMs (B, D) following the indicated treatments (PBS, ICyM2 without NIR, and ICyM2 with NIR). Data are presented as mean ± SD (n = 3). Statistical significance was determined using one-way ANOVA with Tukey’s post hoc test; ****P < 0.0001.


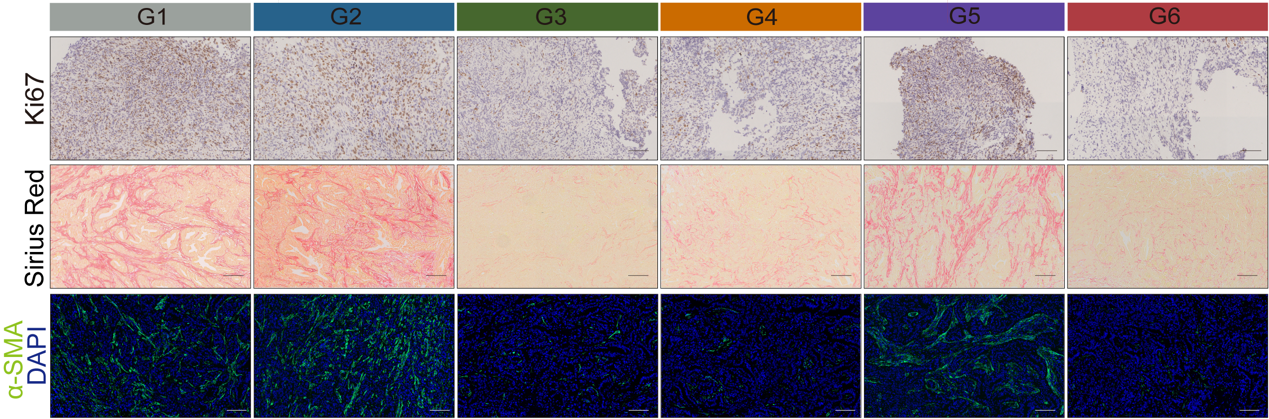


**Figure S24.** Tumor sections stained for Ki67, Sirius Red, and α-SMA (green) with DAPI (blue) to assess proliferation and stromal composition. Scale bar = 100 μm.


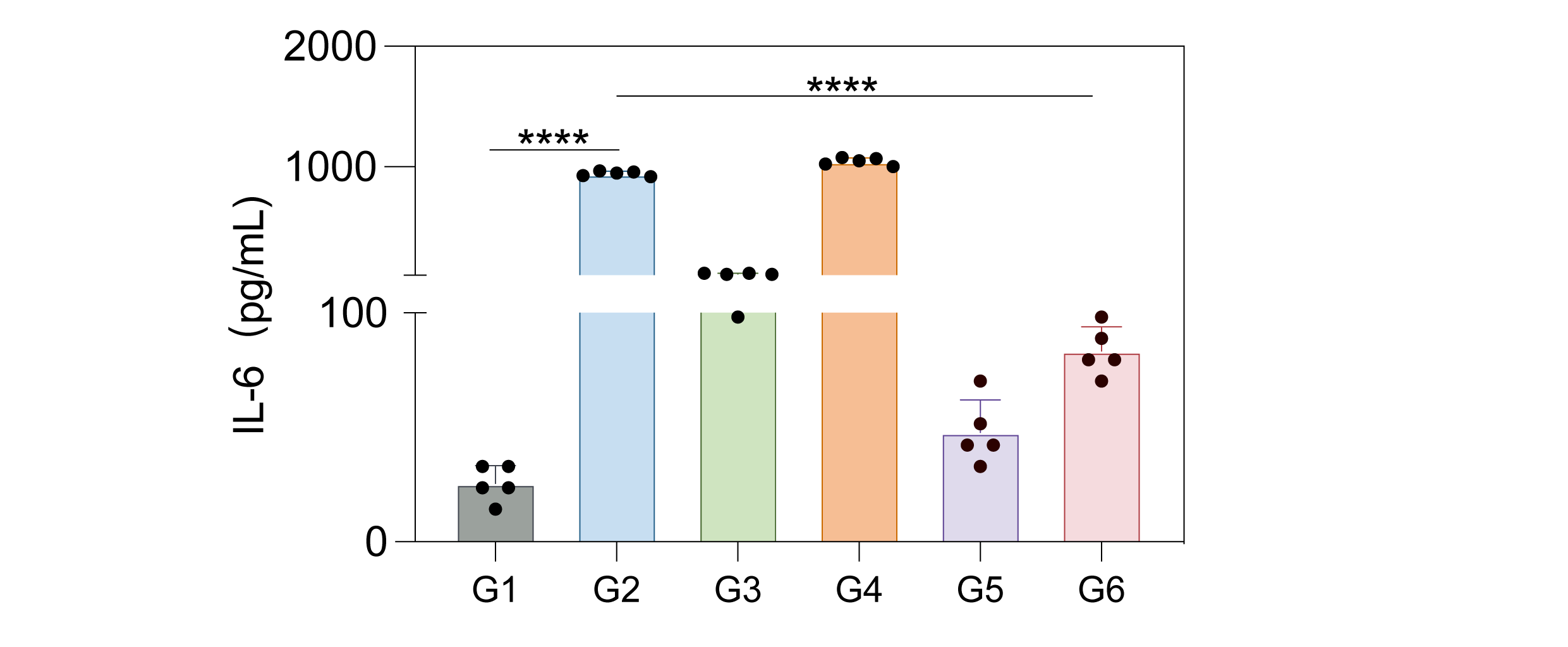


**Figure S25.** Serum IL-6 levels measured 6 h after treatment in six groups: PBS (G1), free MSA-2 (G2), ICyOH + Laser (G3), ICyOH + MSA-2 + Laser (G4), ICyM2 without irradiation (G5), and ICyM2 + Laser (G6). Quantification was performed by ELISA. Data are presented as mean ± SD (n = 5). Statistical analysis was performed using one-way ANOVA followed by Tukey’s post hoc test. ****P < 0.0001.


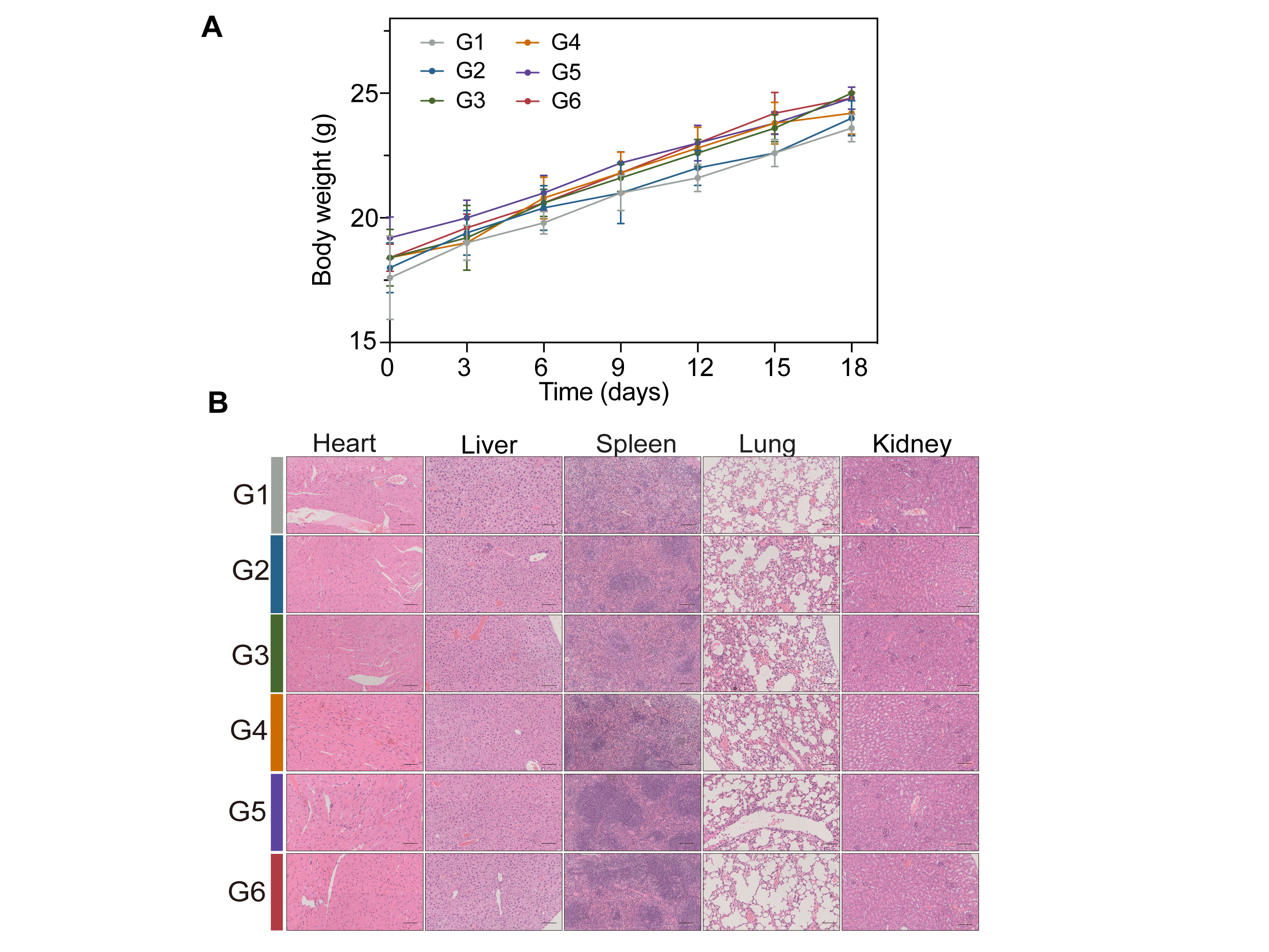


**Figure S26.** A) Tumor weights of different treatment groups (n = 5)**.** B) H&E staining of major organs of treated mice, including heart, spleen, lung, and kidney. Scale bar = 100 μm.


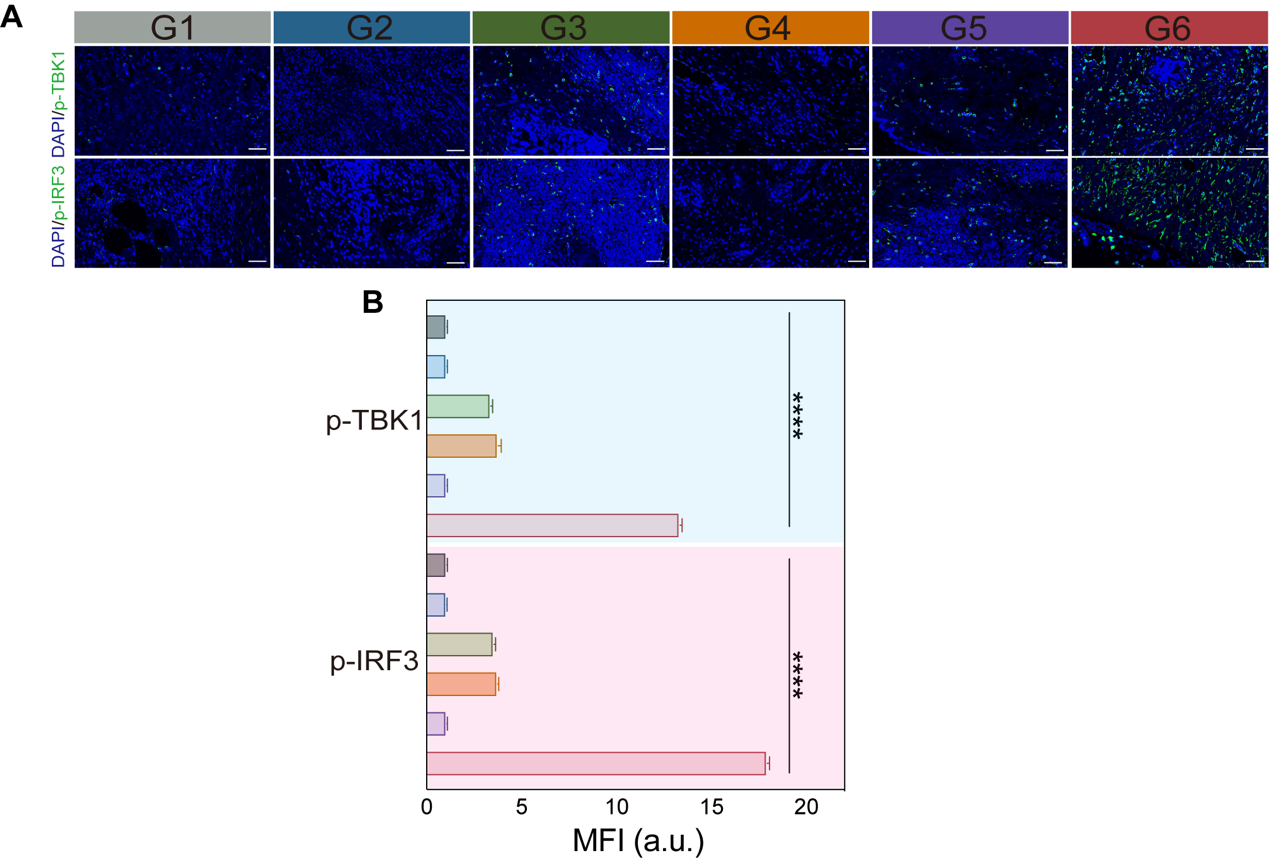


**Figure S27**. A) Representative immunofluorescent images and B) quantification of the expression levels of p-TBK1, and p-IRF3 in primary tumor tissues after different treatments. Scale bar = 50 µm. Data are presented as mean ± SD (n = 3). Statistical significance was determined using one-way ANOVA with Tukey’s post hoc test; ****P < 0.0001.


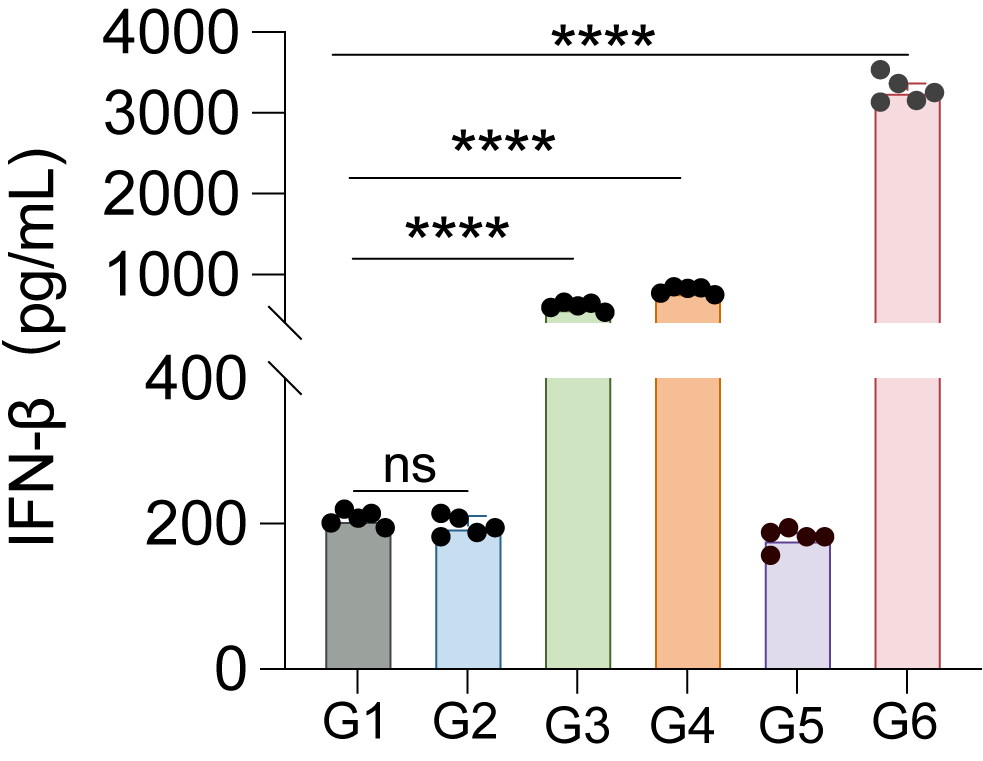


**Figure S28.** Serum cytokine levels of IFN-β in mice after different treatments. Data are presented as mean ± SD (n = 3); Data are presented as mean ± SD (n = 5). Statistical significance was determined using one-way ANOVA with Tukey’s post hoc test; ****P < 0.0001.


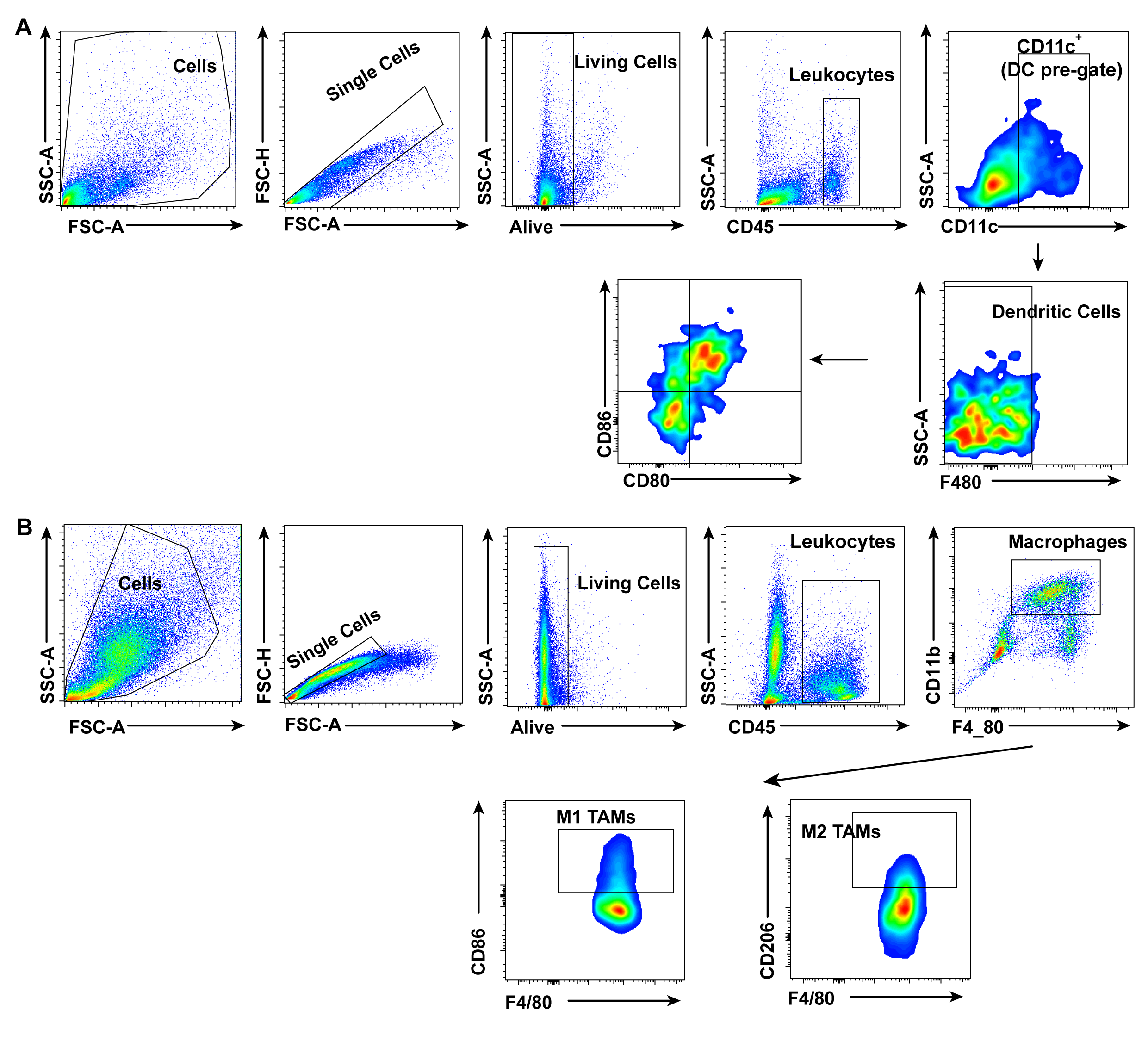


**Figure S29.** Gating strategies for analysis of tumor-infiltrating myeloid populations. A) Mature DCs were identified from CD45⁺CD11c⁺F480^-^ cells. B) M1- and M2-like TAMs were distinguished within the CD11b⁺F4/80⁺ population.


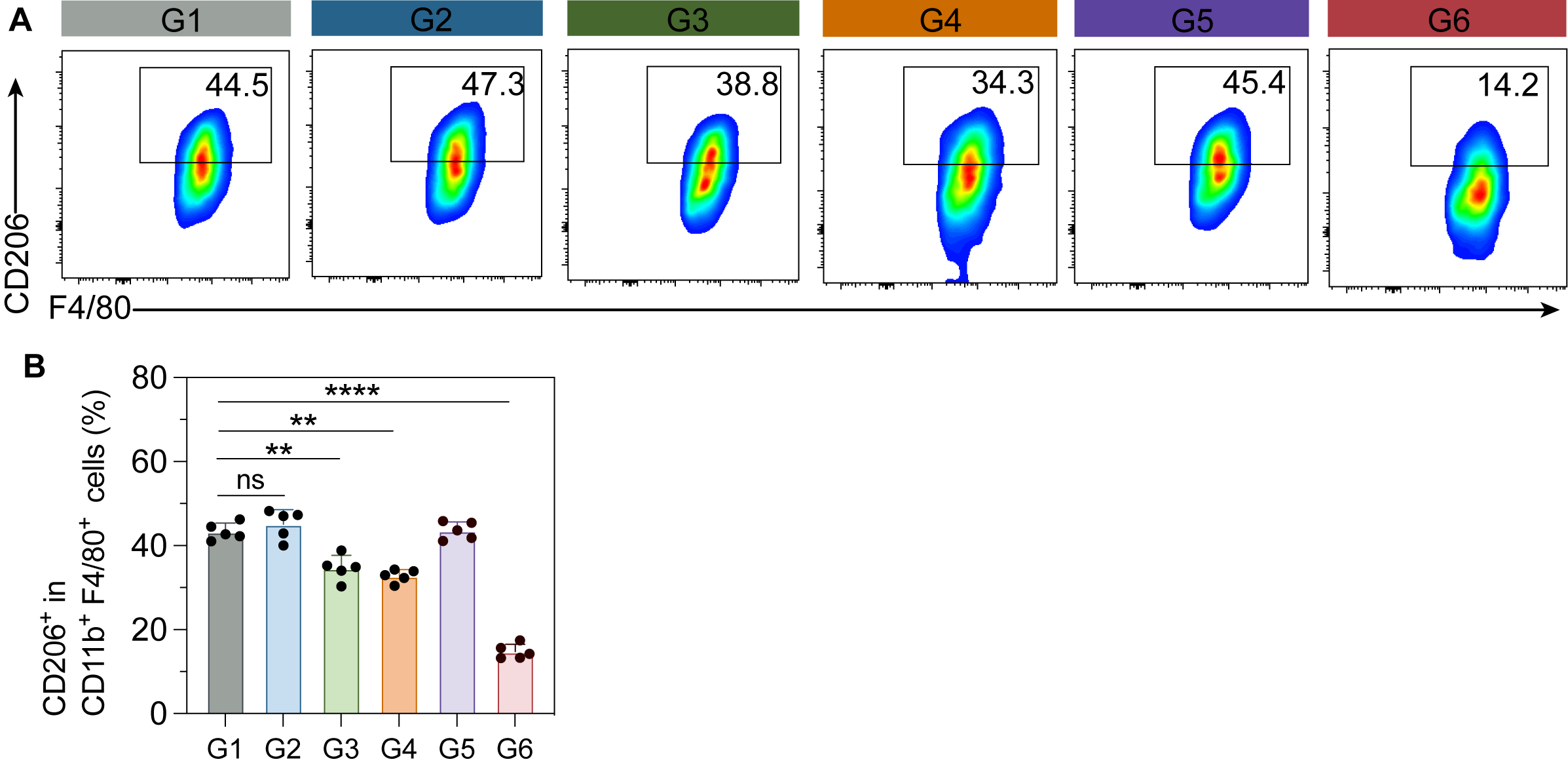


**Figure S30.** A) Flow cytometry analysis of M2‑like macrophages. B) Quantification of M2‑like macrophages. Data are presented as mean ± SD (n = 5). Statistical significance was determined using one-way ANOVA with Tukey’s post hoc test; **p < 0.01, ****p < 0.0001, ns indicates not significant.


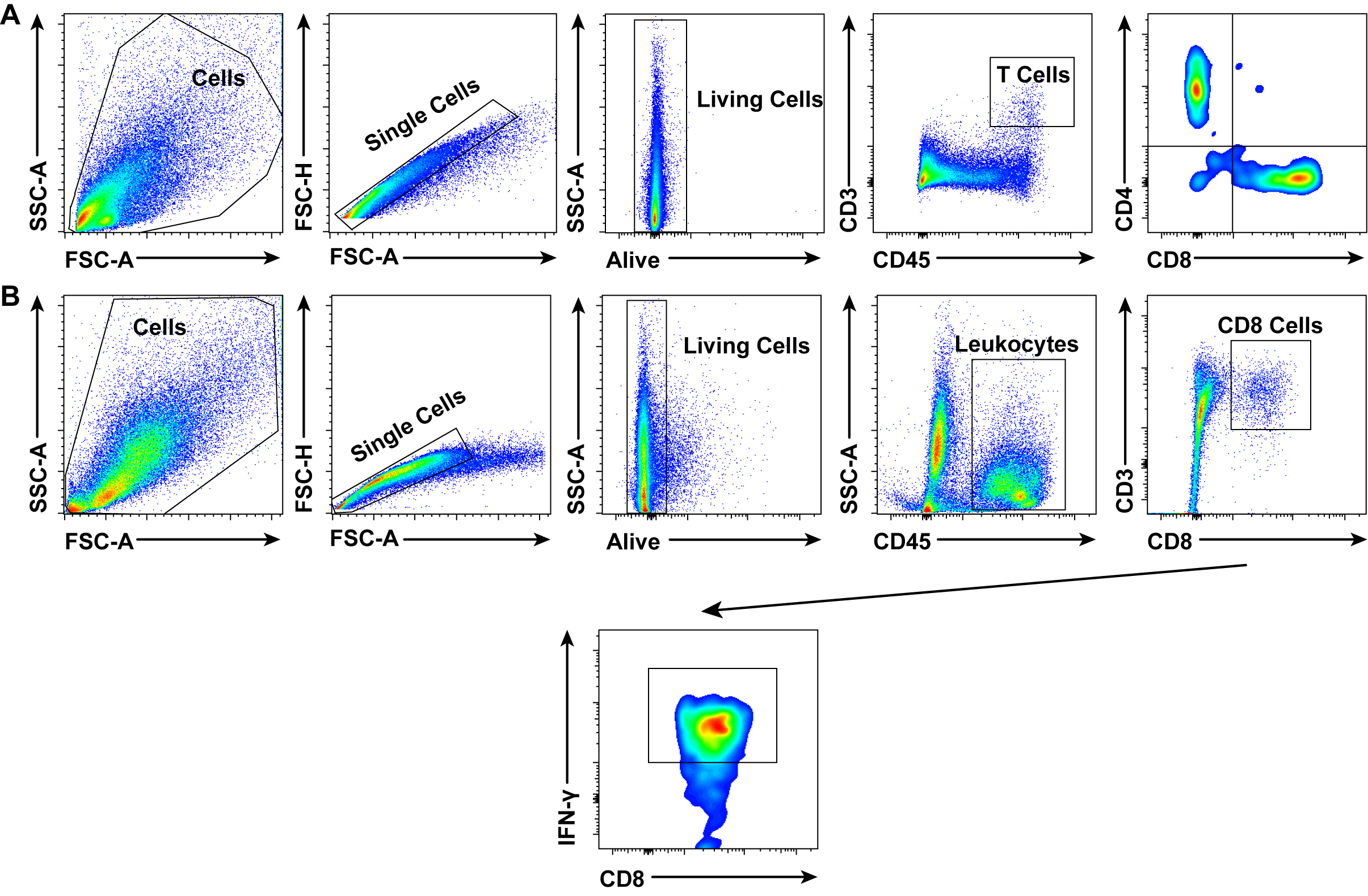


**Figure S31.** Gating strategies were used to investigate proportion of various immune cells in tumors of mice. A) CD8+ T cells and CD4+ T cells were gated from CD45+ CD3+ T cells in tumors. B) IFN-γ production were gated from CD3+CD8+ cells in tumors.


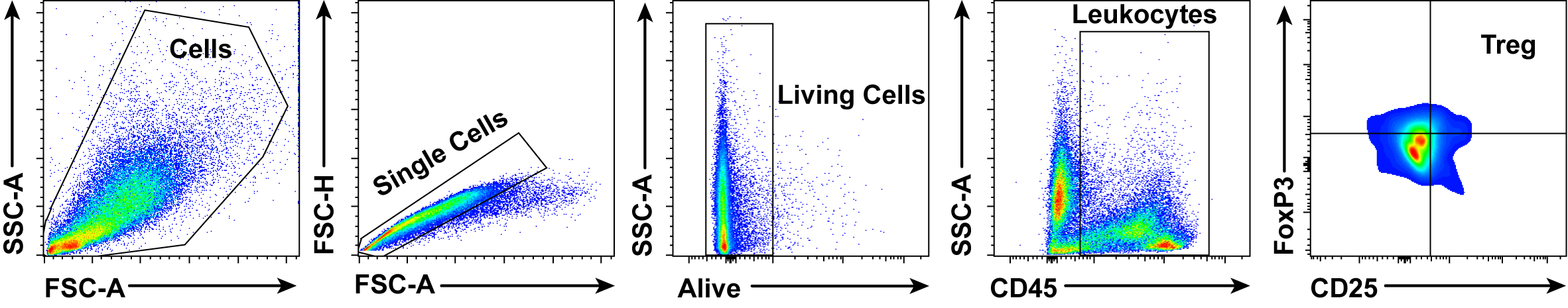


**Figure S32**. Treg cells were gated from CD45+ cells in tumors.


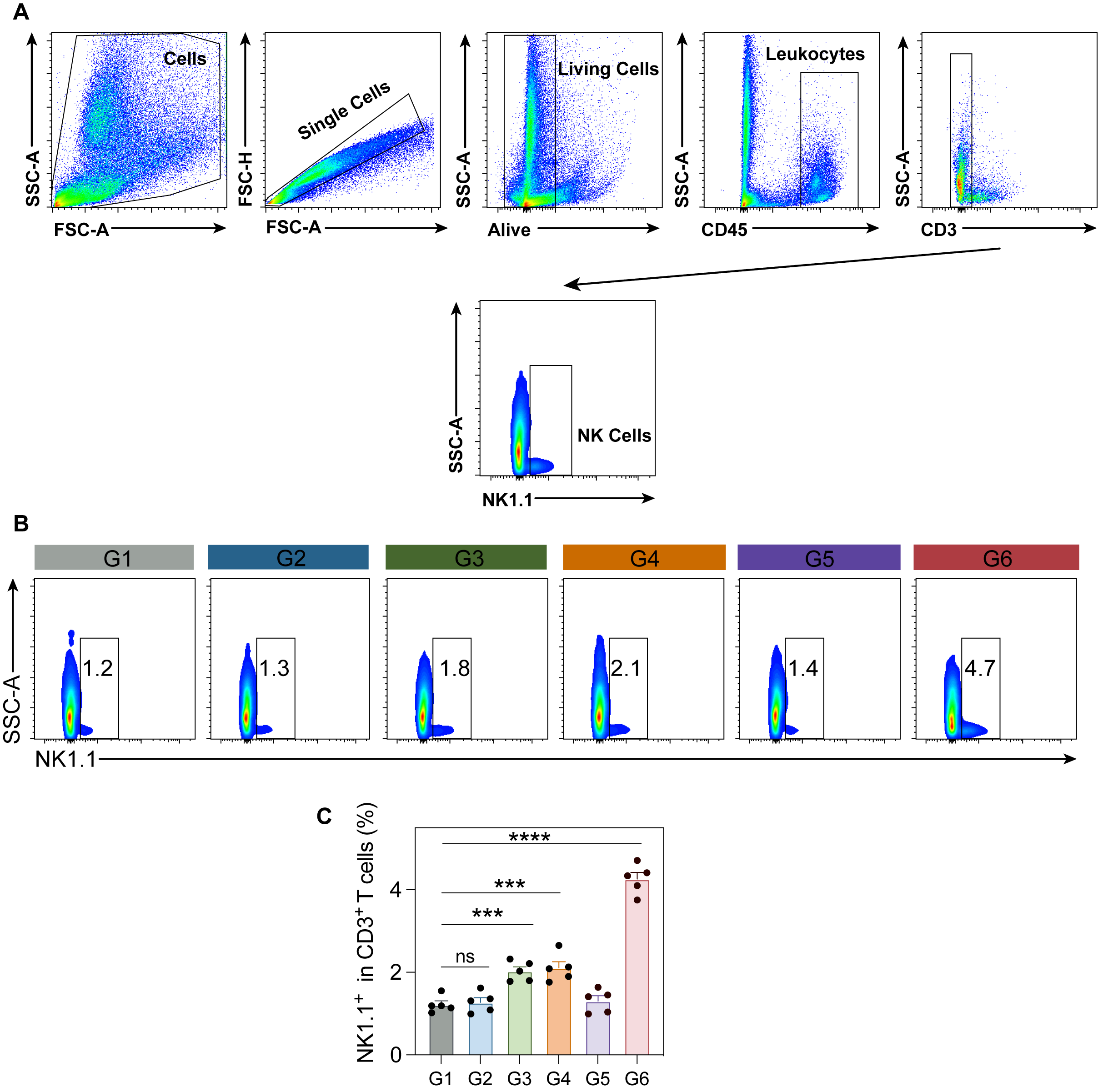


**Figure S33**. A) ΝΚ cells were gated from CD3- cells in tumors. B) Flow cytometry analysis of NK cells. C) Quantification of ΝΚ cells Data are presented as mean ± SD (n = 5). Statistical significance was determined using one-way ANOVA with Tukey’s post hoc test; ***p < 0.001, ****p < 0.0001, ns indicates not significant.


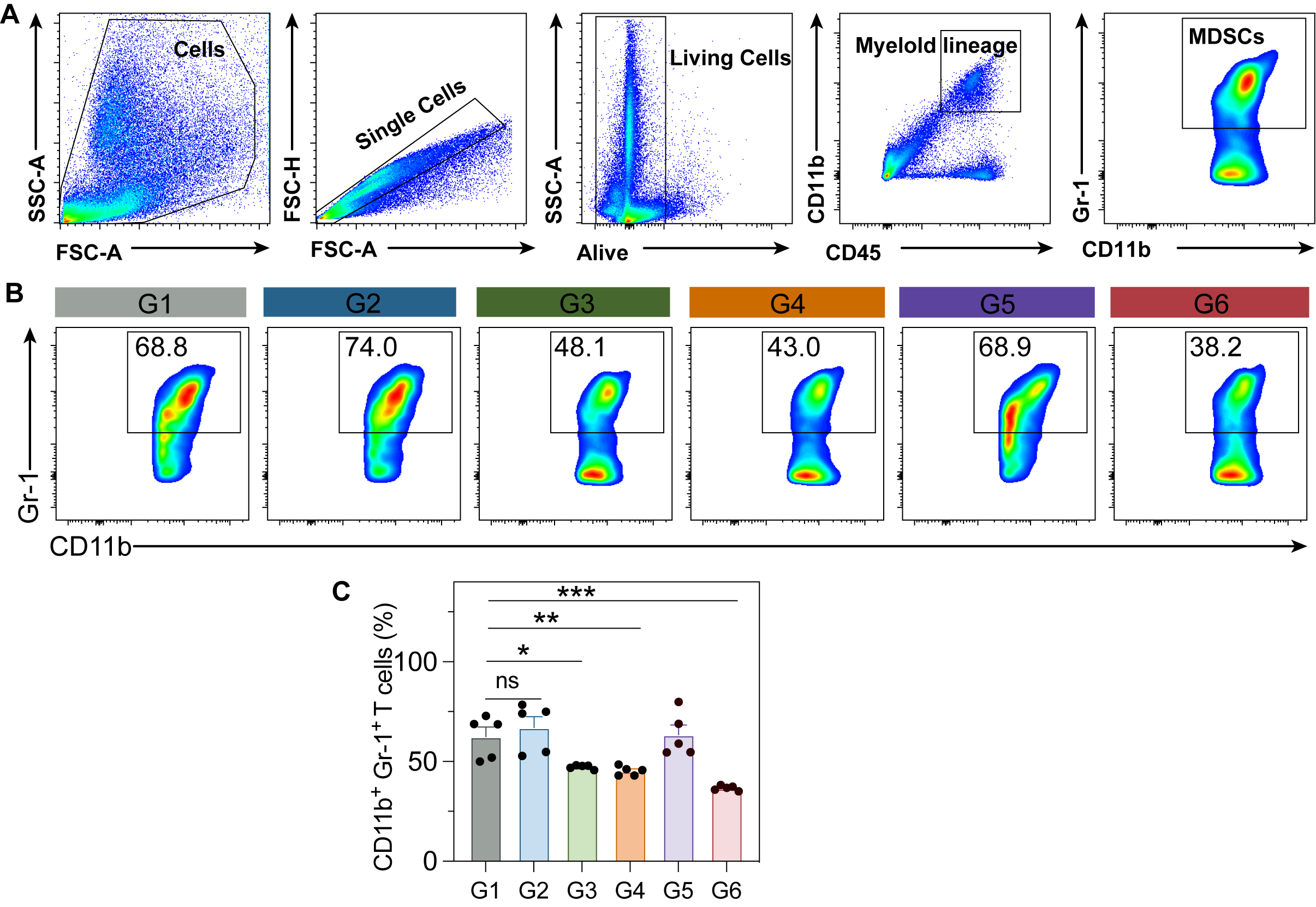


**Figure S34**. A) MDSC cells were gated from CD45+CD11b+ cells in tumors. B) Flow cytometry analysis of MDSC cells. C) Quantification of MDSC cells. Data are presented as mean ± SD (n = 5). Statistical significance was determined using one-way ANOVA with Tukey’s post hoc test; *P < 0.05, ** P < 0.01, *** P < 0.001, ns indicates not significant.

**
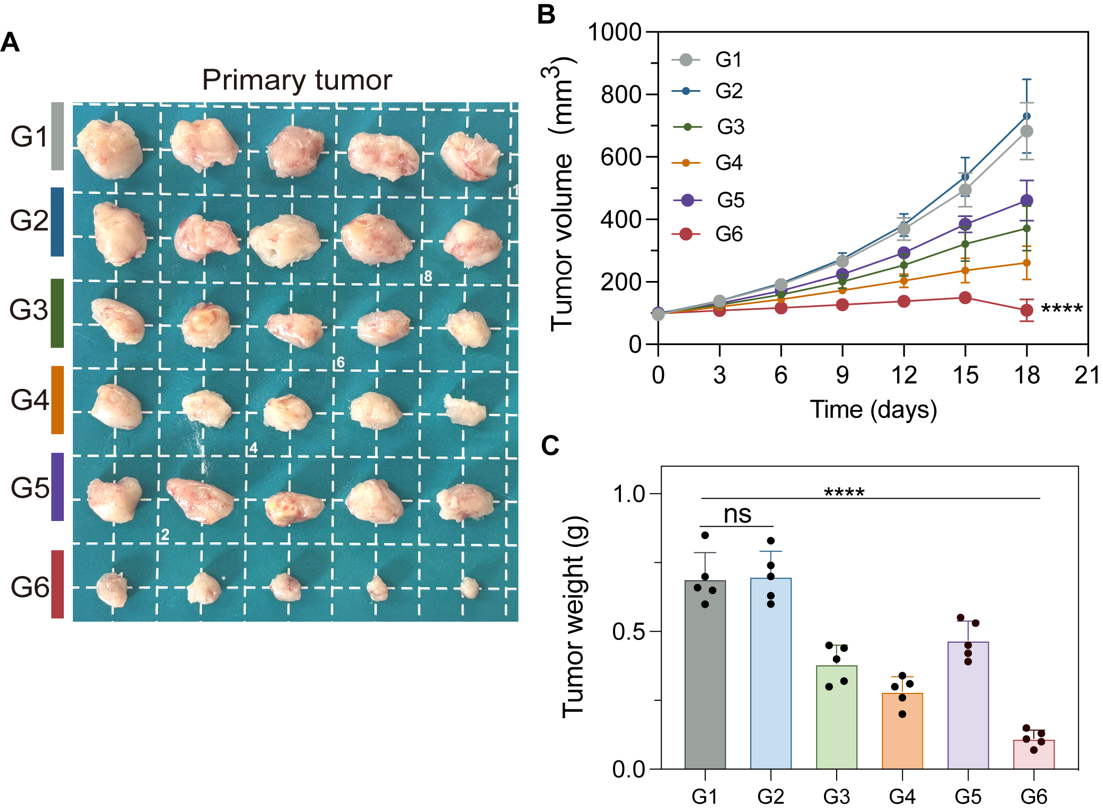
**

**Figure S35.** A) tumor photographs. B) Tumor growth curve. C) tumor weights of different treatment groups (n = 5 mice)**.** Data are presented as mean ± SD (n = 5). Statistical significance was determined using one-way ANOVA with Tukey’s post hoc test; ****P < 0.0001, ns indicates not significant.


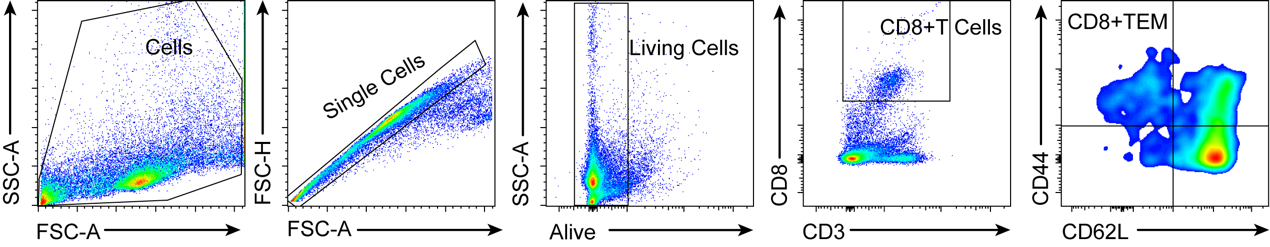


**Figure S36.** Effector memory T cells were gated from CD3+CD8+ cells in spleens.


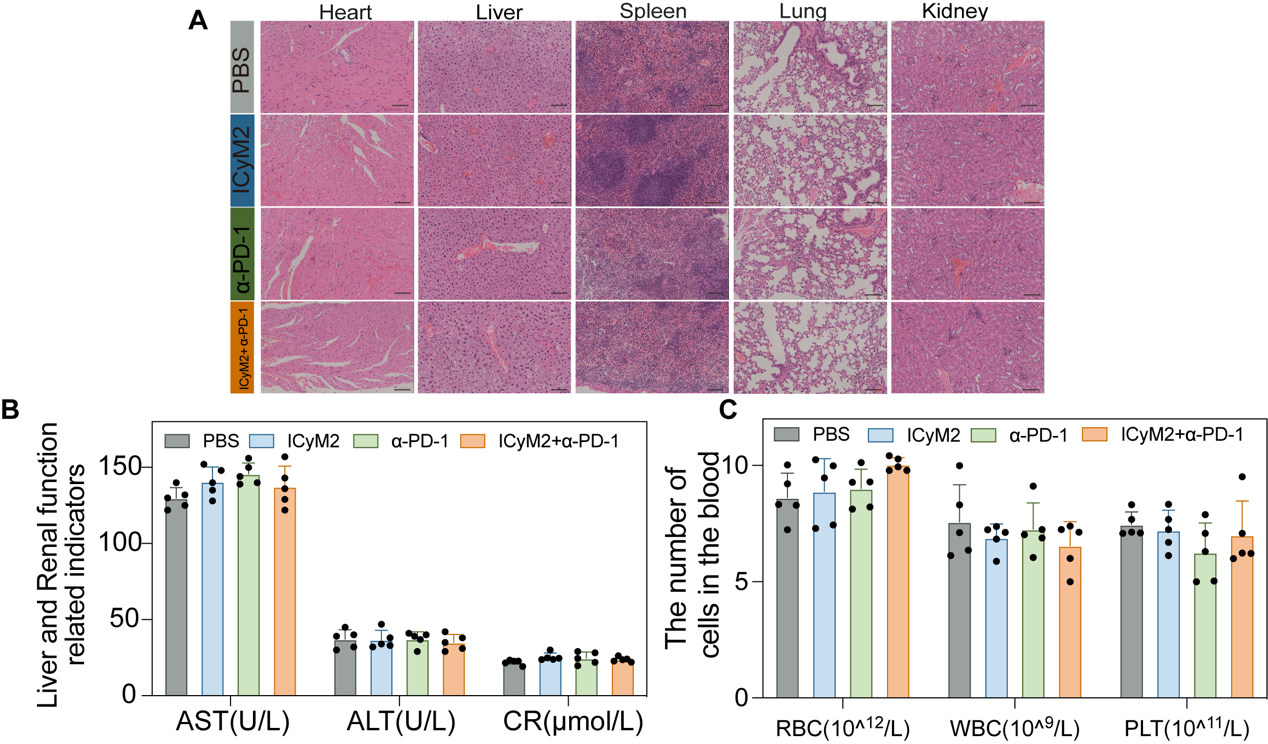


**Figure S37.** A) H&E staining of major organs of treated mice, including heart, spleen, lung, and kidney. Scale bar = 100 μm. B) liver/kidney function markers. C) Hematological parameters.


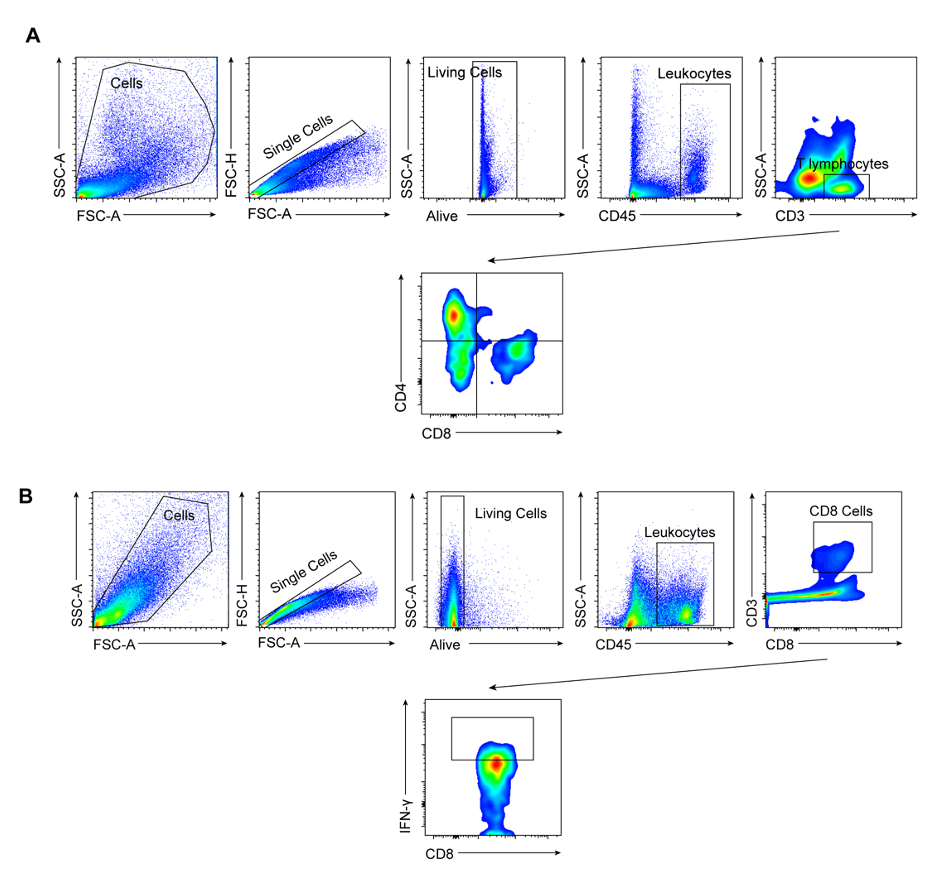


**Figure S38.** A) CD8+ T cells and CD4+ T cells were gated from CD45+ CD3+ T cells in tumors. B) IFN-γ production were gated from CD3+CD8+ cells in tumors.

**2.Experimental section**

**Patient Samples**

**Three patients diagnosed with pancreatic cancer (PC) were enrolled from the PC database of Nanjing Drum Tower Hospital, affiliated with Nanjing University Medical School. Biopsies were obtained using endoscopic ultrasound-guided fine-needle aspiration (EUS-FNA). Each procedure was performed with rapid on-site evaluation (ROSE) by a cytopathologist to confirm specimen adequacy. Tissues were fixed in 10% neutral-buffered formalin for 48 h and subsequently embedded in paraffin. Written informed consent was obtained from all participants. The study protocol was reviewed and approved by the Ethics Committee of Nanjing Drum Tower Hospital (approval no. 2020-183-01) and conducted in accordance with the Declaration of Helsinki and the International Conference on Harmonization Guidelines for Good Clinical Practice.**

**Materials and Reagents**

**MSA-2 was purchased from MedChemExpress (Shanghai, China). Collagenase IV was obtained from Yeasen Biotechnology (Shanghai, China). Red blood cell lysis buffer and Zombie-NIR™ fixable viability dye were obtained from BioLegend (San Diego, CA, USA). Antibodies for flow cytometry, including** **anti-CD45-PerCP-Cy5.5, anti-CD11b-FITC, anti-Gr1-PE, anti-CD80-PE, anti-CD86-APC, anti-CD3-FITC/PerCP-Cy5.5, anti-CD4-APC, anti-CD8a-PE, anti-CD44-FITC, anti-CD62L-APC,** **anti-IFN-γ-APC, anti-MHC-II-APC, anti-CD25-FITC, anti-FOXP3-PE, anti-F4/80-PE, anti-CD206-PE-Cy7/PE, anti-NK1.1-PE, and anti-CD11c-FITC, were purchased from BioLegend or BD Biosciences (San Jose, CA, USA).** **Primary antibodies were purchased from Abcam (Cambridge, MA, USA), including p-STING, p-TBK1, p-IRF3, STING, TBK1, IRF3 and GAPDH for western blotting; p-TBK1, p-IRF3, α-SMA, PanCK, γ-H2AX, CRT, and HMGB1 for immunofluorescence; and α-SMA and PanCK for immunohistochemistry. Fluorophore-conjugated secondary antibodies for immunofluorescence were also obtained from Abcam.**

**The ROS assay kit (DCFH-DA) and mitochondrial membrane potential assay kit (JC-1) were obtained from Beyotime Biotechnology (Shanghai, China). The Super-Rapid Protein Quantification Kit (BCA Assay), Live/Dead Cell Double Staining Kit, and CheKine™ Micro ATP Content Assay Kit were purchased from Abbkine (Wuhan, China). ELISA kits for IFN-β, CXCL10 and IL-6 detection were purchased from** **Yfxbio Biotech. Co., Ltd. (Nanjing, China). DMEM, RPMI-1640 medium, and fetal bovine serum (FBS) were purchased from Haixing Biosciences (Suzhou, China). Recombinant cytokines, including GM-CSF, IL-4, and M-CSF, were obtained from ABclonal Technology (Wuhan, China).**

**All other reagents and solvents were of analytical or HPLC grade and used without further purification.**

**Synthesis of ICyOH**

**ICy (129 mg, 0.15 mmol) was dissolved in dry dichloromethane (DCM) under an argon atmosphere, followed by the addition of HS(CH₂)₆OH (0.16 mmol) and N,N-diisopropylethylamine (DIPEA, 16 μL, 0.16 mmol). The reaction mixture was stirred overnight at room temperature, and the progress was monitored by thin-layer chromatography (TLC). Upon completion, the mixture was concentrated under reduced pressure and purified by column chromatography (CH₂Cl₂/MeOH = 9:1, v/v) to yield ICyOH as a green solid (133 mg, 92%).**

**^1^H NMR (400 MHz, DMSO-d₆) δ 8.71 (d, J = 14.2 Hz, 1H), 8.00 (d, J = 1.2 Hz, 1H), 7.76 (dd, J = 8.3, 1.3 Hz, 1H), 7.24 (d, J = 8.4 Hz, 1H), 6.28 (d, J = 14.3 Hz, 1H), 3.63 (s, 3H), 2.80 (t, J = 7.1 Hz, 1H), 2.65 (t, J = 5.3 Hz, 2H), 1.83–1.77 (m, 1H), 1.68 (s, 6H), 1.58–1.53 (m, 1H), 1.35 (dd, J = 13.0, 6.2 Hz, 2H), 1.25 (d, J = 9.0 Hz, 2H).**

**^13^C NMR (101 MHz, DMSO-d₆) δ 172.01, 156.34, 145.00, 143.67, 143.32, 137.51, 133.88, 131.49, 113.80, 102.26, 89.46, 60.97, 49.14, 37.66, 32.70, 31.85, 30.39, 28.47, 27.52, 26.16, 25.57, 21.08.**

**HRMS (ESI): calcd. for [C₃₈H₄₇I₂N₂OS]⁺ 833.1494; found 833.1467 [M]⁺.**

**Synthesis of ICyM2**

**MSA-2 (59 mg, 0.2 mmol) and HATU (114 mg, 0.3 mmol) were dissolved in anhydrous DMF under ice-bath conditions. After stirring with DIPEA (77 mg, 0.6 mmol) for 5 min, ICyOH (192 mg, 0.2 mmol) was added, and the reaction mixture was stirred overnight at room temperature. The reaction was quenched by dilution with deionized water, and the product was extracted with dichloromethane (DCM). The organic phase was dried over anhydrous Na₂SO₄, concentrated, and purified by column chromatography (CH₂Cl₂/MeOH = 9:1, v/v) to yield ICyM2 as a green solid (198 mg, 80%).**

**^1^H NMR (400 MHz, DMSO-d₆) δ 8.70 (d, J = 14.2 Hz, 1H), 8.19 (s, 1H), 8.00 (s, 1H), 7.75 (d, J = 8.3 Hz, 1H), 7.57 (s, 1H), 7.47 (s, 1H), 7.23 (d, J = 8.4 Hz, 1H), 6.27 (d, J = 14.2 Hz, 1H), 3.97 (t, J = 6.3 Hz, 1H), 3.86 (s, 2H), 3.84 (s, 2H), 3.63 (s, 3H), 3.31–3.26 (m, 1H), 2.77 (t, J = 6.9 Hz, 1H), 2.64 (d, J = 5.4 Hz, 2H), 1.83–1.76 (m, 1H), 1.68 (s, 6H), 1.51 (d, J = 5.6 Hz, 2H), 1.32 (d, J = 6.8 Hz, 1H), 1.28–1.17 (m, 2H).**

**^13^C NMR (101 MHz, DMSO-d₆) δ 192.21, 172.15, 171.53, 155.85, 150.83, 148.56, 144.52, 143.18, 142.83, 140.35, 137.01, 135.68, 133.39, 132.49, 130.99, 130.42, 113.29, 106.57, 104.24, 101.76, 88.99, 63.79, 55.88, 55.64, 54.91, 48.64, 37.11, 33.03, 31.36, 29.68, 27.74, 27.03, 25.67, 24.90, 20.58.**

**HRMS (ESI): calcd. for [C₅₂H₅₉I₂N₂O₅S₂]⁺ 1109.1949; found 1109.1914 [M]⁺.**

**Characterization of ICyOH and ICyM2**

**Spectroscopic analysis**
**ICyOH and ICyM2 were first dissolved in HPLC-grade DMSO to prepare 10 mM stock solutions, which were stored at −20 °C until use. For spectral analysis, the stock solutions were diluted with spectroscopic-grade methanol to 5 μM, and the absorption and fluorescence emission spectra were recorded at room temperature in a 1 cm quartz cuvette using a UV–Vis spectrophotometer and fluorescence spectrometer with a slit width of 5 nm.**

**Particle size, morphology, Ζeta-potential and colloidal stability**

**Dynamic light scattering (DLS, Brookhaven 90 Plus/BI-MAS, USA) and transmission electron microscopy (TEM, JEOL JEM-F200, Japan) were employed to determine the hydrodynamic size, morphology, Ζeta-potential and dispersion state of ICyOH and ICyM2. For DLS measurements, the compounds were diluted to 5 μM in deionized water and vortexed for 2 min prior to analysis. To evaluate colloidal stability, ICyM2 dispersions in PBS supplemented with 10% fetal bovine serum (FBS) were monitored over 72 h, and the particle size distribution was recorded at each time point.**

**Singlet oxygen quantum yield**

**Singlet oxygen (^1^O₂) generation was quantified using 1,3-diphenylisobenzofuran (DPBF) as a probe. Solutions of ICyOH or ICyM2 in methanol were prepared with an optical density of ~0.3 at 790 nm. DPBF was added to achieve an absorbance of ~0.6 at 410 nm. Samples were irradiated with an 808 nm laser (100 mW cm⁻²), and the decay in DPBF absorbance at 410 nm was recorded every 30 s. The singlet oxygen quantum yield (ΦΔ) was calculated according to the equation:**

**ΦΔ(S) = ΦΔ(R) ×** $\frac{S^{R}}{S^{S}}$ **​×**$\frac{F^{\mathbf{S}}}{\mathbf{F}^{R}}$

**where S is the slope of DPBF absorbance decay, F is the absorption correction factor (F=1-10^-OD808^),** **and superscripts S and R denote sample and reference, respectively. Indocyanine green (ICG, ΦΔ = 0.20 in methanol) was used as the reference.**

**Photothermal performance**

**The photothermal effect of ICyOH and ICyM2 was tested in both methanol and PBS. Samples were prepared at 50 μM, irradiated with an 808 nm laser (0.5 W cm⁻²), and the temperature increase was monitored using an infrared thermal camera (FLIR E40, USA). Temperature elevation curves were plotted to compare photothermal conversion efficiency.**

**Esterase-responsive hydrolysis
The enzymatic cleavage of ICyM2 was assessed using porcine liver esterase (PLE, 20 U/mL) in PBS at 37 °C. ICyM2 solutions (50 μM) were incubated with PLE, and 100 μL aliquots were withdrawn at predetermined time points. Each sample was quenched with 100 μL acetonitrile and analyzed by reverse-phase HPLC using acetonitrile/water as the mobile phase at a flow rate of 1.0 mL/min. UV absorbance was monitored at 360 nm and 790 nm to detect the formation of MSA-2 and ICyOH, as well as the depletion of ICyM2. Hydrolysis products were further confirmed by HRMS, where free ICyOH was identified by its characteristic m/z peak at 833.1467.**

**Cells and animals**

**Panc02 and NIH/3T3 (mouse embryonic fibroblast) cell lines were obtained from the Institute of Biochemistry and Cell Biology, Shanghai Institutes for Biological Sciences, Chinese Academy of Sciences (Shanghai, China). Cells were cultured in DMEM (high glucose, Haixing Biosciences, Suzhou, China) supplemented with 10% fetal bovine serum (FBS; Gibco, USA), 100 U/mL penicillin, and 100 μg/mL streptomycin. All cells were maintained at 37 °C in a humidified incubator with 5% CO₂ and were routinely tested to confirm the absence of mycoplasma contamination.**

**To generate cancer-associated fibroblast (CAF)-like cells, NIH/3T3 fibroblasts were incubated in Panc02-conditioned medium. Briefly, Panc02 cells were seeded in 75 cm² flasks and cultured until ~70–80% confluence, followed by replacement with fresh DMEM containing 10% FBS. After 24 h, the supernatant was collected, centrifuged at 1,000 rpm for 5 min to remove cell debris, and filtered through a 0.22 μm membrane. NIH/3T3 cells were cultured in this conditioned medium for 72 h, after which α-SMA immunofluorescence staining was performed to confirm CAF-like activation. The activated fibroblasts were subsequently used in in vitro and in vivo experiments.**

**Male immunocompetent C57BL/6 mice were purchased from GemPharmatech Co., Ltd. (Nanjing, China) and housed under specific pathogen-free (SPF) conditions. All animal procedures were conducted in accordance with protocols approved by the Institutional Animal Care and Use Committee of Nanjing Drum Tower Hospital (approval no. 20231129).**

**Dark and Light Toxicities**

**CAFs and Panc02 cells were cultured in DMEM supplemented with 10% fetal bovine serum (FBS) and 1% penicillin/streptomycin at 37 °C in a humidified 5% CO****_2_ incubator. The cytotoxicity of ICyM2 with or without near-infrared (NIR) irradiation was evaluated using a CCK-8 assay. Briefly, cells were seeded into 96-well plates at a density of 5 × 10^3^ cells per well and allowed to adhere overnight. The cells were then treated with various concentrations of ICyM2 (0.5–4 μM) for 6 h. After washing with PBS, wells assigned to the light group were irradiated with an 808 nm laser (0.5 W cm⁻^2^, 1 min), while non-irradiated wells served as the dark group. Subsequently, cells were cultured for another 24 h. Cell viability was assessed using a CCK-8 kit (TargetMol, USA; C0005) following the manufacturer’s instructions.**

**Isolation and Culture of Bone Marrow–Derived Dendritic Cells (BMDCs)**

**Bone marrow cells were harvested from the femurs and tibias of male C57BL/6 mice under aseptic conditions. The marrow was flushed with RPMI-1640 medium (Haixing Biosciences, Suzhou, China) containing 10% heat-inactivated fetal bovine serum (FBS) and 1% penicillin/streptomycin. After red blood cell lysis and washing, cells were resuspended and seeded in 24-well plates at a density of 1 × 10⁶ cells/mL. The culture medium was supplemented with recombinant mouse GM-CSF (20 ng/mL) and IL-4 (5 ng/mL) (ABclonal Technology, Wuhan, China). Cultures were maintained at 37 °C in a humidified incubator with 5% CO₂. On day 3, half of the medium was gently replaced with fresh cytokine-supplemented medium to remove nonadherent cells and debris. Semi-suspended and loosely adherent cells were collected between days 7 and 9 and used as immature BMDCs for subsequent experiments.**

**Isolation and Culture of Bone Marrow–Derived Macrophages (BMDMs)**

**Bone marrow cells were isolated as described above and seeded in 24-well plates at 1 × 10⁶ cells/mL in DMEM medium (Haixing Biosciences, Suzhou, China) containing 10% heat-inactivated FBS and 1% penicillin/streptomycin. Recombinant mouse M-CSF (20 ng/mL, ABclonal Technology, Wuhan, China) was added to induce macrophage differentiation. On day 3, the medium was replaced with fresh M-CSF–supplemented medium to remove nonadherent cells. By days 6–7, adherent, spindle-shaped cells with typical macrophage morphology were observed. Fully differentiated BMDMs were harvested and used for experiments between days 7 and 9.**

**Cellular immunofluorescence**

**NIH/3T3 fibroblasts were incubated with Panc02-conditioned medium to induce CAF-like activation, and α-SMA staining was performed to confirm the phenotype. For functional studies, CAFs or Panc02 cells were divided into three groups: PBS control, ICyM2(–) (non-irradiated), and ICyM2(+) (irradiated). Cells were treated with ICyM2 (2 µM) for 6 h, followed by near-infrared laser irradiation (808 nm, 0.5 W cm⁻², 1 min) for the ICyM2(+) group, while ICyM2(–) cells were kept in the dark. After treatment, cells were washed with PBS, fixed with 4% paraformaldehyde for 15 min, and permeabilized with 0.1% Triton X-100 for 10 min. After blocking with 3% BSA in PBS for 1 h, cells were incubated with primary antibodies against α-SMA, ecto-CRT, HMGB1, or γ-H2AX (1:1000 dilution, 4 °C overnight). Following PBS washes, cells were incubated with fluorophore-conjugated secondary antibodies (1:500 dilution, 1 h, room temperature, in the dark). F-actin was visualized with phalloidin, and nuclei were counterstained with DAPI. Images were captured using a confocal laser scanning microscope (LSM 880, Carl Zeiss, Germany).**

**Apoptosis Evaluation by Flow Cytometry**

**Panc02 cells (~5 × 10⁵) were seeded into 6-well plates and incubated at 37 °C in a 5% CO_2_ atmosphere for 24 h. Cells were then divided into three groups: PBS control, ICyM2(–) (non-irradiated), and ICyM2(+) (irradiated). For the ICyM2 treatment, cells were incubated with ICyM2 (2 μM) for 6 h. Wells assigned to the ICyM2(+) group were subsequently exposed to an 808 nm laser (0.5 W cm⁻², 1 min), while ICyM2(–) wells were kept in the dark. After treatment, cells were washed with PBS, trypsinized, collected, and resuspended in 200 μL of binding buffer. Apoptosis was detected using an Annexin V-FITC/PI apoptosis detection kit (Beyotime, Shanghai, China) according to the manufacturer’s protocol. After staining, apoptotic cell populations were analyzed on a flow cytometer (BD FACSCanto II, BD Biosciences, USA), and at least 10,000 live cells were recorded per sample.**

**Live/Dead assay**

Panc02 cells (~5 × 10⁴) were seeded into glass-bottom culture dishes and allowed to adhere for 24 h. Cells were then divided into three groups: PBS control, ICyM2(–) (non-irradiated), and ICyM2(+) (irradiated). For treatment, cells were incubated with ICyM2 (2 μM) for 6 h. Wells assigned to the ICyM2(+) group were subsequently exposed to an 808 nm NIR laser (0.5 W cm⁻², 1 min), while ICyM2(–) wells were kept in the dark. After irradiation, the medium was removed and cells were washed three times with PBS. Subsequently, 1 μM Calcein-AM and 1 μM propidium iodide (PI) were added and incubated at 37 °C for 30 min in the dark. After washing three times with PBS, fluorescence images were acquired using a confocal laser scanning microscope (LSM 880, Carl Zeiss, Germany).

**Intracellular ROS generation detection**

Panc02 cells and CAFs (~5 × 10⁵) were seeded into 6-well plates and cultured for 24 h. Cells were divided into three groups: PBS control, ICyM2(–) (non-irradiated), and ICyM2(+) (irradiated). For the ICyM2 treatment, cells were incubated with ICyM2 (2 μM) for 6 h. Wells assigned to the ICyM2(+) group were subsequently exposed to an 808 nm laser (0.5 W/cm², 1 min), while ICyM2(–) wells were kept in the dark. After treatment, cells were incubated with the ROS-sensitive probe DCFH-DA (20 μM, Beyotime, Shanghai, China) for 30 min at 37 °C in the dark, washed three times with PBS, and resuspended in staining buffer. Intracellular ROS levels were quantified using a flow cytometer (BD FACSCanto II, BD Biosciences, USA), and data were analyzed with FlowJo software (v10.0).

**Mitochondrial membrane potential (ΔΨm) detection**

Panc02 tumor cells and CAFs were divided into three groups: PBS control, ICyM2(–) (non-irradiated), and ICyM2(+) (irradiated). Cells were treated with ICyM2 (2 μM) for 6 h, followed by near-infrared (808 nm, 0.5 W cm⁻², 1 min) irradiation for the ICyM2(+) group. After treatment, cells were incubated with JC-1 working solution at 37 °C for 20 min in the dark and washed twice with PBS. The mitochondrial membrane potential (ΔΨm) was then analyzed by flow cytometry (BD FACSCalibur, BD Biosciences, USA), and representative fluorescence images were captured using a confocal laser scanning microscope (LSM 880, Zeiss, Germany).

**ATP Release Detection**

To assess ATP release, Panc02 cells were treated with PBS or ICyM2 (2 μM) for 6 hours, followed by 808 nm laser irradiation (0.5 W cm⁻², 1 min). After treatment, the supernatant was collected at 24 hours, and the ATP content was quantified using an ATP Assay Kit, according to the manufacturer's instructions.

**Transmission electron microscopy (TEM)**

CAFs and Panc02 cells were washed with PBS after treatment, fixed overnight in 2.5% glutaraldehyde, and subsequently post-fixed in 1% osmium tetroxide for 2 hours. After graded ethanol dehydration, the samples were embedded in Epon resin, sectioned, and stained with uranyl acetate and lead citrate. Ultrastructural changes were examined and imaged using a transmission electron microscope.

**Co-culture of Panc02, BMDM, and BMDC**

Panc02 cells were seeded in 6-well plates and cultured for 24 h. Cells were then divided into three groups: PBS control, ICyM2(–) (non-irradiated), and ICyM2(+) (irradiated). For treatment, cells were incubated with ICyM2 (2 μM) for 6 h. Wells assigned to the ICyM2(+) group were subsequently exposed to an 808 nm laser (0.5 W cm⁻², 1 min), while ICyM2(–) wells were kept in the dark. After treatment, bone marrow–derived macrophages (BMDMs) or dendritic cells (BMDCs) were added to the Panc02 culture system and co-incubated for an additional 24 h.

For DC maturation analysis, cells were stained with anti-CD80-PE, anti-CD86-APC, and anti-MHC-II-APC antibodies. For macrophage polarization, BMDMs were stained with anti-CD206-PE and anti-CD86-APC antibodies. Flow cytometry was performed using a CytoFlex S flow cytometer (Beckman Coulter, USA), and data were analyzed with FlowJo software (v10.6.2). The expression of phosphorylated TBK1 (p-TBK1) and IRF3 (p-IRF3) in DCs and macrophages was determined by Western blotting, and IFN-β secretion was quantified by ELISA following the manufacturer’s protocol.

**Cellular Uptake**

Panc02 tumor cells and CAFs were seeded in confocal dishes and cultured for 24 h. Cells were then incubated with ICyM2 (2 μM) for 6 h. After fixation with 4% paraformaldehyde, cells were stained with PanCK (Panc02) or α-SMA (CAFs), followed by incubation with secondary antibodies, and nuclei were counterstained with DAPI. Fluorescence images were acquired using a confocal laser scanning microscope (LSM880, Zeiss, Germany) to assess ICyM2 uptake.

**Mitochondrial Colocalization**

CAFs and Panc02 cells were seeded into 35 mm confocal dishes at a density of 1 × 10⁴ cells/mL and cultured until appropriate confluence. Cells were incubated with ICyM2 (2 μM), followed by removal of the medium and three washes with PBS. Mitochondria were then stained with PK Mito Red, and nuclei were counterstained with DAPI at the recommended concentrations. After 15 min of incubation, cells were washed three times with PBS, and fluorescence images were acquired using a confocal laser scanning microscope (LSM880, Zeiss, Germany).

**Histological, Immunohistochemical, and Immunofluorescence Staining**

Human pancreatic cancer tissues and mouse tumor samples were fixed in 10% neutral buffered formalin, paraffin-embedded, and sectioned at 4 μm. For histological assessment, sections were stained with hematoxylin and eosin (H&E) or Masson’s trichrome following standard protocols.

For immunohistochemistry (IHC), sections were deparaffinized, rehydrated, subjected to antigen retrieval, and blocked with 10% BSA. Primary antibodies against PanCK, α-SMA, and Ki67 were incubated at 4 °C overnight, followed by HRP-conjugated secondary antibodies and DAB chromogenic development.

For immunofluorescence (IF), tumor sections were similarly processed and incubated with primary antibodies against α-SMA, PanCK, p-TBK1, p-IRF3, CRT, and HMGB1 overnight at 4 °C. Alexa Fluor–conjugated secondary antibodies (1:500) were applied for 1 h at room temperature, with nuclei counterstained using DAPI. TUNEL staining was also performed to assess apoptosis in tumor sections. Images were acquired using an optical microscope or a confocal laser scanning microscope (LSM880, Zeiss, Germany).

**In Vivo Anticancer Study**

Male C57BL/6 mice were co-inoculated subcutaneously with 1 × 10⁵ Panc02 cells and 1 × 10⁵ CAFs into the right flank to establish the desmoplastic pancreatic cancer (PC) model. Tumor growth was monitored, and once tumors reached approximately 100 mm³ by day 0, the mice were randomly allocated into six treatment groups: PBS (200 μL), ICyOH + Laser (200 μL, 150 μM), MSA-2 (200 μL, 150 μM), ICyOH + MSA-2 + Laser (200 μL, 150 μM), ICyM2 (200 μL, 150 μM), and ICyM2 + Laser (200 μL, 150 μM). The treatments were administered as follows: ICyOH (2 μM) was incubated with cells for 6 hours and irradiated with 808 nm laser (0.5 W/cm², 5 min); MSA-2 (150 μM) was injected intravenously (200 μL). The treatments were repeated every 3 days for a total of 3 cycles (on days 0, 4, and 8).

Body weight and tumor volume were measured every two days. Tumor volume was calculated using the formula: Volume = (Length × Width²) / 2. On day 20, the mice were euthanized, and tumor tissues along with major organs (heart, liver, spleen, lung, and kidneys) were collected for further analysis. Tumor tissues were subjected to H&E staining, Masson’s trichrome staining, Sirius Red staining, and immunofluorescence for p-TBK1, p-IRF3, α-SMA, HMGB1, and CRT. TUNEL assay was performed to detect apoptosis.

**Anti-metastasis efficacy evaluation**

Following primary tumor treatment, Panc02 cells (1 × 10⁵ cells) were intravenously injected into the mice on days 12 and 16 to simulate aggressive metastatic dissemination. At day 30, mice were euthanized, and the lungs were carefully extracted and soaked in Bouin's solution for fixation. After fixation, the lungs were examined for metastatic nodules, and the metastatic spread was assessed by counting pulmonary metastases. For histological analysis, the lungs were processed for hematoxylin and eosin (H&E) staining to evaluate tumor metastasis. Tumor volume, body weight, and survival were monitored throughout the experiment, with survival curves generated for statistical analysis.

**In vivo PD-1 blockade therapy**

To evaluate the synergistic effect of ICyM2 and anti–PD-1 therapy, mice bearing desmoplastic Panc02 tumors were treated with anti–PD-1 monoclonal antibody (200 μg per mouse) via intraperitoneal injection (i.p.) every four days. The therapy was initiated on day 0 and continued until day 16. Tumor growth was monitored, and mice were sacrificed on day 20 for tumor analysis. During the course of the study, each group received ICyM2 treatment or PBS as a control, and tumors were irradiated with a single dose of 808 nm laser (0.5 W/cm², 5 minutes) on day 0, day 4, and day 8. Tumor volumes and body weights were recorded at regular intervals to assess the therapeutic response.

**In vivo toxicity and histological analysis**

At the conclusion of the in vivo therapeutic study, the major organs (heart, liver, spleen, kidney, and lung) from tumor-bearing mice in each group were collected for histological examination. The organs were fixed in formalin, sectioned, and stained with hematoxylin and eosin (H&E) to assess any potential pathological changes. Blood samples were also collected to evaluate liver and renal function. Liver function was assessed by measuring aspartate aminotransferase (AST) and alanine aminotransferase (ALT) levels, while renal function was evaluated by determining creatinine (CR) levels.

**In vivo evaluation of immune effector and cytokine release**

Tumor tissues were excised, minced into ~1 mm³ fragments, and enzymatically digested with 2 mg/mL collagenase IV at 37 °C for 25 min. The resulting suspensions were filtered through 70 μm strainers and subjected to red blood cell lysis to obtain single-cell suspensions. Cell viability was determined using Zombie-NIR™ Fixable Viability Dye. Following Fc receptor blocking with anti-CD16/32 for 10 min at 4 °C, cells were incubated with fluorochrome-conjugated antibodies for 30 min at 4 °C in the dark. The following antibodies were used: anti-CD45-PerCP-Cy5.5, anti-CD11b-FITC, anti-Gr1-PE, anti-CD80-PE, anti-CD86-APC, anti-CD3-FITC, anti-CD4-APC, anti-CD8a-PE, anti-CD44-FITC, anti-CD62L-APC, anti-IFN-γ-APC, anti-CD25-FITC, anti-FOXP3-PE, anti-F4/80-PE/ PE-Cy7, anti-CD206-PE-Cy7, anti-NK1.1-PE, and anti-CD11c-FITC. For intracellular markers (IFN-γ, FOXP3), fixation and permeabilization were performed using a commercial buffer set. Samples were acquired on a BD LSRFortessa™ flow cytometer, and data were analyzed with FlowJo software (version 10.6.2).

For memory T cell analysis, spleens were aseptically collected, mechanically dissociated, and filtered through 70 μm strainers to prepare single-cell suspensions. After red blood cell lysis, cells were stained with anti-CD3-PerCP-Cy5.5, anti-CD8a-PE, anti-CD44-FITC, and anti-CD62L-APC, following the same procedures described above.

Peripheral blood was collected from each group by retro-orbital bleeding. Serum was separated by centrifugation at 3000 rpm for 10 min at 4 °C and stored at –80 °C until analysis. The concentration of IFN-β and IL-6 in serum was quantified using a commercial ELISA kit according to the manufacturer’s instructions.

**Data analysis**

The value of n indicates either the number of independent experiments or the number of individual mice per experiment, with each experiment including three biological replicates. All experimental procedures were repeated at least three times. Data are presented as mean ± standard deviation (SD). Statistical analyses were performed using GraphPad Prism (version 7.0; San Diego, CA, USA). Comparisons between two groups were conducted using unpaired two-tailed Student’s t-tests, and multiple group comparisons were performed by one-way analysis of variance (ANOVA) followed by Tukey’s post hoc test. Statistical significance was defined as p < 0.05 (*p < 0.05; **p < 0.01; ***p < 0.001; ****p < 0.0001. ns, not significant).
